# Investigations towards the stereoselective organocatalyzed Michael addition of dimethyl malonate to a racemic nitroalkene: possible route to the 4-methylpregabalin core structure

**DOI:** 10.3762/bjoc.14.42

**Published:** 2018-03-05

**Authors:** Denisa Vargová, Rastislav Baran, Radovan Šebesta

**Affiliations:** 1 Department of Organic Chemistry, Faculty of Natural Sciences, Comenius University in Bratislava, Mlynská dolina, Ilkovičova 6, SK-842 15 Bratislava, Slovakia

**Keywords:** kinetic resolution, Michael addition, organocatalysis, pregabalin, squaramide

## Abstract

Chiral derivatives of γ-aminobutyric acid are widely used as medicines and can be obtained by organocatalytic Michael additions. We show here the stereoselective synthesis of 4-methylpregabalin stereoisomers using a Michael addition of dimethyl malonate to a racemic nitroalkene. The key step of the synthesis operates as a kinetic resolution with a chiral squaramide catalyst. Furthermore, specific organocatalysts can provide respective stereoisomers of the key Michael adduct in up to 99:1 er.

## Introduction

Asymmetric organocatalysis has considerably broadened possibilities for stereoselective synthesis of bioactive compounds [1–3]. In particular, stereoselective conversions of nitro compounds served in many syntheses of pharmaceuticals [4].

**Figure 1:**
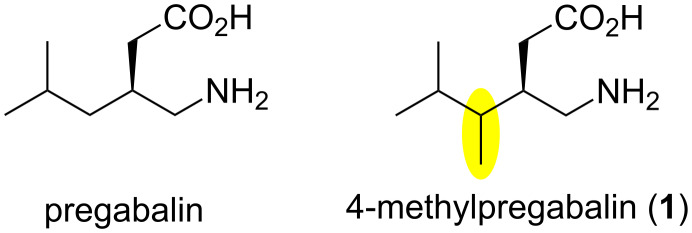
Structures of pregabalin and methylpregabalin.

Derivatives of γ-aminobutyric acid are an important class of medicines targeting problems with the central nervous system, such as pains, seizures, or epilepsy. Several mono- and dialkyl substituted derivatives, known as gabapentinoids, are currently used in clinical praxis. Important members of this class such as phenibut, gabapentin, or pregabalin have anticonvulsant, anxiolytic, and analgesic mode of actions [5]. Pregabalin (Figure 1) is one of the most widely used medicines for the treatment of neuropathic pains and partial seizures. It is also known that the (*S*)-enantiomer is approximately 10 times more active than the (*R*)-enantiomer. Medicinal properties of alkyl derivatives of pregabalin were also investigated. Wustrow and co-workers showed that the presence of another stereogenic center in the molecule and its configuration have a dramatic effect on the activity of these compounds [6]. This study also showed that 4-methylpregabalin (**1**, Figure 1) has higher activities than pregabalin (Figure 1). Methylpregabalin and related amino acids have also been investigated as treatments for ocular disorders [7]. However, the syntheses of this type of compounds were long and relied on the use of Evans chiral auxiliaries or chiral starting materials.

Various GABA derivatives were synthesized using asymmetric organocatalysis. Stereoselective Michael addition, one of the most important organocatalytic reactions [8] usually serves as a key stereoinduction transformation. Earlier studies, performed by Hayashi and Wang employed iminium activation to perform the enantioselective addition of nitromethane to enals for syntheses of baclofen and pregabalin [9–10]. Later, hydrogen-bonding activation proved to be more general for obtaining Michael adducts via the addition of 1,3-dicarbonyl compounds to nitroalkenes. Chiral thioureas and squaramides, particularly those with the bifunctional mode of action, served as excellent catalysts in numerous Michael additions, as well as other reactions [11–14]. In this way, chiral thioureas, and squaramides were used to synthesize various GABA derivatives [15–19]. Interestingly, hydrogen-bonding activation of this kind of Michael additions worked well also in aqueous media [20–21]. Other green chemistry concepts such as reusable media [22], immobilized catalysts [23], or flow set-ups have also been used with success for the synthesis of GABA derivatives [24].

Pregabalin is currently manufactured using enzymatic kinetic resolution [25], but an organocatalytic procedure based on chiral phase-transfer-catalysis of the Michael addition was also reported [26].

In this context, we decided to develop a synthesis of 4-methylpregabalin from a simple and achiral starting material and build its chirality centers using asymmetric catalysis. This paper describes the synthesis of 4-methylpregabalin from ethyl 3-methylbutanoate using an organocatalytic Michael addition as the stereoinduction step.

## Results and Discussion

**Scheme 1:**
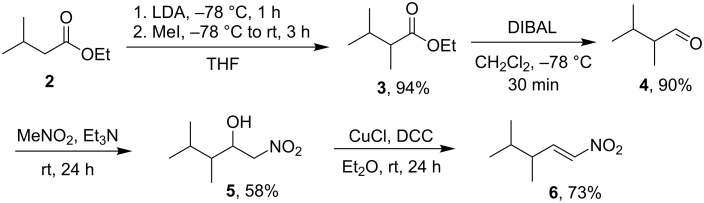
Synthesis of the nitroalkene **6**.

The starting material for the Michael addition was synthesized from ethyl 3-methylbutanoate (**2**). This straightforward sequence comprised methylation, reduction, nitro-aldol reaction, and dehydration (Scheme 1). Methylation of the ester **2** in the alpha position proceeded easily with LDA as a base and methyl iodide as an alkylating agent. The ester functionality was then reduced with DIBAL in dichloromethane to afford aldehyde **4** in 90% yield. A base-mediated addition of nitromethane to the aldehyde **4** provided nitro alcohol **5**. The somewhat lower yield (58%) of the aldol product **5** is likely caused by the reversibility of the nitro-aldol reaction. The yield of this reaction did not improve with longer time and unreacted aldehyde was still present in the reaction mixture. Nitroaldol product **5** was then dehydrated using the CuCl/DCC protocol [16] to nitroalkene **6**. Overall, this sequence afforded the desired racemic Michael acceptor **6** in total 36% yield over four steps.

**Scheme 2:**
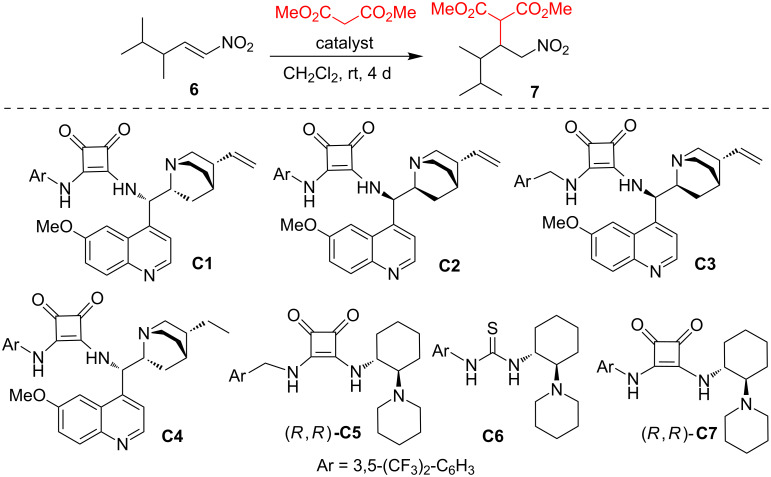
Catalyst screening in the Michael addition of dimethyl malonate to nitroalkene **6**.

**Scheme 3:**
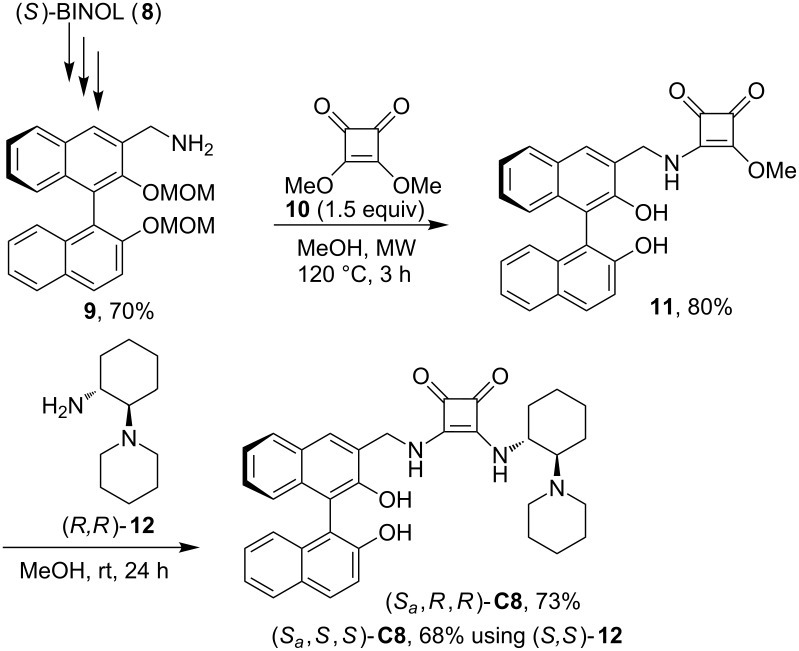
Synthesis of catalysts (*S_a_,R,R*)-**C8** and (*S_a_,S,S*)-**C8**.

With Michael acceptor **6** in hands, we started to investigate the 1,4-addition of dimethyl malonate catalyzed by hydrogen-bond-donating organocatalysts (Scheme 2). We have also briefly investigated Meldrum´s acid as a donor, instead of dimethyl malonate, but we have obtained a complicated reaction mixture, which was difficult to purify. Therefore, we have focused our attention on the Michael addition of dimethyl malonate. An initial catalyst screening was performed in dichloromethane, based on our previous experiences with this type of Michael additions [18]. We have employed a range of squaramide and thiourea organocatalysts **C1**–**C7** [18,27–34], as well as two newly synthesized binaphthol-squaramide catalysts (*S_a_,R,R*)-**C8**, and (*S_a_,S,S*)-**C8** (Scheme 3). Results of the initial catalyst screening are summarized in Table 1.

The binaphthyl structural motif has already been employed in the hydrogen-bond-donating organocatalysis [35–36]. However, the binaphthol moiety possessing an additional hydrogen bond donor group was not tested in too much depth. Therefore, we have synthesized two binaphthol-based diastereomeric squaramide catalysts (*S_a_,R,R*)-**C8** and (*S_a_,S,S*)-**C8** (Scheme 3). Starting from (*S*)-BINOL (**8**), amine **9** was obtained in five steps following literature procedures (see Supporting Information File 1 for more details) [37–40]. The amine **9** was then attached to a squaramide moiety using dimethyl squarate (**10**) in slight excess to ensure monosubstitution. In this way compound **11** was isolated in 80% yield. We have originally envisaged the transformation from amine **9** to compound **11** in two steps. However, under microwave heating conditions, both couplings of the amino group with squarate, as well as methoxymethyl (MOM) deprotection was observed. To the best of our knowledge, there is no report describing methanolysis of MOM-groups under microwave conditions. This transformation is perhaps similar to acetal hydrolysis in neat water under microwave irradiation [41]. Because of this efficient process, compound **11** was isolated in one synthetic operation in 80% yield from amine **9**. In the last step, precursor **11** reacted with either amine (*R,R*)-**12** or (*S,S*)-**12**, to afford diastereoisomeric catalysts (*S_a_,R,R*)-**C8**, and (*S_a_,S,S*)-**C8**, respectively.

In the Michael addition of dimethyl malonate to the racemic nitroalkene **6**, the cinchona-based catalysts **C1**–**C3** performed poorly and provided the desired Michael adduct **7** in less than 10% yields (Table 1, entries 1–3). The lower reactivity of the nitroalkene **6** compared with that of similar Michael acceptors is probably due to the higher steric hindrance near the β-carbon caused by branching of the alkyl chain. More relevant results in the Michael addition were obtained with more reactive, and likely less hindered, catalysts **C5**–**C8,** which afforded adduct **7** in up to 30% yield (Table 1, entries 4–9).

**Table 1:** Catalyst screening in the addition of dimethyl malonate to nitroalkene **6**.^a^

entry	catalyst	yield of **7** (%)	dr^b^	er^c^

1	**C1**	10	n.d.	n.d.
2	**C2**	<5	n.d.	n.d.
3	**C3**	0	0	0
4	(*R,R*)-**C5**	21	74:26	92:8
5	(*S,S*)-**C5**	28	98:3	6:94
6	**C6**	30	88:12	71:29
7	(*R,R*)-**C7**	22	77:23	95:5
8	(*Sa,R,R*)-**C8**	27	86:14	90:10
9	(*Sa,S,S*)-**C8**	12	53:47	1:99

^a^General conditions: nitroalkene **6** (1.4 mmol), dimethyl malonate (1.4 mmol), catalyst (0.07 mmol, 5 mol %) in CH_2_Cl_2_ (1.5 mL) was stirred at rt for 4 d; ^b^dr determined by chiral HPLC; ^c^er for the major diastereomer determined by chiral HPLC.

As the results of the Michael addition in dichloromethane were not fully satisfactory with neither of the nine tested catalysts, we have decided to test the reaction in other solvents. Using catalyst (*S,S*)-**C5**, we have evaluated the Michael addition of dimethyl malonate to nitroalkene **6** in several other solvents (Table 2). The best results were obtained in toluene. We have observed a dramatic increase of the yield of compound **7**. Its enantiomeric purity was also the highest (er 99:1) in toluene. Similarly, high enantiomeric purities were also observed in acetonitrile and methanol, but yields were lower in these solvents. This transformation likely operates as a kinetic resolution that creates a further stereocenter. We have also recovered unreacted nitroalkene **6** with enantiomeric purity of approx. 67:33 er. Given the recent successful Michael additions to nitroalkenes in aqueous media [20–21], we have also tested the Michael addition of dimethyl malonate to nitroalkene **6** in brine. For this experiment, we have employed the more lipophilic organocatalyst **C4**, but the desired adduct **7** was formed only in small amount (14% determined by NMR).

**Table 2:** Solvent screening in the addition of dimethyl malonate to nitroalkene **6**.^a^

solvent	catalyst	yield (%)	dr^b^	er^c^

CH_2_Cl_2_	(*S,S*)-**C5**	28	98:2	94:6
toluene	(*S,S*)-**C5**	75	68:32	>99:1
toluene	(*S_a_,S,S*)-**C8**	36	62:38	84:15
MeCN	(*S,S*)-**C5**	29	71:29	>99:1
Me-THF	(*S,S*)-**C5**	31	76:24	97:3
MeOH	(*S,S*)-**C5**	37	73:27	>99:1
brine^d^	**C4**	14	n.d.	n.d.

^a^General conditions: nitroalkene **6** (1.4 mmol), dimethyl malonate (1.4 mmol), catalyst (0.07 mmol, 5 mol %) in solvent (1.5 mL) was stirred at rt for 4 d; ^b^dr determined by chiral HPLC; ^c^er for the major diastereomer determined by chiral HPLC; ^d^reaction time 48 h.

**Figure 2:**
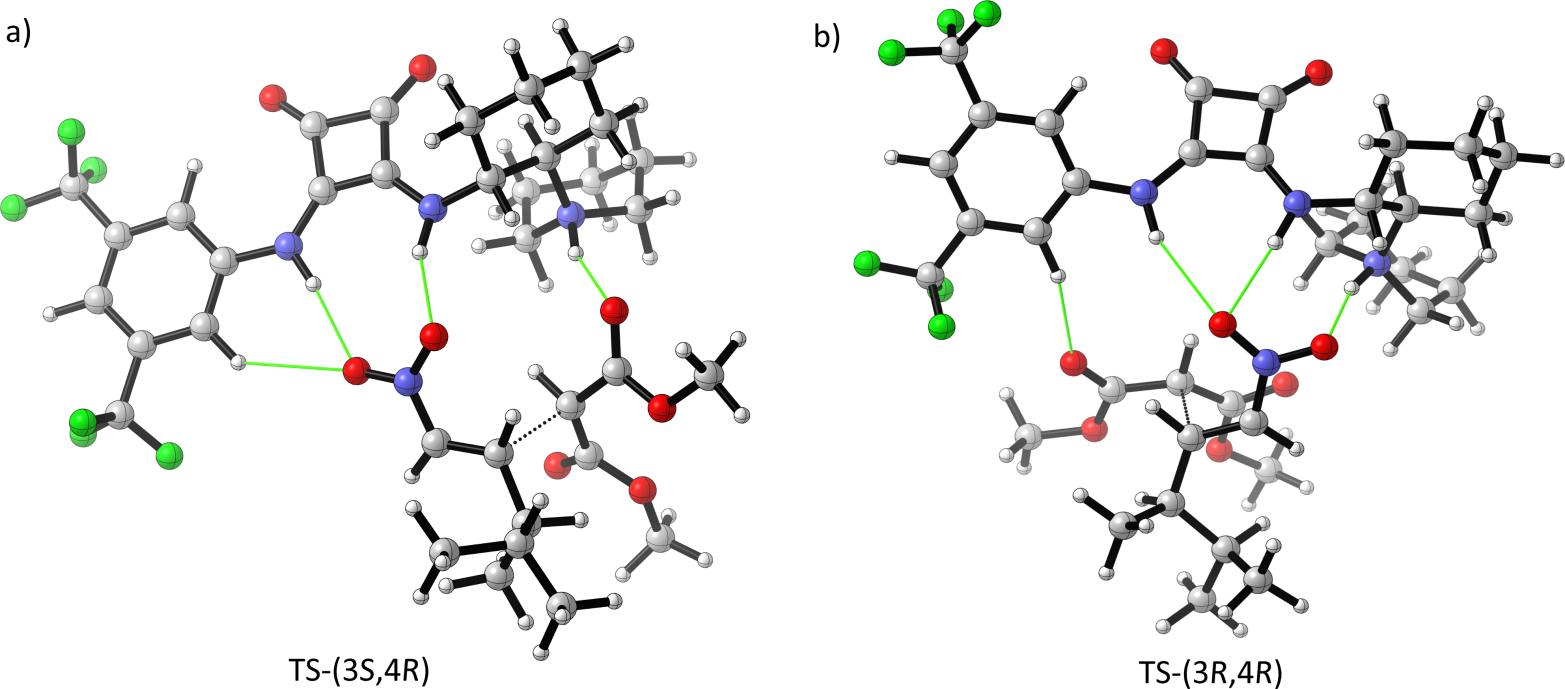
Transition state models for the reaction of (*R*)-**6** with dimethyl malonate using catalyst **C7** (M06-2X/6-311+G**-C-PCM(toluene)//HF/6-31G*).

Using quantum chemical calculations, we propose approximate transition state models for the stereoselective Michael addition (Figure 2). Geometrical optimizations were performed at HF/6-31G* level and energies were further refined using M06-2X functional with 6-311+G** basis set. Solvation effects were evaluated by using conductor-like C-PCM model with toluene as solvent (see Supporting Information File 1 for more details). The model envisages dual activation mode originally suggested by Takemoto [42], and then applied in similar situations [18,43–44]. In this model, the nitroalkene is bound to the catalyst via a squaramide moiety including an ancillary C–H···O hydrogen bond [45–46]. The dimethyl malonate anion binds via the protonated tertiary amide group. The calculations suggest that using organocatalyst **C7** the preferred enantiomers of the product, within *like* and *unlike*-diastereomers, have (3*R*,4*R*) and (3*S*,4*R*)-configuration. These isomers would result from the *Re*-face and *Si*-attack of dimethyl malonate anion to (*R*)-enantiomer of the nitroalkene **6**.

**Scheme 4:**
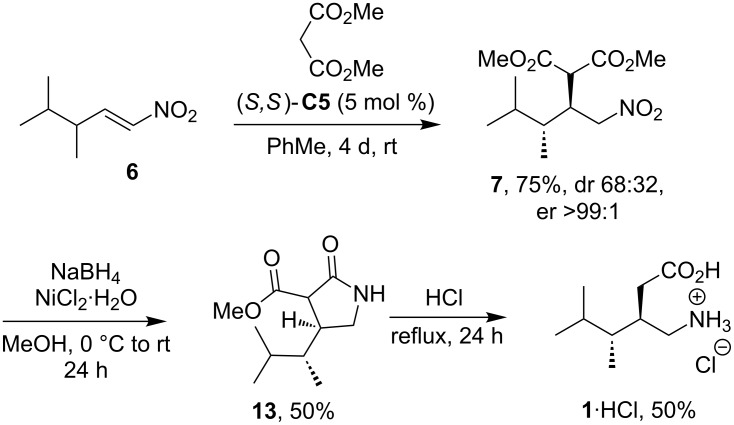
Synthesis of 4-methylpregabalin (**1**).

Enantiomerically enriched Michael adduct **7**, which was obtained with catalyst (*S*,*S*)-**C5**, was also used to finish the synthesis of 4-methylpregabalin (**1**) in an analogy to literature procedures (Scheme 4) [47]. Adduct **7** was transformed to lactone **13** via nitro group reduction with NaBH_4_/NiCl_2_ followed by spontaneous lactamization. In the last step, lactam **13** was hydrolyzed and decarboxylated to give 4-methylpregabalin hydrochloride (**1**·HCl). We have measured specific optical rotation for compound **1** to be [α]_D_^20^ −2.6 (*c* 0.95, MeOH). This value corresponds better with literature data, which for the (3*R*,4*R*)-isomer states [α]_D_^20^ −5.3 whereas for (3*R*,4*S*) [α]_D_^20^ +14.9.

## Conclusion

We have shown that 4-methylpregabalin stereoisomers can be synthesized from ethyl 3-methylbutanoate. The key step is an organocatalytic Michael addition of dimethyl malonate to racemic nitroalkene **6**. Using chiral squaramide organocatalyst, the desired Michael adduct **7** was obtained in 75% yield as a mixture of diastereomers (dr 68:32) with very high enantiomeric purity of the major diastereomer (er 99:1). With the help of quantum-chemical calculations, we have proposed a transition state model for the Michael addition.

## Supporting Information

File 1 Experimental procedures, characterization data for all compounds, pictures of NMR spectra and computational details.
