# Supplementary material for: Investigations towards the stereoselective organocatalyzed Michael addition of dimethyl malonate to a racemic nitroalkene: possible route to the 4-methylpregabalin core structure
Source: Beilstein J Org Chem. 2018 Mar 5;14:553–9. doi: 10.3762/bjoc.14.42 (PMC5852648; doi:10.3762/bjoc.14.42)
Supplement: File 1 — Experimental procedures, characterization data for all compounds, pictures of NMR spectra and computational details. [file Beilstein_J_Org_Chem-14-553-s001.pdf]

Supporting Information

for

Investigations towards the stereoselective organocatalyzed Michael addition of dimethyl malonate to a racemic nitroalkene: possible route to the 4-methylpregabalin core structure

Denisa Vargová, Rastislav Baran and Radovan Šebesta*

Address: Department of Organic Chemistry, Faculty of Natural Sciences, Comenius University in Bratislava, Mlynská dolina, Ilkovičova 6, SK-842 15 Bratislava, Slovakia

Email: Radovan Šebesta - radovan.sebesta@uniba.sk

*Corresponding author

Experimental procedures, characterization data for all compounds, pictures of NMR spectra and computational details

Contents

Experimental procedures for starting materials and intermediates in the catalysts syntheses....... S2

- General S2
- Synthesis of 4-methylpregabalin S2
- Synthesis of the quinine-derived catalysts S4
- Synthesis of the cyclohexanediamine-derived catalysts S9
- Synthesis of the binaphtol-derived catalysts S11
- Copies of NMR spectra S15
- HPLC chromatograms S41
- Computational details S44

Experimental procedures for starting materials and intermediates in the catalysts syntheses

General

Chemicals were purchased and used without further purification. Solvents were dried according to standard procedures and distilled freshly prior to use. Dry DMF was purchased from Sigma-Aldrich. Moisture sensitive reactions were performed in an Ar atmosphere, in oven-dried glassware. Reactions were monitored by TLC on Silica Gel 60 F254 (Merck). For visualization UV light (254 nm), or $KMnO_4$ solution was used. Column and flash chromatography were performed using SiO_2 (40–65 µm). NMR spectra were measured using a Varian NMR System 300 and 600, at 20 °C, using TMS as an internal standard. Abbreviations for multiplicities are: br (broad), s (singlet), d (dublet), t (triplet), q (quartet), p (pentet), m (multiplet). Melting points were measured using a Melting Point M-656 apparatus from Büchi. UV–vis spectra were measured at room temperature, on a Jenway 6705 UV–vis Spectrophotometer. IR spectra were measured using an Agilent technologies Cary 630 FTIR in dry film. Enantiomeric excesses were determined using HPLC, on Daicel Chiralpak OD-H and IC column using an UV detector. Optical activity was measured on a Jasco P-2000 polarimeter. HRMS was measured using an Orbitrap Elite Thermo Scientific Velos Pro, ionisation mode: HESI heated electrospray, MS was measured using an Agilent technologies 1200 Series, ionisation mode: ESI.

Synthesis of 4-methylpregabalin

Ethyl 2,3-dimethylbutanoate (3)

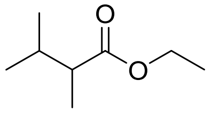

Compound **3** was prepared according to the procedure described in the literature.[1] Pale yellow oil. 1H NMR (300 MHz, $CDCl_3$) δ 4.20 – 4.03 (m, 2H), 2.20 (p, J = 7.0 Hz, 1H), 1.98 – 1.80 (m, 1H), 1.24 (t, J = 7.1 Hz, 3H), 1.09 (d, J = 7.0 Hz, 3H), 0.90 (dd, J = 6.8, 4.3 Hz, 3H). IR $\bar{\upsilon}_{max}$ (cm-1): υ 1667 (s, C=O), 1447 (m, C-H), 1235 (s, C-O), 1198 (s, C-O), 1144 (s, C-O). Spectral data agree with data described in the literature.[1]

2,3-Dimethylbutanal (4)

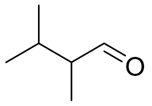

Ethyl 2,3-dimethylbutanoate (3.00 g, 23.1 mmol) was dissolved in CH_2Cl_2 (24 mL) and cooled to –78 ˚C. DIBAL (1 M in hexane, 27.7 mmol, 27.7 mL) was added dropwise. After 30 min of stirring the reaction was quenched with MeOH (6 mL). Saturated aqueous solution of Rochelle´s salt (potassium sodium tartrate tetrahydrate, 150 mL) was added, and the mixture was stirred overnight. The layers were separated, and the organic phase was extracted with *n*-pentane (3 × 50 mL). The organic layers were combined and dried over anhydrous Na_2SO_4, filtered through Celite. The product was obtained after evaporation (2.85 g, 95%). Pale yellow oil. ^{1}H NMR (300 MHz, $CDCl_3$) δ: 9.66 (d, J = 2.1 Hz, 1H), 2.25 – 2.13 (m, 1H), 2.13 – 2.00 (m, 1H), 1.00 (dd, J = 12.1, 6.9 Hz, 6H), 0.90 (d, J = 6.7 Hz,

[1] Nitsch, D.; Huber, S. M.; Pöthig, A.; Narayanan, A.;. Olah, G. A; Prakash, G. K. S.; Bach, T. *J. Am. Chem. Soc.* **2014**, *136*, 2851-2857.

3H). IR (ATR) ν: 2958 (m, =C-H), 2873 (m, =C-H), 1728 (s, C=O), 1035 (s) cm^{-1}. Spectral data agree with data described in the literature.[2]

(*E*)-3,4-Dimethyl-1-nitropent-1-ene (6)

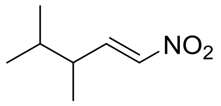

2,3-Dimethylbutanal (**4**, 500 mg, 4.99 mmol) was stirred with nitromethane (0.4 mL, 457 mg, 7.49 mmol) and triethylamine (0.1 mL, 100 mg, 0.998 mmol) 24 h at room temperature. The reaction mixture was acidified with 2 M HCl (3 mL) and extracted with CH_2Cl_2 (3 × 30 mL). Organic layers were combined and washed with water (3 × 30 mL), brine (30 mL) and dried over anhydrous Na_2SO_4. The solvent was evaporated to give the crude alcohol **5** (466 mg, 58%). This was used in the next step without further purification. Yellow oil. A mixture of diastereoisomers, peaks for minor diastereoisomer are denoted with an asterisk. ^{1}H NMR (300 MHz, $CDCl_3$) δ 4.65 – 4.33 (m, 2H), 2.57 *, 2.40 (s, 1H), 2.01 – 1.88* (m, 1H), 1.67 (tt, *J* = 13.0, 6.6 Hz, 1H), 1.59 – 1.45 (m, 1H), 1.41 – 1.23 (m, 1H), 1.02 – 0.80 (m, 9H). IR $\bar{\upsilon}_{max}$ (cm-1): υ 3392 (s, O-H), 2960 (m, C-H), 2874 (m, C-H), 1552 (s, N-O), 1382 (m, N-O), 1018 (m) cm^{-1}. Crude alcohol **5** (460 mg, 2.86 mmol) was dissolved in anhydrous Et_2O (1.4 ml), CuCl (6.0 mg, 0.057 mmol) and DCC (619 mg, 3.00 mmol) was then added. The mixture was stirred for 24 h in the absence of light at room temperature. Then it was cooled to 0 ˚C, *n*-pentane (4 mL), H_2O (2 mL) and acetic acid (1 mL) was then added, and stirred for 4 h at room temperature. The precipitate was filtered and washed with a small amount of *n*-pentane. The filtrate was washed with H_2O, saturated NH_4Cl solution, and brine. The organic layer was separated and dried over anhydrous Na_2SO_4, the solvent was evaporated. The product was obtained after distillation at reduced pressure (300 mg, 73 %). Pale yellow oil. bp 120 °C (0.89 mbar). UV λ_{max}: 233 nm. (hex:*i*PrOH, 95:5). ^{1}H NMR (300 MHz, $CDCl_3$) δ: 7.23 (dd, *J* = 13.4, 8.7 Hz, 1H), 6.94 (d, *J* = 13.5 Hz, 1H), 2.38 – 2.13 (m, 1H), 1.86 – 1.57 (m, 1H), 1.09 (d, *J* = 6.8 Hz, 3H), 0.92 (d, *J* = 6.8 Hz, 3H), 0.90 (d, *J* = 6.8 Hz, 3H).^{13}C NMR (151 MHz, $CDCl_3$) δ: 146.5, 139.0, 39.7, 32.6, 19.7, 19.6, 16.1. IR (ATR) ν: 2962 (m, C-H), 2875 (w, C-H), 1644 (w, C=C), 1522 (s, N-O), 1348 (s, N-O) cm^{-1}. HRMS (*m/z*): $[M+H]^+$ calcd for $C_7H_{13}NO_2$, 144.0982; found 144.1019.

Dimethyl (3,4-dimethyl-1-nitropentan-2-yl)propanedioate (7)

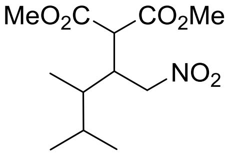

(*E*)-3,4-Dimethyl-1-nitropent-1-ene (**6**, 200 mg, 1.40 mmol), dimethylmalonate (184 mg, 1.40 mmol) and catalyst (*S*,*S*)-**C5** (35 mg, 0.069 mmol), were dissolved in PhMe (1.5 mL) and was stirred for 4 d at room temperature. The solvent was evaporated and the crude product was purified by column chromatography (SiO_2, hexane/EA 5:1). The compound **7** was isolated as a colorless oil (288 mg, 75 %). Major diastereoisomer: ^{1}H NMR (300 MHz, $CDCl_3$) δ: 4.65 (dd, C*H*HNO$_2$, *J* = 14.7, 5.6 Hz, 1H), 4.50 (dd, CH*H*NO$_2$, *J* = 14.7, 4.6 Hz, 1H), 3.76 (s, -OMe, 3H), 3.73 (s, -OMe, 3H), 3.70 (d, C*H*(CO$_2$Me)$_2$, *J* = 6.8 Hz, 1H), 3.15 (dq, C*H*CH$_2$NO$_2$, *J* = 11.4, 5.7 Hz, 1H), 1.64 (td, C*H*Me$_2$, *J* = 13.1, 6.5 Hz, 1H), 1.42 (dq, C*H*Me, *J* = 13.3, 6.7 Hz, 1H), 0.98 (d, CH*Me*Me, *J* = 6.7 Hz, 3H), 0.88 (d, CHMe*Me*, *J* = 6.7 Hz, 3H), 0.81 (d, CH*Me*, *J* = 7.0 Hz, 3H). ^{13}C NMR (75 MHz, $CDCl_3$) δ 168.7, 168.4, 75.2, 52.9, 52.7, 40.6, 39.1, 29.9, 21.2, 17.9, 11.2. Minor diastereoisomer: ^{1}H NMR (600 MHz,

[2] Daw, G.; Regan, A. C.; Watt, C. I. F.; Wood, E. *J. Phys. Org. Chem*. **2013**, *26*, 1048-1057.

$CDCl_3$) δ 4.86 (dd, C*H*HNO$_2$, *J* = 14.4, 3.7 Hz, 1H), 4.64 (dd, CH*H*NO$_2$, *J* = 9.3, 5.0 Hz, 1H), 3.77 (s, -OMe, 3H), 3.75 (s, -OMe, 3H), 3.69 (d, C*H*($CO_2Me)_2$, *J* = 3.5 Hz, 1H), 3.14 – 3.10 (m, C*H*CH_2NO_2, 1H), 1.69 (td, C*H*Me_2, *J* = 13.4, 6.8 Hz, 1H), 1.41 – 1.38 (m, C*H*Me, 1H), 0.95 (d, CH*Me*Me, *J* = 6.7 Hz, 3H), 0.86 (d, CHMe*Me*, *J* = 6.6 Hz, 3H), 0.82 (d, CH*Me*, *J* = 7.0 Hz, 3H). ^{13}C NMR (75 MHz, $CDCl_3$) δ 167.7, 167.3, 74.2, 51.9, 51.7, 39.6, 38.1, 30.9, 28.9, 28.30, 16.9, 10.2. Chiral HPLC (Daicel Chiralpac OD-H) hexane/2-propanol (90:10), flowrate 1.0 mL/min; UV (hexane/2-propanol) λ_{max} (nm) = 211, major: t_{R1} = 6.31 min; t_{R2}=11.88 min; minor: t_{R1}=6.34 min; t_{R2}=12.37 min. HRMS (*m/z*): [M + H]$^+$ calcd for $C_{12}H_{21}NO_6$, 276.1402; found 276.1442.

4-Methylpregabalin hydrochloride (1·HCl)

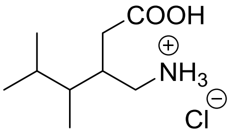

Nitroester **7** (260 mg, 0.944 mmol) and $NiCl_2{\cdot}H_2O$ (224 mg, 0.944 mmol) was dissolved in MeOH (4 mL). The solution was cooled to 0°C, and $NaBH_4$ was added (230 mg, 6.08 mmol) in small portions. The reaction mixture was stirred at rt for 3 h. The solution was cooled to 0 °C, and another portion of $NaBH_4$ was added (230 mg, 6.08 mmol) in small portions, and stirred at rt over night. The reaction was quenched with saturated NH_4Cl, filtered, and the filtrate was extracted with DCM, dried over anhydrous Na_2SO_4 and the solvent was evaporated. The crude lactam **13** was dissolved in 20% HCl (2mL) and heated at reflux for 24 h. After cooling to rt the reaction mixture was evaporated and washed with Et_2O (5 mL). The product was dissolved in MeOH (3 mL), and passed through Amberlyst 15 (250 mg) multiple times. The column was then washed with MeOH a few times until neutral pH. The column was then washed with 10% NH_3 in MeOH (2 mL) multiple times. MeOH was evaporated to give 4-methylpregabalin as a white solid (29 mg, 25%). $[\alpha]^{20}_D$ -2.6 (c 0.95, MeOH). ^{1}H NMR (300 MHz, CD_3OD) δ: 3.59 – 3.39 (m, 1H), 3.04 (dd, *J* = 25.2, 15.9 Hz, 1H), 2.78 (dd, *J* = 29.3, 19.3 Hz, 1H), 2.37 (ddd, *J* = 28.5, 15.2, 8.9 Hz, 1H), 2.17 – 2.00 (m, 1H), 1.71 (dd, *J* = 21.1, 14.2 Hz, 1H), 1.48 – 1.29 (m, 2H), 1.03 – 0.73 (m, 6H). Spectral data agree with data described in the literature.[3]

Synthesis of the quinine-derived catalysts

3,4-Dimethoxycyclobut-3-ene-1,2-dione (10)

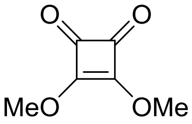

Compound **10** was prepared according to the procedure described in the literature.[4] White solid. ^{1}H NMR (300 MHz, $CDCl_3$) δ 4.38 (s, 6 H). Spectral data are in agreement with data described in the literature.[4] IR $\bar{\upsilon}_{max}$ (cm^{-1}): υ 2963 (m, C-H), 1810 (m), 1720 (s, C=O), 1582 (s, C-H), 1479 (s, C=C) , 1414 (m, C=C), 1353 (m, C-O), mp 51.0-53.1˚C.

[3] Belliotti, T. R.; Capiris, T.; Ekhato, I. V.; Kinsora, J. J.; Field, M. J.; Heffner, T. G.; Meltzer, L. T.; Schwarz, J. B.; Taylor, C. P.; Thorpe, A. J.; Vartanian, M. G.; Wise, L. D.; Zhi-Su, T.; Weber, M. L.; Wustrow, D. J. *J. Med. Chem.* **2005**, *48*, 2294-2307.
[4] Liu, H.; Tomooka,C.S.; Xu, S.L.; Yerxa, B.R.; Sullivan, R.W.; Xiong, Y.; Moore, H.W. *Org. Synth.* **1999**, *76*, 189.

3-((3,5-Bis(trifluoromethyl)phenyl)amino)-4-methoxycyclobut-3-ene-1,2-dione (S1)

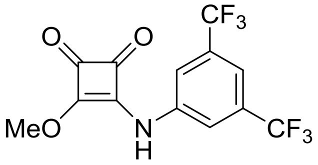

Compound **S1** was prepared according to the procedure described in the literature.[5] White solid. ^{1}H NMR (600 MHz, DMSO-d_6) δ 11.20 (s. 1H), 8.03 (s, 2H), 7.77 (s, 1H), 4.40 (s, 3H). Spectral data agree with data described in the literature.[5] IR $\bar{\upsilon}_{max}$ (cm^{-1}): υ 3186 (w, N-H), 3100 (w, N-H), 3032 (w, Ar C-H), 2974 (w, Ar C-H), 1793 (w, C=O), 1715 (w, C=O), 1579 (m, C=C), 1525 (m, C=C), 1443 (m, C-F), 1377 (m, C-F), 1270 (m, C-F), 1127 (m, C-O), mp = 178.2-178.4 ˚C.

3-((3,5-bis(trifluoromethyl)benzyl)amino)-4-methoxycyclobut-3-ene-1,2-dione (S2)

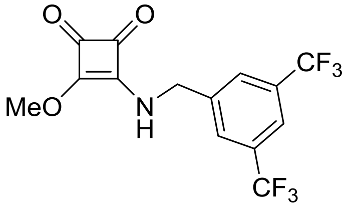

Compound **S2** was prepared according to the procedure described in the literature.[6] White solid. ^{1}H NMR (300 MHz, DMSO-d_6) δ 9.14 (d, J = 59.8 Hz, 1H), 8.05 (s, 3H), 4.78 (d, J = 55.8 Hz, 2H), 4.28 (s, 3H). Spectral data agree with data described in the literature.[6] mp = 122.4-123.7 ˚C.

9-Amino-9-deoxyepiquinine (S3)

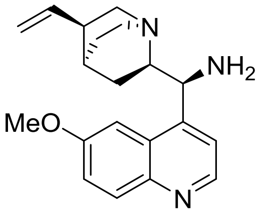

Compound **S3** was prepared according to the procedure described in the literature.[7] Brown oil. 1H NMR (300 MHz, $CDCl_3$) δ 8.75 (d, J = 4.6 Hz, 1H), 8.04 (d, J = 9.2 Hz, 1H), 7.64 (s, 1H), 7.47 (d, J = 4.3 Hz, 1H), 7.39 (dd, J = 9.2, 2.7 Hz, 1H), 5.81 (ddd, J = 17.5, 10.3, 7.5 Hz, 1H), 5.08 – 4.93 (m, 2H), 4.61 (d, J = 10.0 Hz, 1H), 3.98 (s, 3H), 3.29 (dd, J = 13.8, 10.1 Hz, 1H), 3.27 – 3.02 (m, 2H), 2.90 – 2.74 (m, 2H), 2.30 (s, 1H), 1.69 – 1.52 (m, 3H), 1.51 – 1.37 (m, 1H), 0.77 (dd, J = 13.6, 7.4 Hz, 1H). Spectral data agree with data described in the literature.[7] IR $\bar{\upsilon}_{max}$ (cm^{-1}): υ 3360 (N-H) (s), 2933 (Ar C-H) (s), 1619 (C=C) (m), 1508 (m), 1229 (C-O) (m).

[5] Yang, W.; Du, D.M. *Org. Lett.* **2010**, *12*, 5450-5453.
[6] Malerich, J. P.; Hagihara, K.; Rawal, V. H. *J. Am. Chem. Soc.* **2008**, *130*, 14416-14417.
[7] Genoni, A.; Benaglia, M.; Mattiolo, E.; Rossi, S.; Raimondi, L.; Barrulas, P. C.; Burke, A. J. *Tetrahedron Letters* **2015** , *56,* 5752 – 5756.

9-Amino(9-deoxy)epiquinidine (S4)

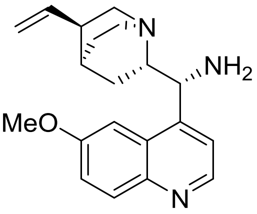

Compound **S4** was prepared according to the procedure described in the literature.[7] Brown oil. ^{1}H NMR (300 MHz, $CDCl_3$) δ 8.75 (d, *J* = 4.6 Hz, 1H), 8.03 (d, *J* = 9.2 Hz, 1H), 7.60 (br s, 1H), 7.53 (d, *J* = 4.1 Hz, 1H), 7.38 (dd, *J* = 9.2, 2.7 Hz, 1H), 5.89 (ddd, *J* = 17.2, 10.7, 6.5 Hz, 1H), 5.09 (dt, *J* = 7.5, 1.5 Hz, 1H), 5.05 (d, *J* = 1.4 Hz, 1H), 4.68 (br d, *J* = 9.9 Hz, 1H), 3.97 (s, 3H), 3.11 – 2.88 (m, 5H), 2.28 (dtd, *J* = 8.0, 6.7, 1.5 Hz, 1H), 1.95 (br s, *J* = 20.0 Hz, 2H), 1.60 (d, *J* = 10.0 Hz, 1H), 1.55 (td, *J* = 7.7, 2.8 Hz, 2H), 1.12 (dd, *J* = 8.7 Hz, 1H), 0.99 – 0.81 (m, 1H). Spectral data agree with data described in the literature.[7] IR $\bar{\upsilon}_{max}$ (cm^{-1}): υ 3360 (N-H) (s), 2935 (Ar C-H) (s), 2867 (Ar C-H) (m), 1620 (C=C) (m), 1507 (m), 1227 (C-O) (m). $[\alpha]_D^{20}$ = + 59.5 (*c* 1.0, $CHCl_3$).

Hydroquinine (S5)

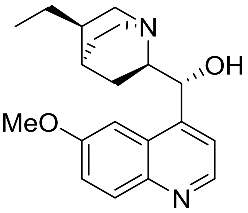

Compound **S5** was prepared according to the procedure described in the literature.[8] White solid. ^{1}H NMR (300 MHz, $CDCl_3$) δ 8.60 (d, *J* = 4.5 Hz, 1H), 7.96 (d, *J* = 9.2 Hz, 1H), 7.48 (d, *J* = 4.5 Hz, 1H), 7.31 (dd, *J* = 9.2, 2.6 Hz, 1H), 7.24 (d, *J* = 2.6 Hz, 1H), 5.49 (d, *J* = 4.2 Hz, 1H), 3.90 (s, 3H), 3.47 – 3.34 (m, 1H), 3.16 – 2.98 (m, 2H), 2.67 – 2.55 (m, 1H), 2.41 – 2.29 (m, 1H), 1.79 – 1.65 (m, 3H), 1.53 – 1.34 (m, 3H), 1.24 (dq, *J* = 7.5, 5.6 Hz, 2H), 0.80 (t, *J* = 7.3 Hz, 3H). Spectral data agree with data described in the literature.[8] mp168.1-168.9 ˚C.

9-Amino(9-deoxy)epihydroquinine (S6)

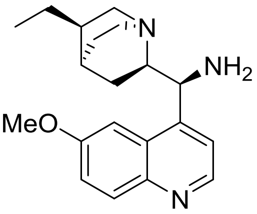

Compound **S6** was prepared according to the procedure described in the literature.[9] ^{1}H NMR (300 MHz, $CDCl_3$) δ 8.74 (d, *J* = 4.5 Hz, 1H), 8.02 (d, *J* = 9.2 Hz, 1H), 7.65 (br s, 1H), 7.46 (d, *J* = 4.4 Hz, 1H), 7.37 (dd, *J* = 9.2, 2.7 Hz, 1H), 4.58 (d, *J* = 10.0 Hz, 1H), 3.96 (s, 3H), 3.24 (dd, *J* = 13.6, 9.7 Hz, 1H), 3.19 – 3.13 (m, 1H), 3.05 (dd, *J* = 17.4, 8.9 Hz, 1H), 2.83 – 2.71 (m, 1H), 2.51 (ddd, *J* = 13.6, 4.4, 2.4 Hz, 1H), 2.09 (br s, 3H), 1.61 – 1.23 (m, 7H), 0.81 (t, *J* = 7.3 Hz, 3H), 0.73 (dd, *J* = 13.6, 7.6 Hz, 1H). Spectral data agree with data described in the literature.[9]

---

[8] Vakulya, B.; Varga, Sz.; Csámpai, A.; Soós, T. *Org. Lett.* **2005**, *7*, 1967-1969.

[9] Melchiorre, P.; Bravo Lara, F.; Martin, R. Patent: EP2687527 A1, 2014.

Catalyst C1

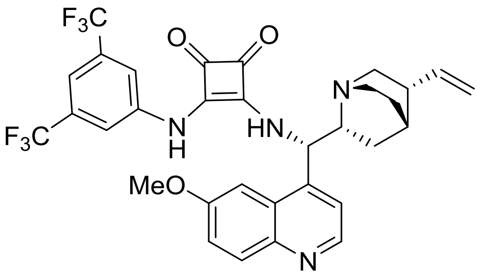

Catalyst **C1** was prepared according to the procedure described in the literature.[5] White solid. ^{1}H NMR (600 MHz, DMSO-d_6) δ 10.14 (br s, 1H), 8.80 (d, *J* = 4.0 Hz, 1H), 8.28 (br s, 1H), 7.98 – 7.92 (m, 3H), 7.73 (s, 1H), 7.65 (d, *J* = 4.5 Hz, 1H), 7.62 (s, 1H), 7.43 (dd, *J* = 9.1, 1.9 Hz, 1H), 6.09 – 5.92 (m, 2H), 5.02 (s, 1H), 4.97 (d, *J* = 10.3 Hz, 1H), 3.93 (s, 3H), 3.50 – 3.41 (m, 1H), 3.37 – 3.23 (m, 1H), 3.18 (dd, *J* = 13.0, 10.5 Hz, 1H), 2.73 – 2.59 (m, 2H), 2.27 (br s, 1H), 1.58 (s, 1H), 1.50 (d, *J* = 9.2 Hz, 3H), 0.69 – 0.59 (m, 1H). ^{13}C NMR (151 MHz, DMSO-d_6) δ 185.17, 180.50, 168.97, 163.13, 158.30, 148.20, 144.72, 143.43, 142.66, 141.25, 131.65 (q, *J* = 33.2 Hz), 123.55 (q, *J* = 273.2 Hz) 122.34, 118.76, 115.36, 114.78, 101.90, 59.36, 56.12, 53.74, 40.56, 27.73, 25.92. Spectral data agree with data described in the literature.[5] mp 226 °C (decomp). IR $\bar{\upsilon}_{max}$ (cm^{-1}): υ 3191 (m, N-H), 2940 (m, N-H), 1795 (m, C=O), 1669 (m, C=C), 1571 (s, C=C), 1445 (s, C-F), 1274 (s, C-O), 1118 (s, C-O). $[\alpha]_D^{25}$ = -52.6 (*c* 0.5, DMSO).

Catalyst C2

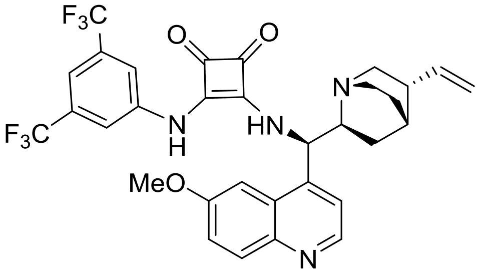

Catalyst **C2** was prepared according to the procedure described in the literature.[5] Pale yellow solid. ^{1}H NMR (300 MHz, $CDCl_3$) δ 8.65 (d, *J* = 4.5 Hz, 1H), 7.96 (d, *J* = 9.1 Hz, 1H), 7.65-7.82 (m, 3H), 7.43 – 7.31 (m, 3H), 6.20 (br s, 1H), 6.05 (br s, 1H), 5.87 – 5.81 (m, 1H), 5.24 (d, *J* = 17.3 Hz, 1H), 5.15 (d, *J* = 10.6 Hz, 1H), 4.00 (s, 3H), 3.20-3.34 (m, 1H),3.06 (br s, 1H), 2.98 – 2.89 (m, 1H), 2.88 – 2.80 (m, 2H), 1.71 (s, 1H), 1.62 (m, 2H), 1.15-1.02 (m, 2H). ^{13}C NMR (75 MHz, $CDCl_3$) δ 184.43, 181.12, 173.19, 169.12, 165.38, 163.09, 158.78, 147.35, 144.86, 140.33, 139.67, 132.69 (q, *J* = 33.7 Hz), 131.79, 129.72, 127.59, 122.92, 118.90, 117.94, 115.95, 115.36, 100.87, 63.01, 55.91, 53.41, 49.29, 46.44, 38.61, 27.27, 26.31, 25.32. Spectral data agree with data described in the literature.[5] IR $\bar{\upsilon}_{max}$ (cm^{-1}): υ 3187 (m, N-H), 3101 (m, N-H), 3031 (m, Ar C-H), 2974 (m, Ar C-H), 1793 (m, C=O), 1715 (m, C=O), 1578 (s, C=C), 1474 (m, C-F), 1442 (s, C-F), 1404 (m, C-F), 1270 (s, C-O), 1125 (s, C-O). $[\alpha]_D^{25}$ = + 56.8 (*c* 0.5, DMSO), mp 169.7-171.0 °C (decomp).

Catalyst C3

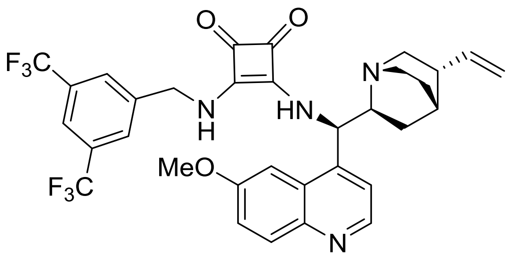

Catalyst **C3** was prepared according to the procedure described in the literature. [10] White solid. ^{1}H NMR (300 MHz, $CDCl_3$) δ 8.67 (d, *J* = 4.5 Hz, 1H), 7.95 (d, *J* = 9.2 Hz, 1H), 7.76 (s, 2H), 7.52 (d, *J* = 16.8 Hz, 1H), 7.37 (dd, *J* = 8.9, 6.2 Hz, 1H), 5.94 – 5.81 (m, 1H), 5.49 (br s, 1H) 5.26 – 5.10 (m, 1H), 4.58 (s, 2H), 3.94 (s, 3H), 3.09 – 2.89 (m, 3H), 2.37 (d, *J* = 6.7 Hz, 1H), 1.59 (dd, *J* = 17.7, 9.6 Hz, 3H), 1.32 – 1.19 (m, 3H), 0.89 (dd, *J* = 15.6, 7.3 Hz, 1H). ^{13}C NMR (151 MHz, $CDCl_3$ + DMSO-d_6) δ 183.24, 182.50, 172.41, 158.23, 147.57, 144.66, 142.21, 140.71, 131.48 (t, *J* = 16.6 Hz), 131.14, 130.93, 128.71, 127.92, 124.28, 122.47, 121.21, 121.18, 121.16, 114.71, 101.42, 59.70, 55.84, 54.47, 49.48, 46.38, 46.26, 39.06, 27.65, 26.67, 25.58. 22.72. Spectral data agree with data described in the literature.[10, 11] $[\alpha]_D^{30}$ = + 97.8 (*c* 0.1, $CHCl_3$), mp 124-128 ˚C (decomp.).

Catalyst C4

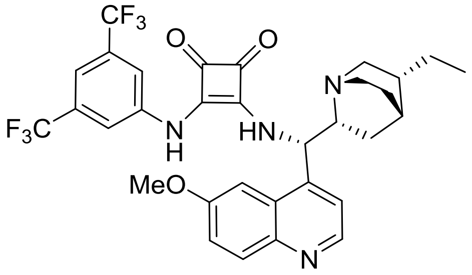

Catalyst **C4** was prepared according to the procedure described in the literature.[12] White solid. ^{1}H NMR (600 MHz, DMSO-d_6) δ 10.31 (br s, 1H), 8.80 (d, *J* = 3.9 Hz, 1H), 8.61 (br s, 1H), 7.97 (d, *J* = 9.8 Hz, 2H), 7.70 (s, 1H), 7.68 (d, *J* = 3.4 Hz, 1H), 7.62 (s, 1H), 7.44 (dd, *J* = 9.3, 2.0 Hz, 1H), 6.07 (br s, 1H), 3.93 (s, 3H), 3.33 (br s, 4H), 2.48 (s, 2H), 1.48 (dd, *J* = 80.1, 73.6 Hz, 6H), 0.80 (t, *J* = 7.2 Hz, 3H), 0.69 (d, *J* = 12.3 Hz, 1H). ^{13}C NMR (151 MHz, DMSO-d_6) δ 188.32, 185.14, 180.42, 171.86, 170.75, 168.98, 158.39, 148.22, 144.73, 141.34, 131.70 (dd, *J* = 64.6, 31.5 Hz), 129.22, 128.59, 126.50, 126.25, 124.44, 122.63, 122.40, 120.82, 118.56, 115.35, 101.75, 60.18, 59.39, 56.13, 40.80, 22.94, 21.18, 14.51, 12.27. Spectral data agree with data described in the literature.[12] $[\alpha]_D^{20}$ = - 53.1 (*c* 1.0, DMSO), mp 168-175 ˚C (decomp.).

[10] Rao, K. S.; Ramesh, P.; Trivedi, R.; Kantam, M. L. *Tetrahedron Lett.* **2016**, *57*, 1227-1231.
[11] George, J.; Sridhar, B.; Subba Reddy, B. V. *Org. Biomol. Chem.* **2014**, *12*, 1595-1602.
[12] Bae, H. Y.; Some, S.; Lee, J. H.; Kim, J.-Y.; Song, M. J.; Lee, S.; Zhang, Y. J.; Song, C. E. *Adv. Synth. Catal.* **2011** , *353*, 3196 – 3202.

Synthesis of the cyclohexanediamine-derived catalysts

N-((1S,2S)-2-Aminocyclohexyl)acetamide ((S,S)-S7)

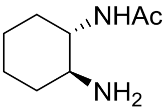

To a solution of ethyl acetimidate hydrochloride (2.40 g, 19.5 mmol) in anhydrous EtOH (24 mL) was added (*S*,*S*)-1,2-diaminocyclohexane (1.23 g, 10.7 mmol). The reaction mixture was stirred for 12 h at room temperature. After evaporation of the solvent, 10 M NaOH (10 mL) was added and the mixture was extracted with CH_2Cl_2 (5 × 30 mL). The combined organic extracts were concentrated and a mixture of EtOH–H_2O (1:2, v/v) was added to the residue. The mixture was refluxed for 12 h. After this time, the solvent was evaporated. The product was used in the next step without further purification (2,04 g, 67%). Brown solid. ^{1}H NMR (300 MHz, $CDCl_3$) δ 5.48 (s, 1H), 3.59 – 3.44 (m, 1H), 2.36 (td, *J* = 10.4, 3.9 Hz, 1H), 2.01 (s, 3H), 1.99 – 1.93 (m, 1H), 1.79 – 1.61 (m, 7H), 1.39 – 1.07 (m, 4H). ^{13}C NMR (75 MHz, $CDCl_3$) δ 170.38, 56.01, 55.60, 35.62, 32.63, 25.08, 25.03, 23.68. Spectral data agree with data described in the literature.[13] IR $\bar{\upsilon}_{max}$ (cm^{-1}): υ 3274 (s, N-H), 2923 (m, N-H), 2855 (m, N-H), 1633 (s, C=O). $[\alpha]_D^{25}$ = - 14.0 (*c* 0.1, $CHCl_3$).

N-((1S,2S)-2-(Piperidin-1-yl)cyclohexyl)acetamide ((S,S)-S8)

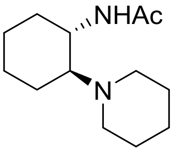

To a solution of the amide (*S*,*S*)-**S7** (1.49 g, 9.54 mmol) in acetonitrile (12 mL) were sequentially added 1,5-dibromopentane (2.19 g, 9.54 mmol, 1.3 ml) and DIPEA (2.56 g, 19.8 mmol, 3,45 ml). The mixture was irradiated in a microwave reactor for 3 h at 110 °C. After cooling, a solution of KOH (1.12 g, 19.9 mmol) in MeOH (8 mL) was added and the reaction mixture was concentrated. The residue was diluted with $CHCl_3$ (150 mL) and the precipitate was removed by filtration. The dried (Na_2SO_4) filtrate was concentrated in vacuo to afford *N*-((1*S*,2*S*)-2-piperidin-1-yl)-cyclohexyl)acetamide (*S*,*S*)-S8 (2.03 g, 95%). Colorless solid. ^{1}H NMR (300 MHz, $CDCl_3$) δ 6.24 (br s, 1H), 3.43 (tt, *J* = 10.7, 3.9 Hz, 1H), 2.63 – 2.48 (m, 3H), 2.33 – 2.15 (m, 3H), 1.96 (s, 3H), 1.91 – 1.75 (m, 2H), 1.69 – 0.93 (m, 11H). Spectral data agree with data described in the literature.[13] $[\alpha]_D^{20}$ = + 66.0 (*c* 0.1, $CHCl_3$), mp 123-124 ˚C.

(1S,2S)-2-(Piperidin-1-yl)cyclohexan-1-amine ((S,S)-12)

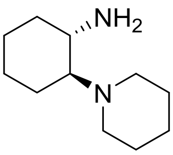

The crude *N*-((1*S*,2*S*)-2-(piperidin-1-yl)cyclohexyl)acetamide (*S*,*S*)-**S8** (2.03 g, 9.06 mmol) was dissolved in HCl (2:1, v/v, 30 mL) and washed with a small amount of toluene (5 ml). The aqueous was heated for 17 h at reflux. After adding 50% NaOH (10 mL), the free amine was extracted with CH_2Cl_2 (3 × 50 mL). The extract was washed with brine, dried (Na_2SO_4) and concentrated in vacuo to afford (*S*,*S*)-2-(piperidin-1-yl)cyclohexanamine (*S*,*S*)-**12** (145 g, 88%). Brown liquid.^{1}H NMR (300 MHz, $CDCl_3$) δ 2.66 – 2.54 (m, 3H), 2.33 – 2.23 (m, 2H), 2.02 – 1.89 (m, 2H), 1.75 (dd, *J* = 9.0, 5.1 Hz, 3H), 1.67 – 1.35 (m, 6H), 1.27 – 0.99 (m, 4H). Spectral data are in agreement with data described

[13] Baran, R.; Veverková, E.; Škvorcová, A.; Šebesta, R. *Org. Biomol. Chem.* **2013**, *11*, 7705-7711.

in the literature.[14] IR $\bar{\upsilon}_{max}$ (cm^{-1}): υ 2924 (s, N-H), 2852 (s, N-H), 2792 (m, N-H), 1448 (s, CH_2). $[\alpha]_D^{20}$ = + 54.2 (*c* 0.1, $CHCl_3$)

(1*R*,2*R*)-Derivatives **S7**, **S8** and **12** were prepared using the same procedures.

Catalyst (*R*,*R*)-C5

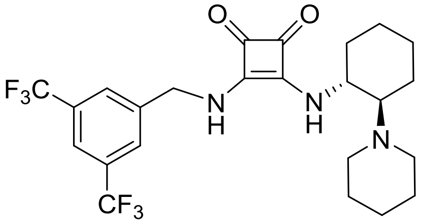

Catalyst (*R*,*R*)-**C5** was prepared according to the procedure described in the literature.[13] White solid. ^{1}H NMR (300 MHz, DMSO-d_6) δ 8.06 (s, 1H), 7.92 (br s, 1H), 7.34 (br s, 1H), 4.92 (s, 2H), 3.81 (br s, 1H), 2.20 (d, *J* = 10.1 Hz, 3H), 1.98 (s, 1H), 1.83 – 1.58 (m, 3H), 1.35 – 1.10 (m, 10H). ^{13}C NMR (75 MHz, DMSO-d_6) δ 182.82, 181.96, 168.96, 166.65, 142.89, 130.39 (q, *J* = 32.8 Hz), 128.64, 128.34, 125.03, 121.41, 121.06, 121.01, 117.79, 68.36, 53.92, 49.27, 45.55, 33.88, 26.15, 24.79, 24.46, 24.36, 23.51. Spectral data agree with data described in the literature.[13] $[\alpha]^D_{20}$ = -4.81 (c = 0.31, DMSO), mp 238 °C (decomp.).

Catalyst (*S*,*S*)-C5

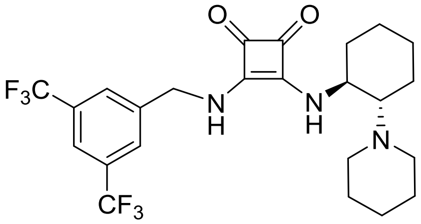

(1*S*,2*S*)-2-(Piperidin-1-yl)cyclohexan-1-amine ((*S*,*S*)-**12**, 52 mg, 0.283 mmol) and 3-((3,5-bis(trifluoro-methyl)benzyl)amino)-4-methoxycyclobut-3-ene-1,2-dione (**S2**, 100 mg, 0.283 mmol) was dissolved in DCM (1 mL) and was stirred at r.t. for 48 h. The precipitated catalyst was washed with ice-cold DCM and dried (84 mg, 59%). White solid. ^{1}H NMR (300 MHz, DMSO-d_6) δ 8.06 (s, 3H), 7.86 (br s, 1H), 7.30 (br s, 1H), 4.91 (s, 2H), 3.80 (br s, 1H), 2.20 (d, *J* = 10.4 Hz, 3H), 2.00 (d, *J* = 9.1 Hz, 1H), 1.85 – 1.59 (m, 3H), 1.35 – 1.12 (m, 10H). ^{13}C NMR (151 MHz, DMSO-d_6) δ 183.4, 182.5, 169.5, 167.2, 143.4, 130.9 (q, *J* = 32.8 Hz), 128.84, 126.44, 124.63, 122.82, 121.59, 121.57, 121.54, 121.01, 68.89, 54.43, 49.78, 46.1, 34.4, 26.7, 25.3, 25.0, 24.9, 24.0. Spectral data agree with data described in the literature.[13] $[\alpha]^D_{20}$ = -4.11 (c = 1.00, DMSO), mp 238 ˚C (decomp.).

Catalyst C6

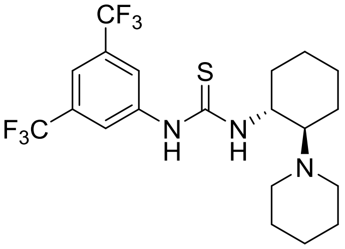

[14] Gonzalez-Sabin, J.; Gotor, V.; Rebolledo, F. *Chem. Eur. J.* **2004**, *10*, 5788-5794.

Catalyst **C6** was prepared according to the procedure described in the literature.[15] White solid. ^{1}H NMR (600 MHz, CD_3OD) δ 8.17 (s, 2H), 7.62 (s, 1H), 4.24 – 4.15 (m, 1H), 2.76 – 2.68 (m, 2H), 2.51 – 2.36 (m,4H), 1.99 – 1.94 (m, 1H), 1.84 – 1.78 (m, 1H), 1.70 (d, J = 12.0 Hz, 1H), 1.61 – 1.39 (m, 7H), 1.37 – 1.15 (m, 5H). ^{13}C NMR (151 MHz, CD_3OD) δ 181.6, 143.1, 133.2, 132.9, 132.7, 132.5, 127.5, 125.7, 123.9, 123.6, 122.1, 117.7, 117.7, 69.0, 56.3, 56.2, 50.5, 33.5, 27.7, 26.6, 26.0, 24.7. Spectral data agree with data described in the literature.[15] IR $\bar{\upsilon}_{max}$ (cm^{-1}): υ 3924 (w, N-H), 1524 (m, C=S), 1473 (m, C-H), 1400 (s, C-H), 1374 (s, C-F), 1136 (s, C-C). $[\alpha]^D_{20}$ = -4.80 (c = 0.53, DMSO), mp 59 ˚C (decomp.).

Catalyst (*R*,*R*)-C7

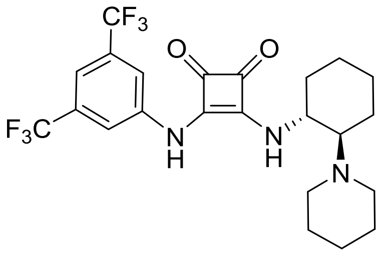

Catalyst (*R*,*R*)-**C7** was prepared according to the procedure described in the literature.[16] White solid. ^{1}H NMR (300 MHz, DMSO-d_6) δ 10.19 (br s, 1H), 8.06 (s, 2H), 7.66 (s, 1H), 3.91 (s, 1H), 2.64 (d, J = 8.2 Hz, 2H), 2.29 (m, 3H), 2.07 (d, J = 11.3 Hz, 1H), 1.84 (s, 1H), 1.78 – 1.61 (m, 1H), 1.27 (d, J = 20.6 Hz, 10H). ^{13}C NMR (75 MHz, DMSO-d_6) δ 184.7, 180.1, 170.2, 161.6, 141.2, 131.3 (q, J = 32.3 Hz), 128.6, 125.0, 121.4, 117.9, 114.5, 68.3, 54.5, 49.4, 33.88, 26.4, 24.7, 24.5, 24.4, 23.4. Spectral data agree with data described in the literature.[16] $[\alpha]^D_{25}$ = +149.7 (c = + 0.62, DCM), mp 137 °C (decomp.).

Synthesis of the binaphtol-derived catalysts

(*S*)-2,2'-Bis(methoxymethoxy)-1,1'-binaphthalene (**S9**)

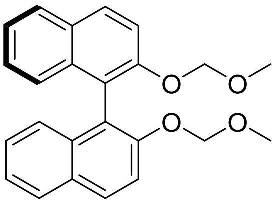

Compound **S9** was prepared according to the procedure described in the literature.[17] White solid. ^{1}H NMR (600 MHz, $CDCl_3$) δ 7.95 (d, J = 9.0 Hz, 2H), 7.87 (d, J = 8.2 Hz, 2H), 7.57 (d, J = 9.0 Hz, 2H), 7.34 (t, J = 7.3 Hz, 2H), 7.22 (t, J = 7.4 Hz, 2H), 7.15 (d, J = 8.5 Hz, 2H), 5.08 (d, J = 6.8 Hz, 2H), 4.97 (d, J = 6.8 Hz, 2H), 3.14 (s, 6H). Spectral data agree with data described in the literature.[17] mp 99.6-100.6 °C.

---

[15] Jing, Z.; Bai, X.; Chen, W.; Zhang, G.; Zhu, B.; Jiang, Z. *Org. Lett.* **2016**, *18*, 260-263.
[16] Du, D-M.; Yang, W. *Adv. Synth. Catal.* **2011**, *353*, 1241 – 1246.
[17] Beckendorf, S.; Mancheno, O. G. *Synthesis* **2012** , *44*, 2162–2172.

(*S*)-2,2'-Bis(methoxymethoxy)-1,1'-binaphthalene-3-carbaldehyde (S10)

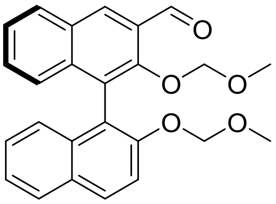

Compound **S10** was prepared according to the procedure described in the literature.[18] Yellow solid. ^{1}H NMR (300 MHz, $CDCl_3$) δ 10.59 (s, *J* = 11.0 Hz, 1H), 8.57 (s, 1H), 8.08 – 7.85 (m, 3H), 7.63 – 7.11 (m, 7H), 5.15 (d, *J* = 6.9 Hz, 1H), 5.04 (d, *J* = 7.0 Hz, 1H), 4.75 (d, *J* = 5.9 Hz, 1H), 4.63 (d, *J* = 5.9 Hz, 1H), 3.16 (s, 3H), 3.00 (s, 3H). Spectral data agree with data described in the literature.[18] IR $\bar{\upsilon}_{max}$ (cm^{-1}): υ 2924 (w, C-H), 2848 (w, C-H), 1688 (C=O).

(*S*)-3-Hydroxymethyl-2,2´-bis(methoxymethyloxy)-1,1´ binaphthalene (S11)

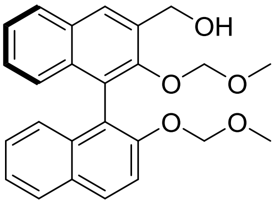

Compound **S11** was prepared according to the procedure described in the literature.[19] Colorless viscous oil. ^{1}H NMR (600 MHz, $CDCl_3$) δ 7.99 (d, *J* = 8.4 Hz, 2H), 7.90 (dd, *J* = 14.0, 6.5 Hz, 2H), 7.60 (d, *J* = 9.1 Hz, 1H), 7.42 – 7.35 (m, 2H), 7.30 – 7.21 (m, 2H), 7.18 – 7.13 (m, 2H), 5.12 (d, *J* = 7.0 Hz, 1H), 5.04 (d, *J* = 7.0 Hz, 1H), 4.92 (qd, *J* = 12.5, 6.6 Hz, 2H), 4.68 (d, *J* = 6.1 Hz, 1H), 4.47 (d, *J* = 6.0 Hz, 1H), 3.25 (s, 3H), 3.15 (s, 3H). Spectral data agree with data described in the literature.[19] IR $\bar{\upsilon}_{max}$ (cm^{-1}): υ 3400 (w, O-H), 2917 (w, C_{Ar}-H), 1197 (m, C-O), 1147 (s, C-O).

(*S*)-3-(Azidomethyl)-2,2'-bis(methoxymethoxy)-1,1'-binaphthalene (S12)

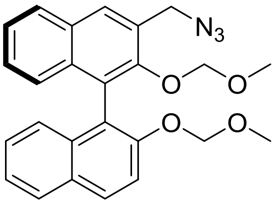

Compound **S12** was prepared according to the procedure described in the literature.[20] Pale yellow solid. ^{1}H NMR (300 MHz, $CDCl_3$) δ 7.93 (dt, *J* = 17.8, 6.5 Hz, 4H), 7.59 (d, *J* = 9.1 Hz, 1H), 7.46 – 7.32 (m, 2H), 7.30 – 7.12 (m, 4H), 5.12 (d, *J* = 6.9 Hz, 1H), 5.04 (d, *J* = 7.0 Hz, 1H), 4.73 (s, 2H), 4.62 (d, *J* = 5.6 Hz, 1H), 4.50 (d, *J* = 5.6 Hz, 1H), 3.17 (s, *J* = 5.0 Hz, 3H), 3,04 (s, 3H). Spectral data agree with data described in the literature.[20]

[18] Hodačová, J.; Stibor, I. *Collect. Czech. Chem. Commun.* **2000**, *65,* 83-98.
[19] Zhang, A.-L., Yu, Z.-d., Yang, L.-W. , Yang, N.-F. *Tetrahedron: Asymmentry* **2015**, *26*, 173-179.
[20] Rahaman, H.; Madarász, Á.; Pápai, I.; Pihko, P. M. *Angew. Chem. Int. Ed.* **2012**, *34*, 8495-8499.

(*S*)-2,2'-bis(methoxymethoxy)-1,1'-binaphthalen-3-ylmethanamine (9)

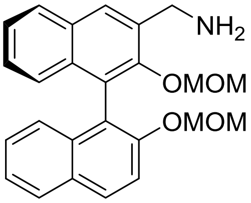

Compound **9** was prepared according to the procedure described in the literature.[20] Viscous brown oil. ^{1}H NMR (300 MHz, $CDCl_3$) δ 7.99 – 7.84 (m, 4H), 7.58 (d, *J* = 9.1 Hz, 1H), 7.37 (dtd, *J* = 8.0, 6.5, 1.4 Hz, 2H), 7.30 – 7.13 (m, 4H), 5.11 (d, *J* = 6.9 Hz, 1H), 5.04 (d, *J* = 6.9 Hz, 1H), 4.64 (d, *J* = 5.8 Hz, 1H), 4.48 (d, *J* = 5.8 Hz, 1H), 4.16 (s, 2H), 3.17 (s, 3H), 3.10 (s, 3H).

(*S*)-3-(((2,2'-dihydroxy-1,1'-binaphthalen-3-yl)methyl)amino)-4-methoxycyclobut-3-ene-1,2-dione (11)

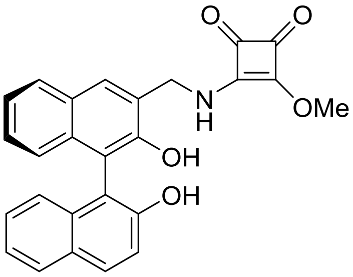

Dimethyl squarate (184 mg, 1.30 mmol) and (2,2'-bis(methoxymethoxy)-1,1'-binaphthalen-3-yl)methanamine (**9**, 350 mg, 0.87 mmol) was dissolved in MeOH (3 mL) and heated using microwave irradiation at 120 °C for 3 h. The reaction mixture was then evaporated and the crude product was purified by column chromatography (CH_2Cl_2→ CH_2Cl_2/MeOH, 90:10). The product **11** was obtained as a yellow solid (300 mg, 80 %). ^{1}H NMR (600 MHz, CD_3OD) δ: 7.91 (d, *J* = 8.9 Hz, 1H), 7.84 (d, *J* = 7.9 Hz, 3H), 7.32 (d, *J* = 8.9 Hz, 1H), 7.27 – 7.24 (m, 2H), 7.16 (t, *J* = 7.2 Hz, 2H), 7.00 (d, *J* = 8.4 Hz, 1H), 6.95 (d, *J* = 8.4 Hz, 1H), 5.05 (s, 1H), 4.84 (br s, 1H), 4.34 (d, *J* = 18.1 Hz, 3H). ^{13}C NMR (151 MHz, CD_3OD) δ: 188.5, 183.6, 176.9, 173.3, 153.6, 151.0, 134.5, 134.0, 130.0, 129.1, 128.7, 128.3, 128.0, 127.8, 127.6, 126.1, 125.9, 124.3, 124.0, 122.9, 122.7, 118.0, 115.2, 112.9, 59.7, 44.9. HRMS (*m/z*): $[M+H]^+$ calcd for $C_{26}H_{19}NO_5$, 426.1336; found 426.1336. $[\alpha]^{D}_{20}$ = -12.8 (c = 0.5, DMSO); mp 156˚C (decomp.).

Catalyst (S_a,*S*,*S*)-C8

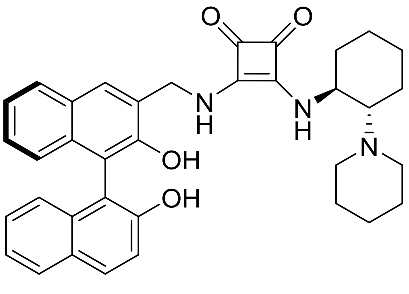

Binol squarate **11** (380 mg, 0.89 mmol) and (1*S*,2*S*)-2-(piperidin-1-yl)cyclohexan-1-amine ((*S*,*S*)-**12,** 194 mg, 1.07 mmol) was dissolved in MeOH (4 mL) and stirred at r.t. for 2 d. The product was purified by flash chromatography (CH_2Cl_2/MeOH, 90:10). The product **C8** was obtained as a yellow solid (350 mg, 68%). ^{1}H NMR (300 MHz, DMSO-d_6) δ: 7.79 (d, *J* = 8.6 Hz, 2H), 7.70 (d, *J* = 6.8 Hz,

2H), 7.23 (d, J = 8.9 Hz, 1H), 7.19 – 6.98 (m, 4H), 6.91 (d, J = 8.4 Hz, 1H), 6.80 (d, J = 8.4 Hz, 1H), 4.92 (s, 2H), 3.79 (s, 1H), 2.71 (s, 1H), 2.64 – 2.51 (m, 2H), 2.33 – 2.13 (m, 2H), 1.81 – 1.54 (m, 7H), 1.32 (dd, J = 21.8, 16.7 Hz, 6H). ^{13}C NMR (151 MHz, DMSO-d_6) δ: 182.8, 182.5, 169.0, 167.8, 156.6, 134.7, 134.3, 132.5, 132.0, 131.9, 129.2, 129.2, 129.0, 128.3, 128.0, 127.1, 125.6, 125.3, 125.2, 122.0, 121.0, 116.8, 68.4, 54.1, 50.3, 49.8, 32.6, 27.0, 26.8, 26.5, 25.4, 24.97, 24.96, 24.5, 24.1, 22.5. HRMS (*m/z*): $[M+H]^+$ calcd for $C_{36}H_{37}N_3O_4$, 576.2818; found 576.2859. mp 130 ˚C (decomp.) $[\alpha]^D{}_{20}$ = + 4.25 (c = 0.5, DMSO)

Catalyst (*S*$_a$,*R*,*R*)-C8

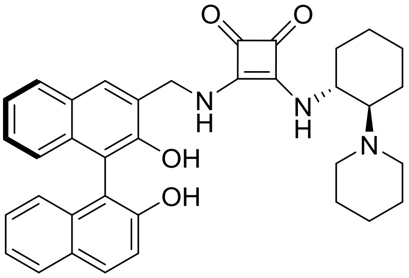

Binol squarate **11** (325 mg, 0.76 mmol) and (1*R*,2*R*)-2-(piperidin-1-yl)cyclohexan-1-amine ((*R*,*R*)-**12,** 167 mg, 0.92 mmol) was dissolved in MeOH (4 mL) and stirred at r.t. for 2 d. The crude product was purified by flash chromatography (CH_2Cl_2/MeOH, 90:10). The product **C8** was obtained as a yellow solid (320 mg, 73%). ^{1}H NMR (600 MHz, DMSO-d_6) δ: 9.39 (br s, 1H), 8.44 (br s, 1H), 7.89 (d, J = 8.9 Hz, 1H), 7.86 (d, J = 8.0 Hz, 1H), 7.83 – 7.76 (m, 2H), 7.33 (d, J = 8.9 Hz, 1H), 7.26 – 7.21 (m, 2H), 7.18 – 7.12 (m, 2H), 6.88 (d, J = 8.4 Hz, 1H), 6.82 (d, J = 8.5 Hz, 1H), 4.96 (d, J = 5.3 Hz, 2H), 3.83 (br s, 1H), 2.62 (s, 2H), 2.25 (d, J = 25.2 Hz, 2H), 2.04 (d, J = 14.4 Hz, 1H), 1.86 – 1.57 (m, 3H), 1.12-1.40 (m, 10H). ^{13}C NMR (151 MHz, DMSO-d_6) δ: 182.8, 182.6, 168.7, 167.9, 154.4, 151.3, 134.8, 133.8, 129.9, 128.9, 128.8, 128.5, 128.5, 128.0, 127.1, 126.5, 126.2, 124.8, 124.5, 123.3, 122.8, 119.2, 116.6, 113.9, 60.2, 55.4, 54.2, 49.8, 49.6, 43.8, 34.9, 26.8, 25.0, 24.0. HRMS (*m/z*): $[M+H]^+$ calcd for $C_{36}H_{37}N_3O_4$, 576.2818; found 576.2859. mp 140 ˚C (decomp.) $[\alpha]^D{}_{20}$ = -3.94 (c = 0.5, DMSO)

Copies of NMR spectra

^{1}H NMR spectrum of **3**

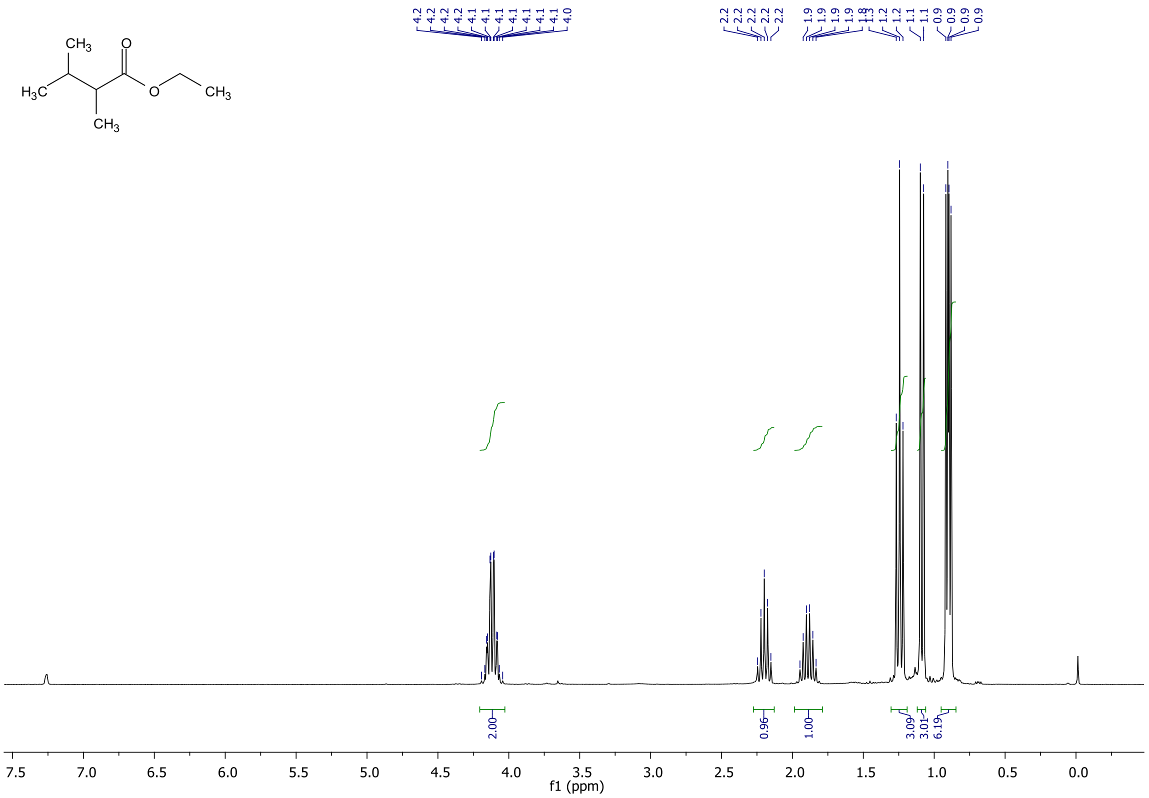

^{1}H NMR spectrum of **4**

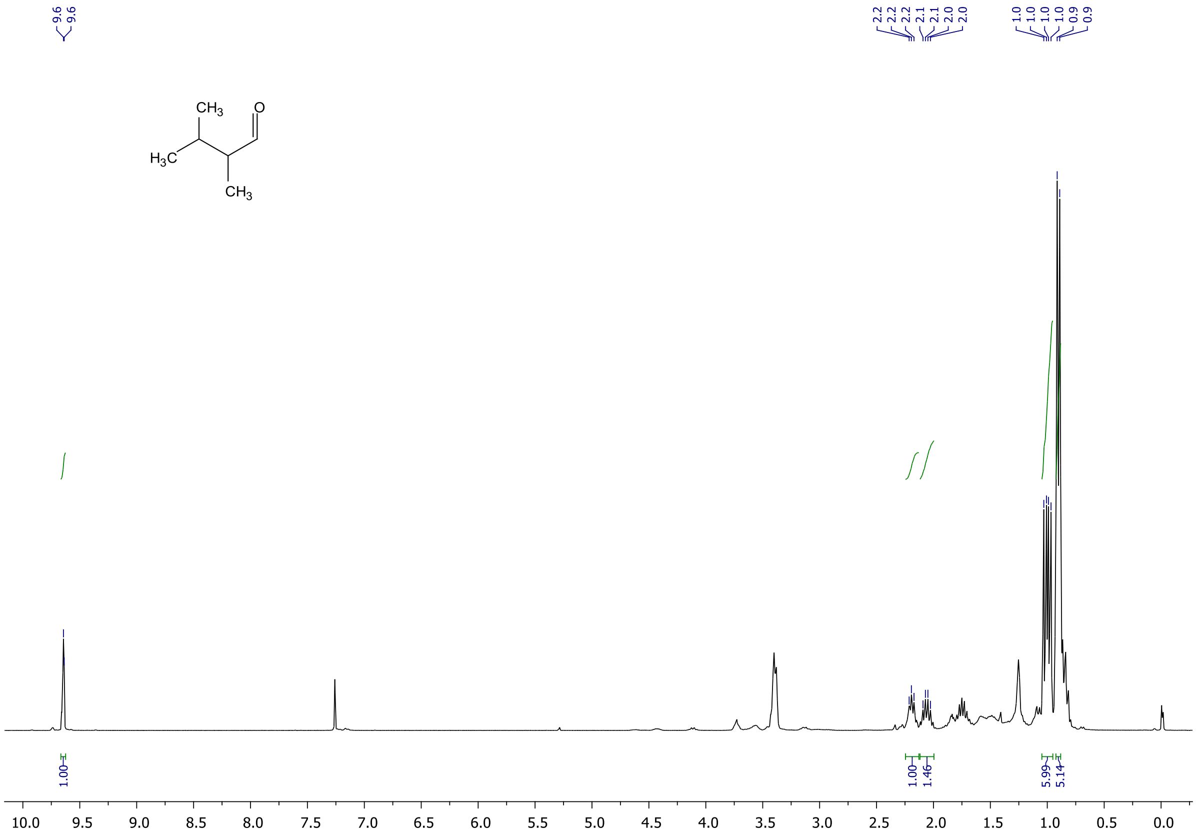

^{1}H NMR spectrum of **5**

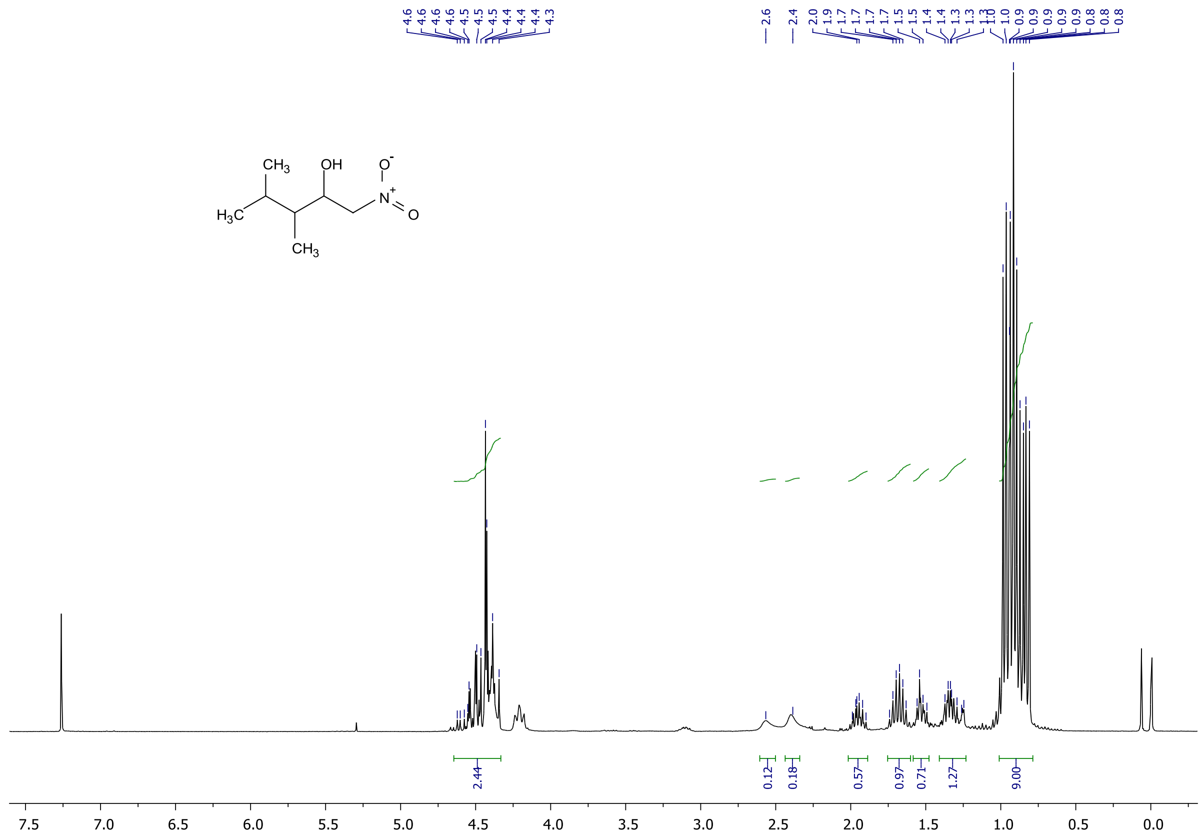

^{1}H NMR spectrum of **6**

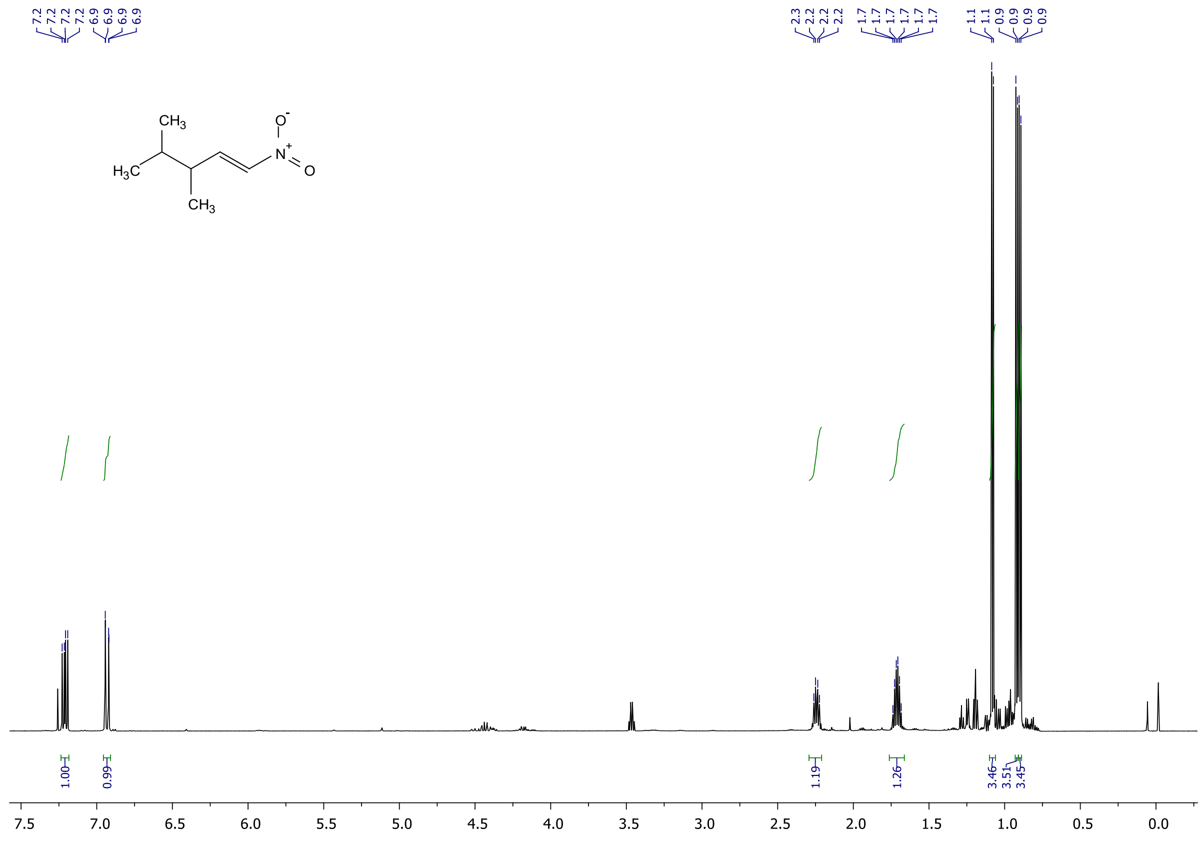

^{13}C NMR spectrum of **6**

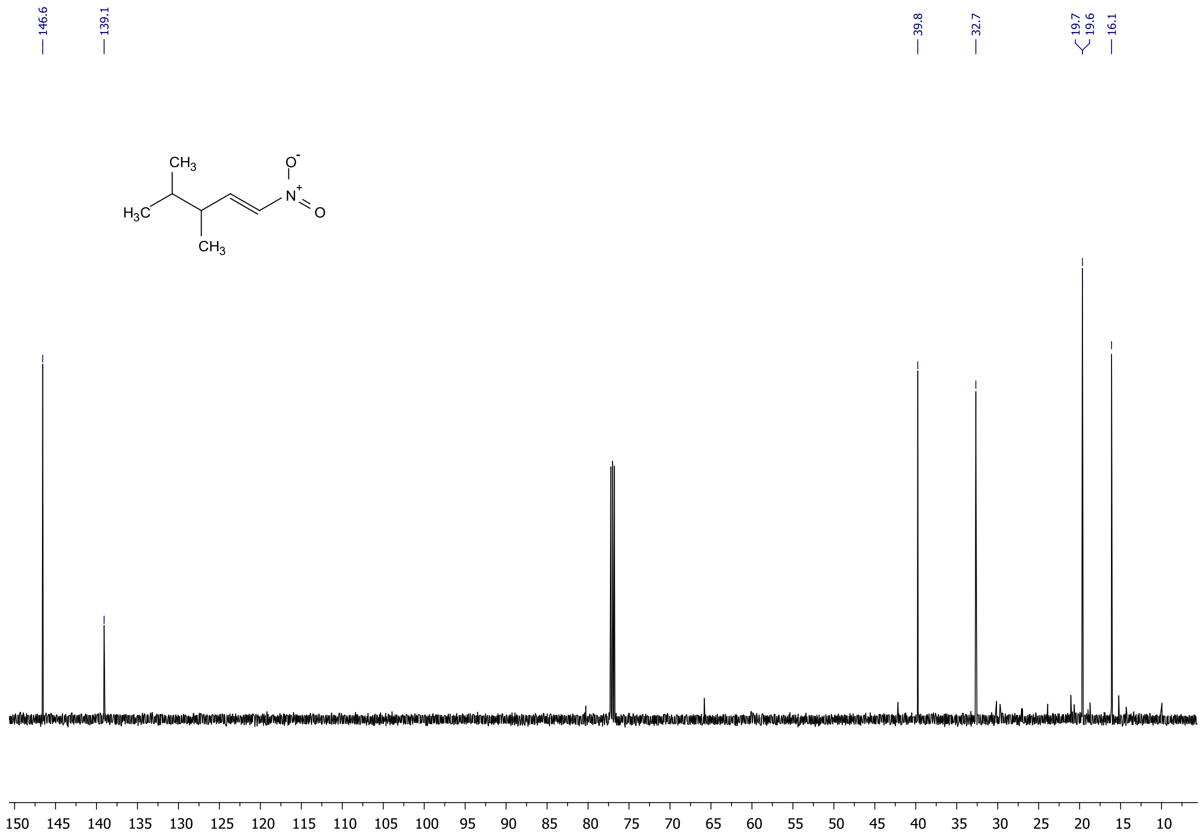

^{1}H NMR spectrum of **7** (major diastereomer)

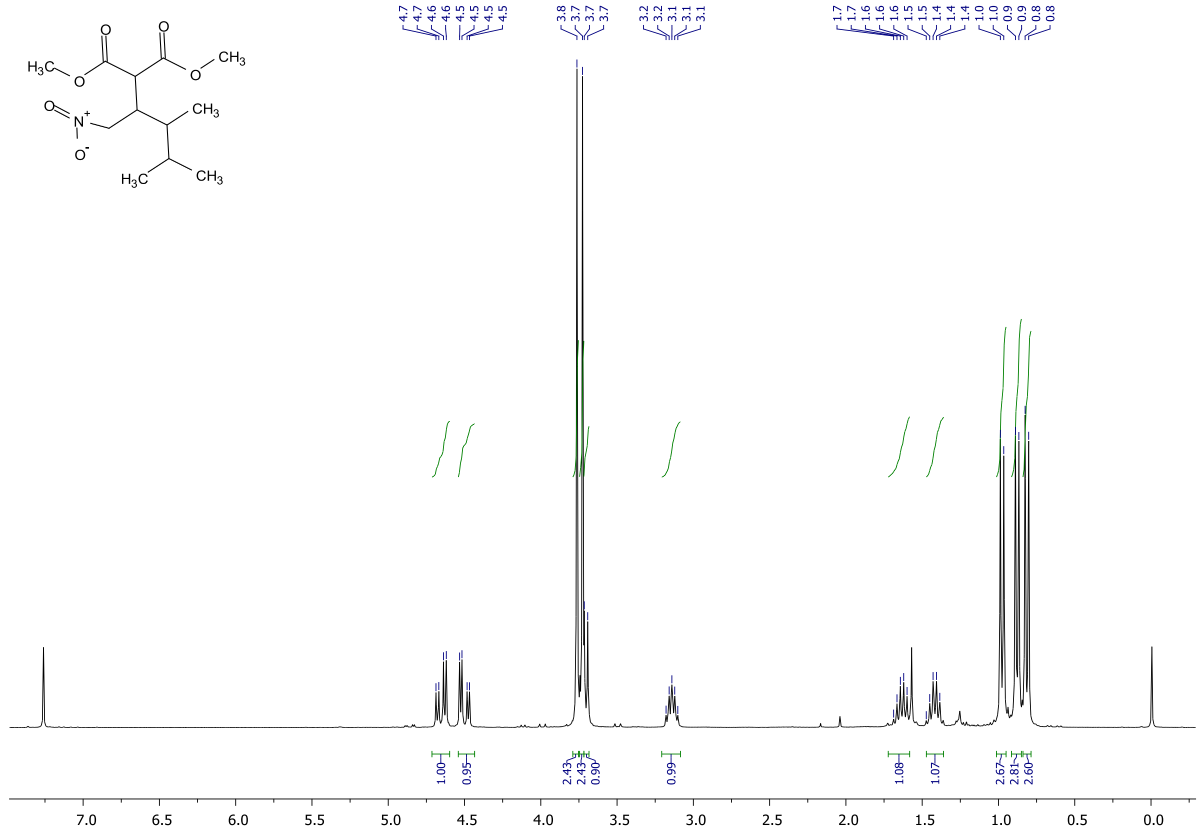

^{13}C NMR spectrum of **7** (major diastereomer)

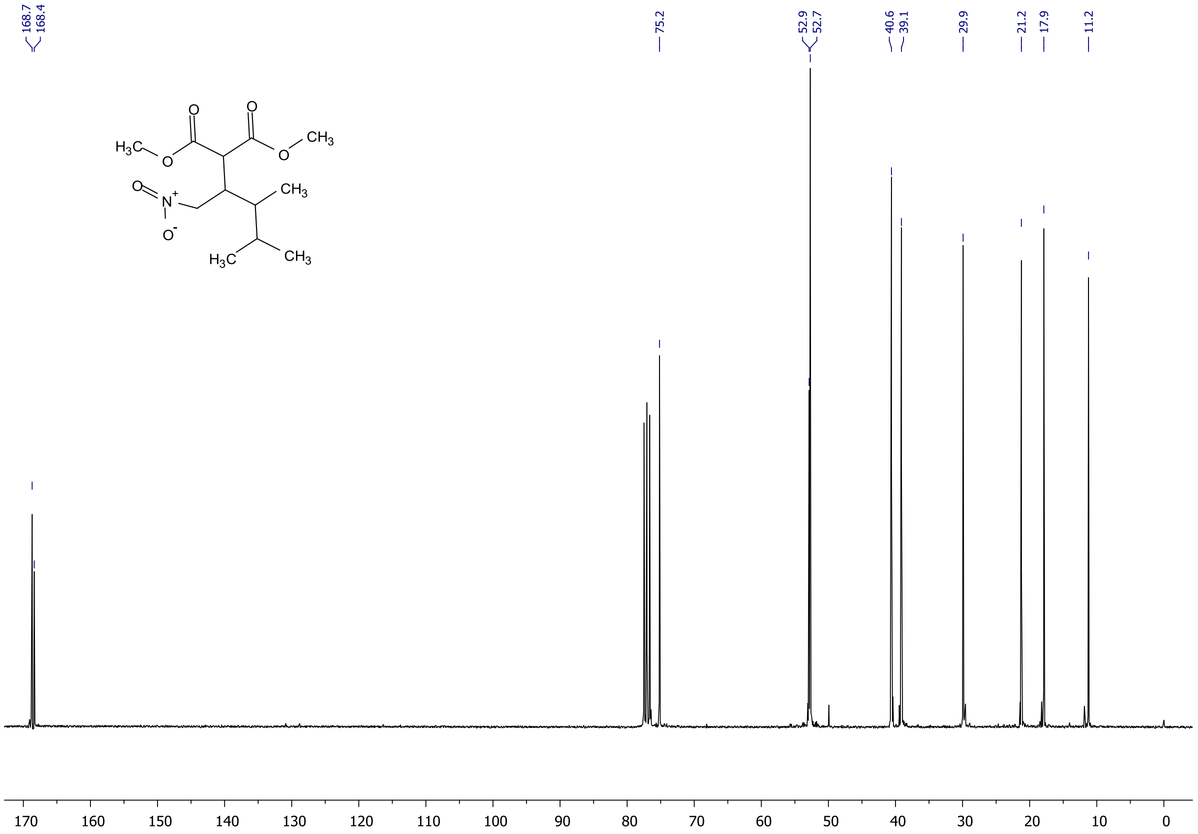

^{1}H-^{1}H COSY NMR spectrum of **7** (major diastereomer)

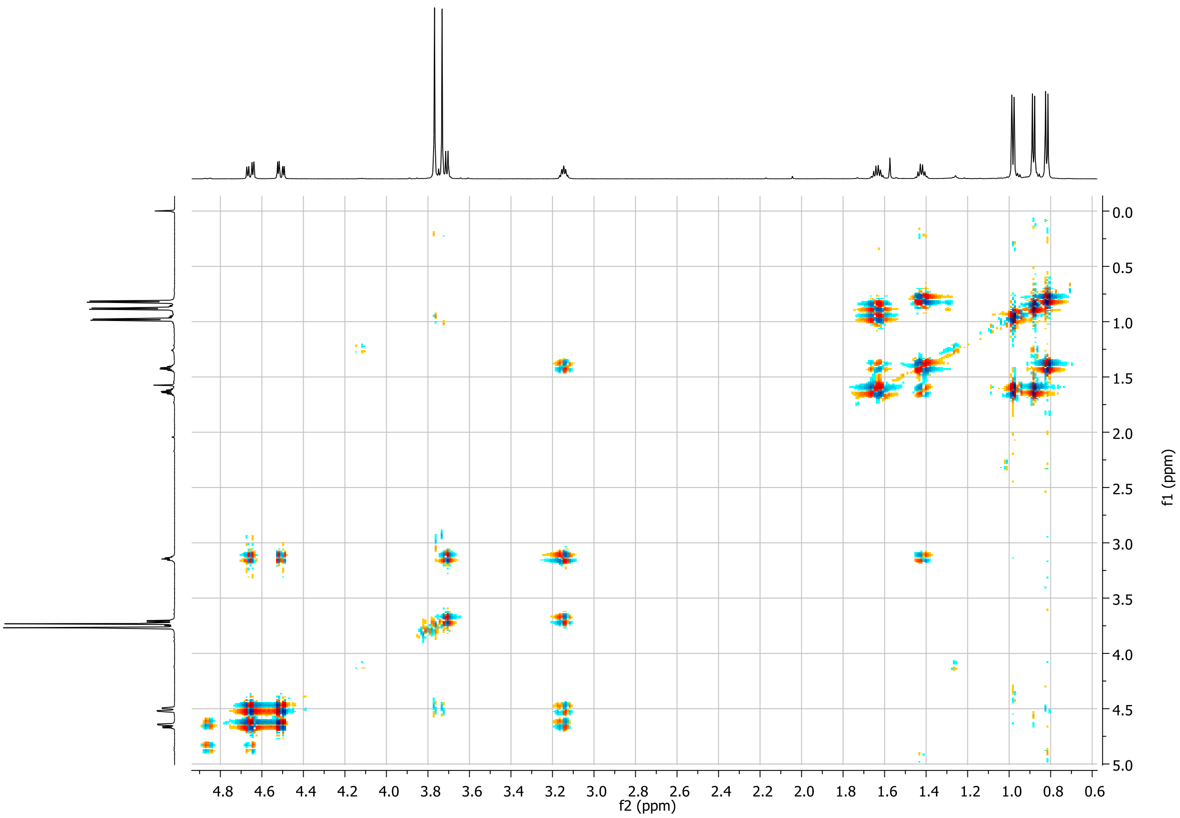

^{1}H NMR spectrum of **7** (minor diastereomer)

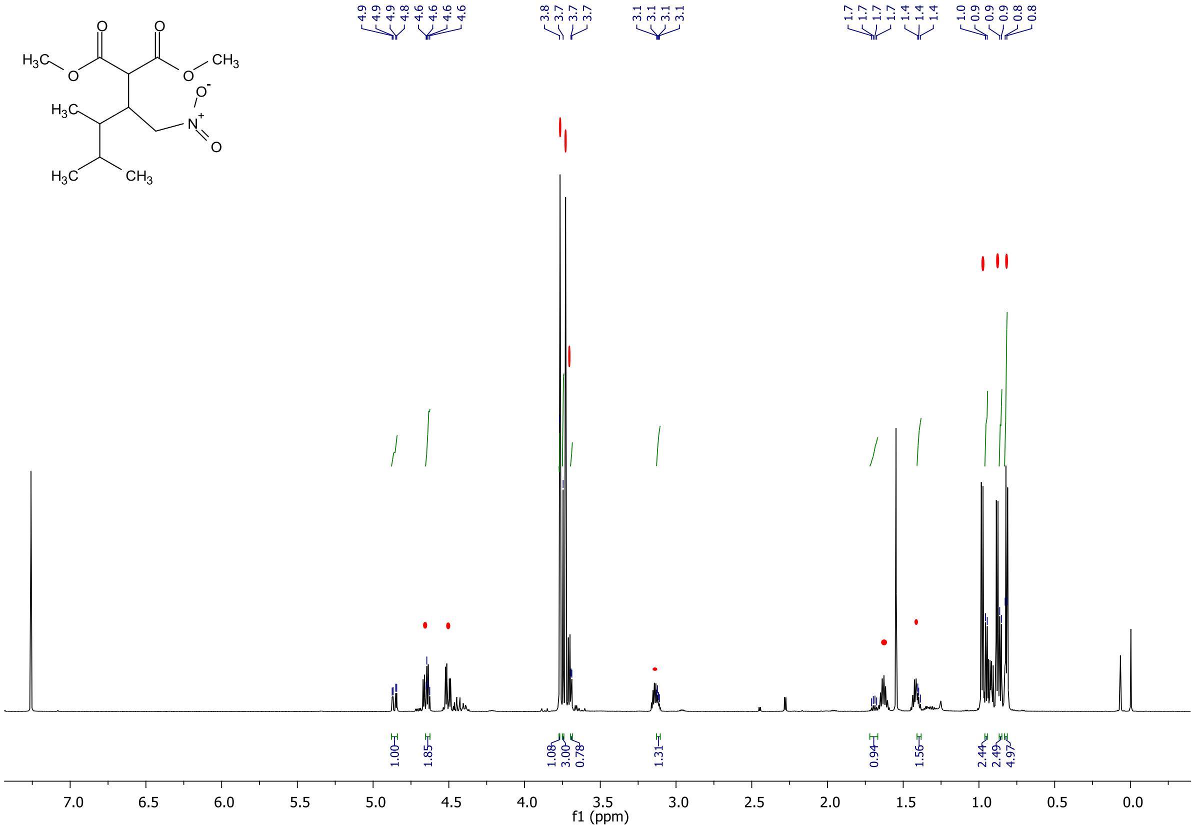

^{13}C NMR spectrum of **7** (minor diastereomer)

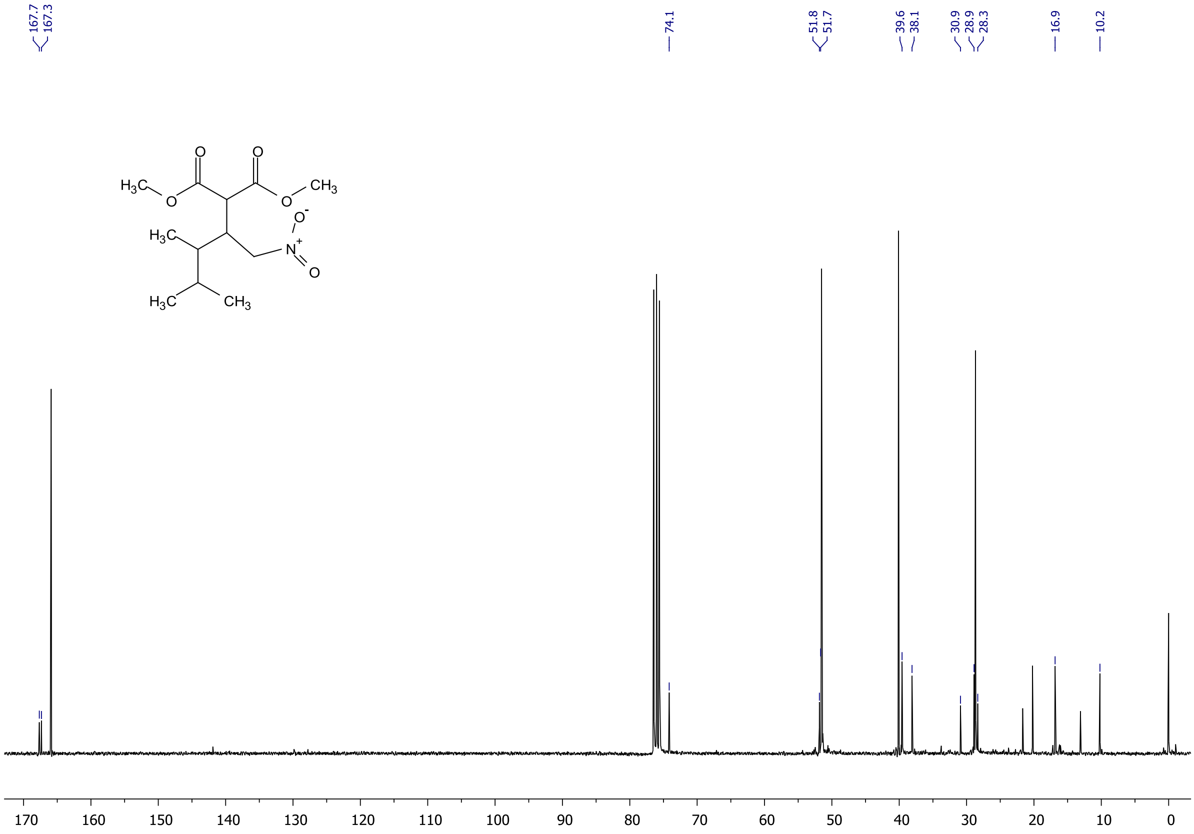

^{1}H NMR spectrum of **1**.HCl

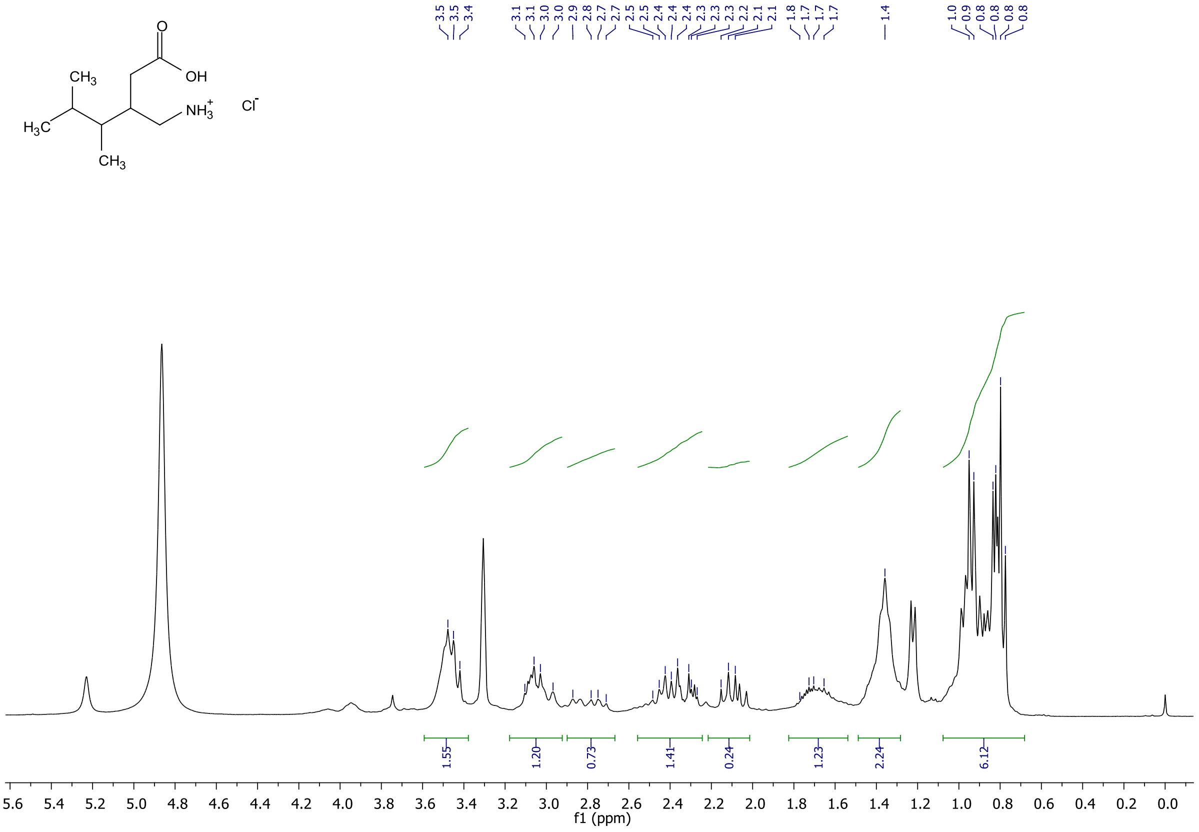

^{1}H NMR spectrum of **10**

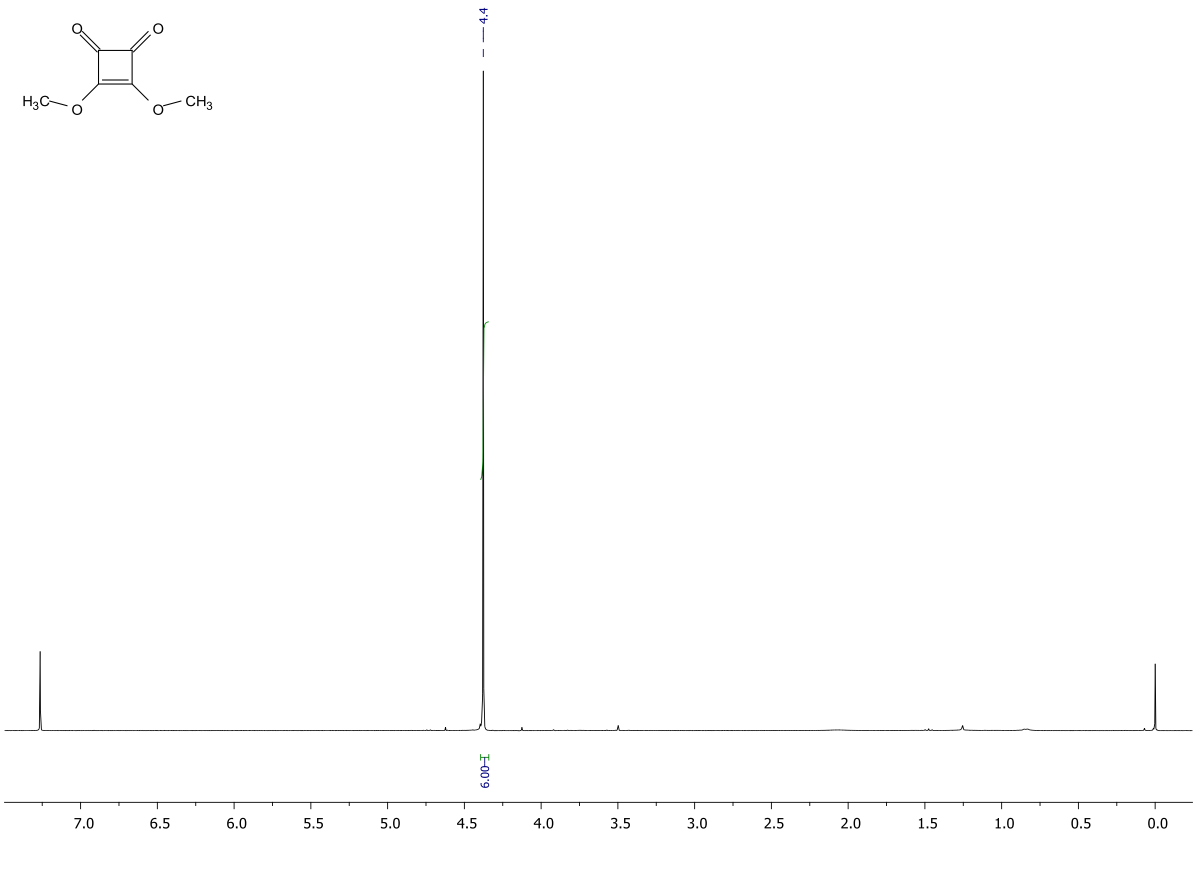

^{1}H NMR spectrum of **S1**

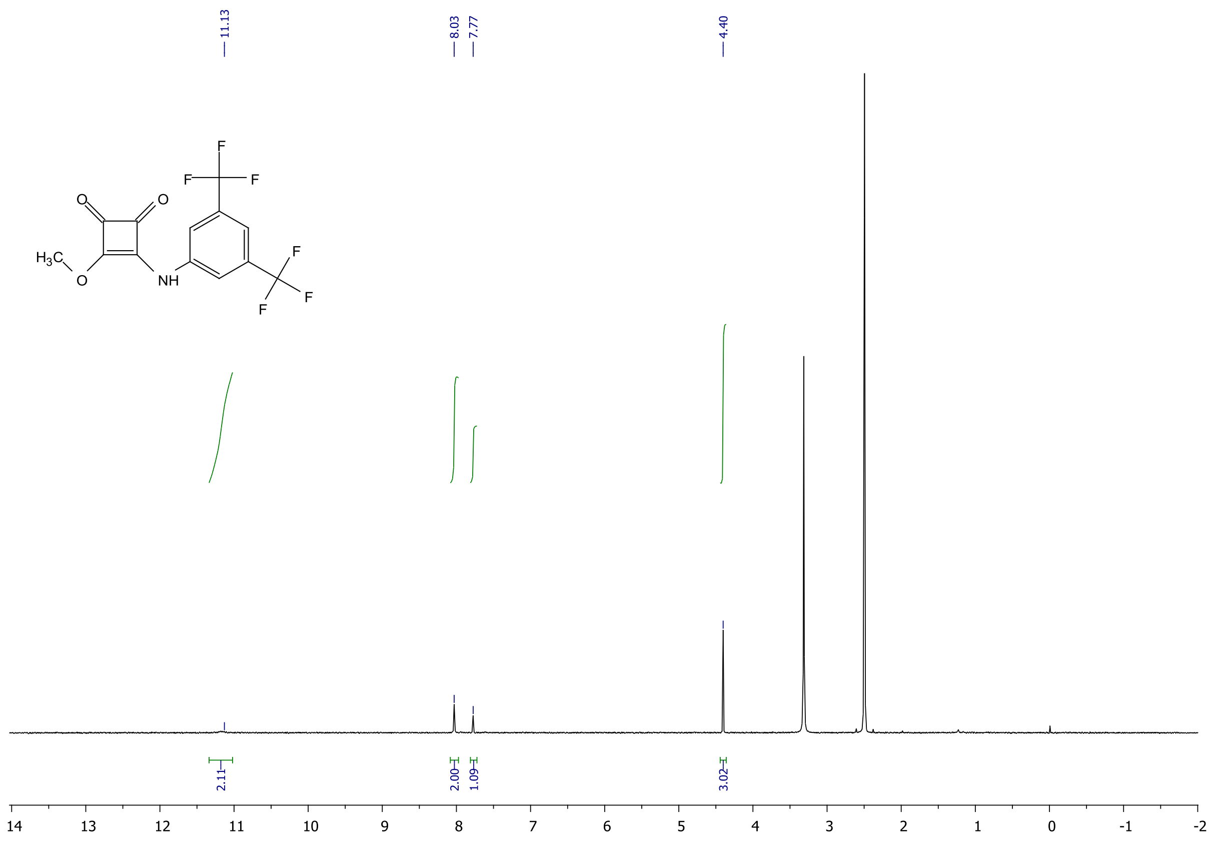

^{1}H NMR spectrum of **S2**

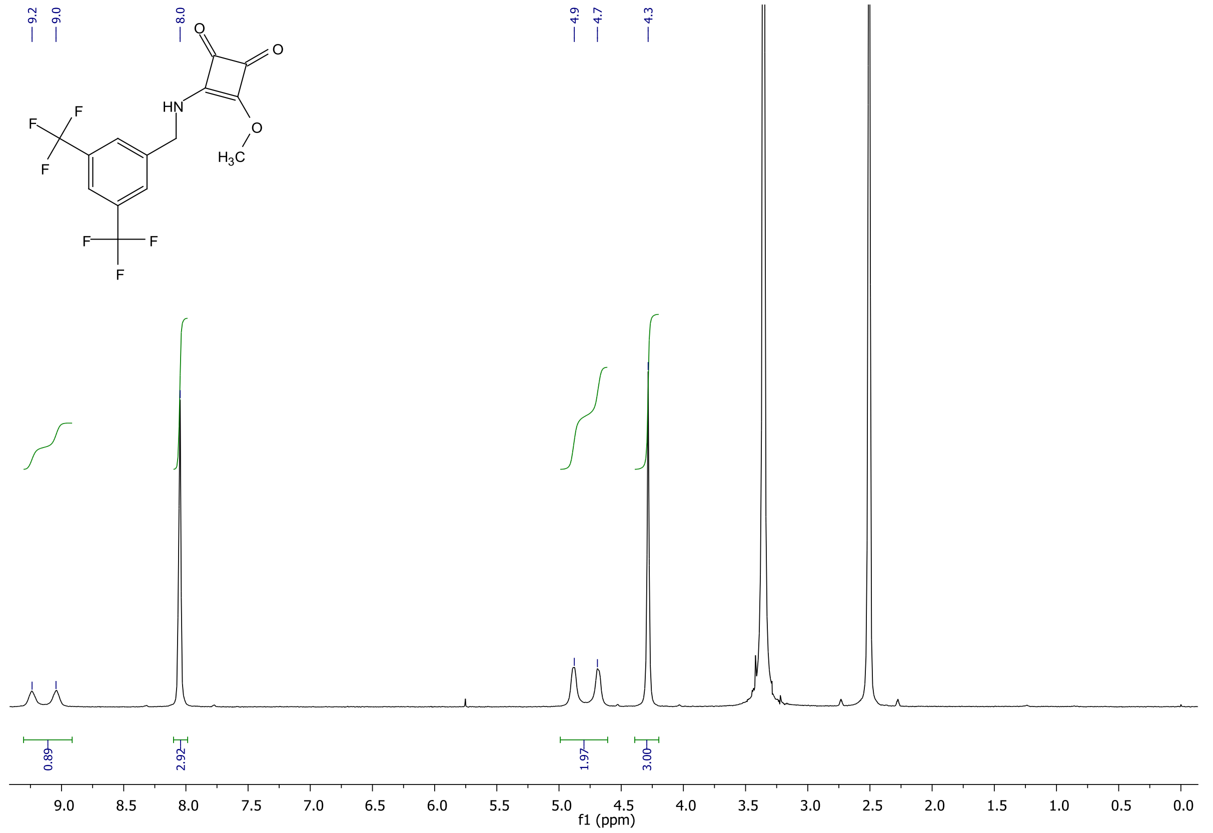

^{1}H NMR spectrum of **S3**

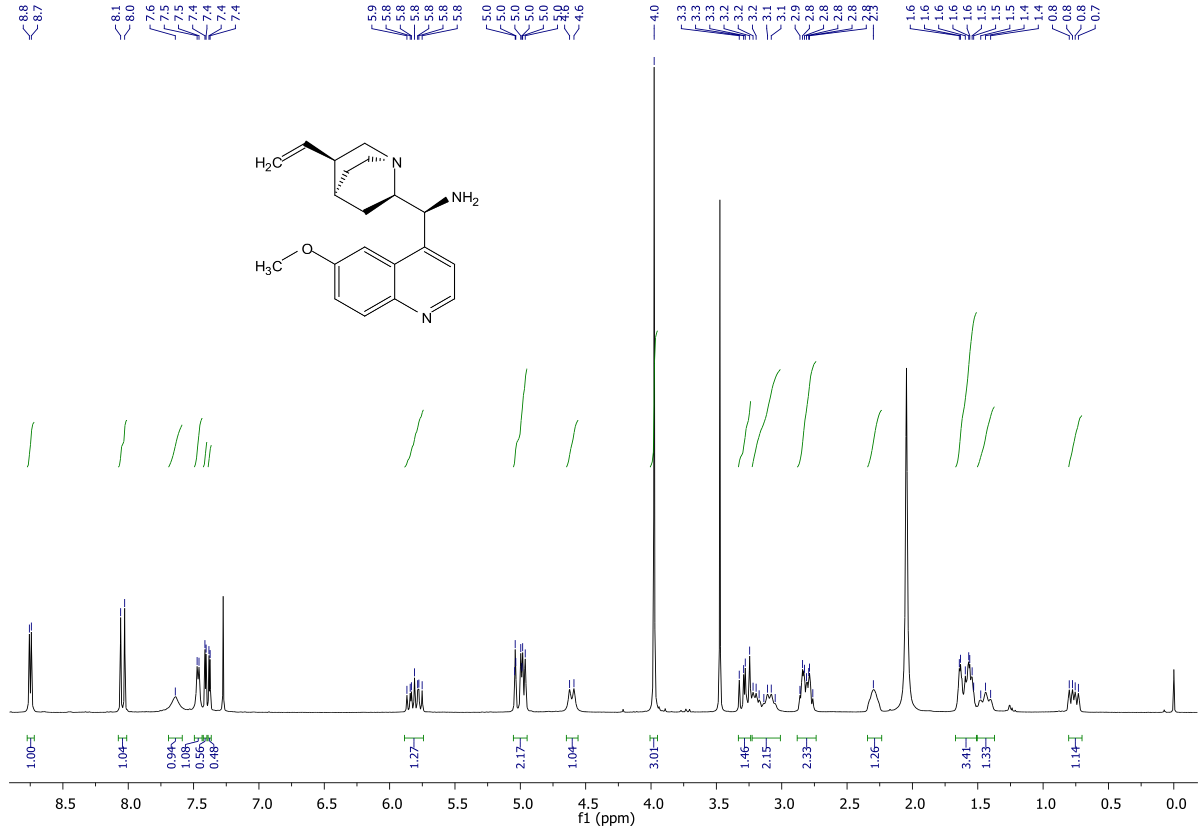

^{1}H NMR spectrum of **S4**

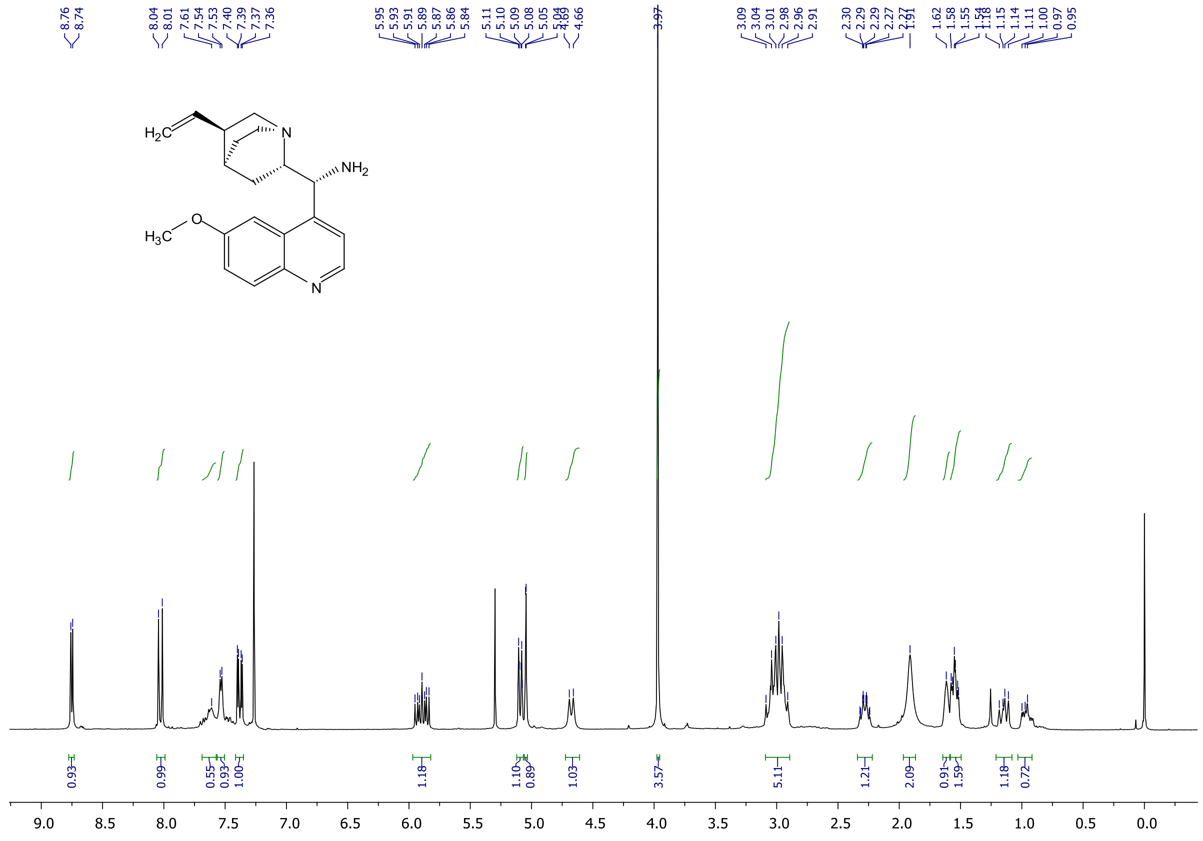

^{1}H NMR spectrum of **S5**

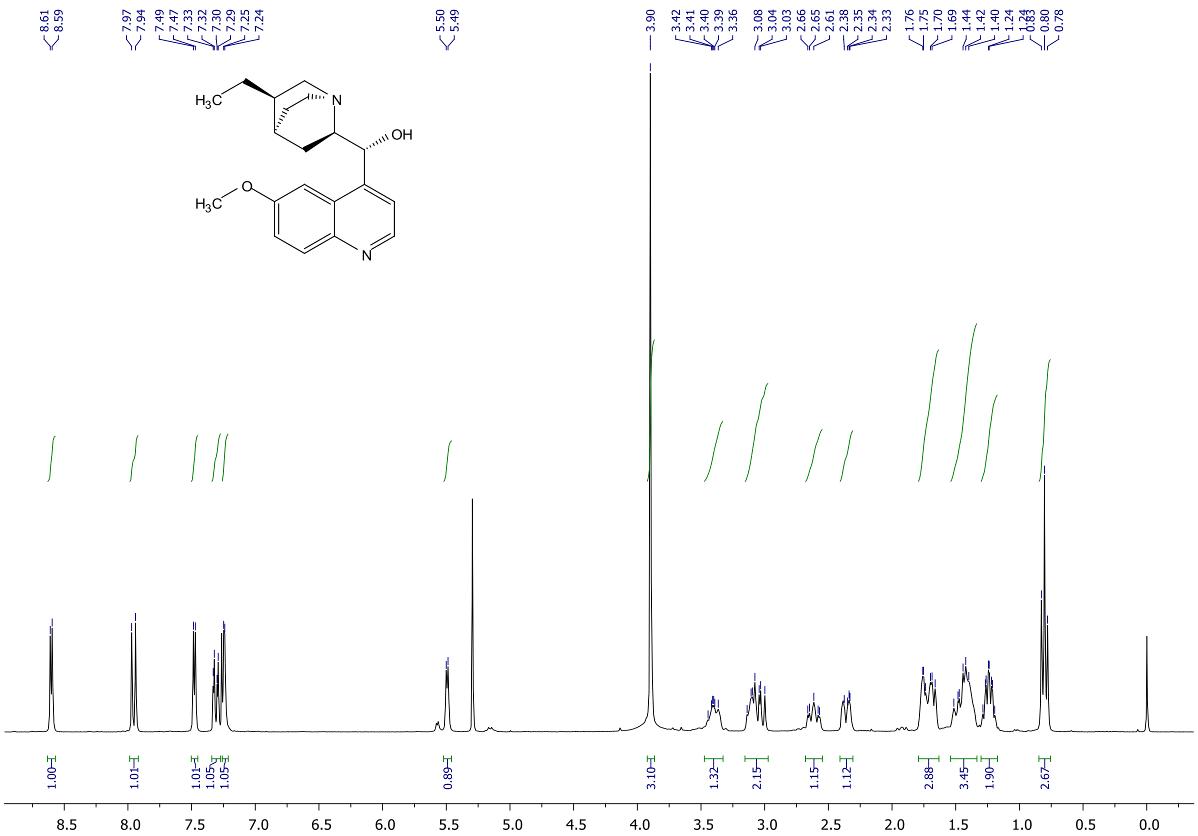

^{1}H NMR spectrum of **S6**

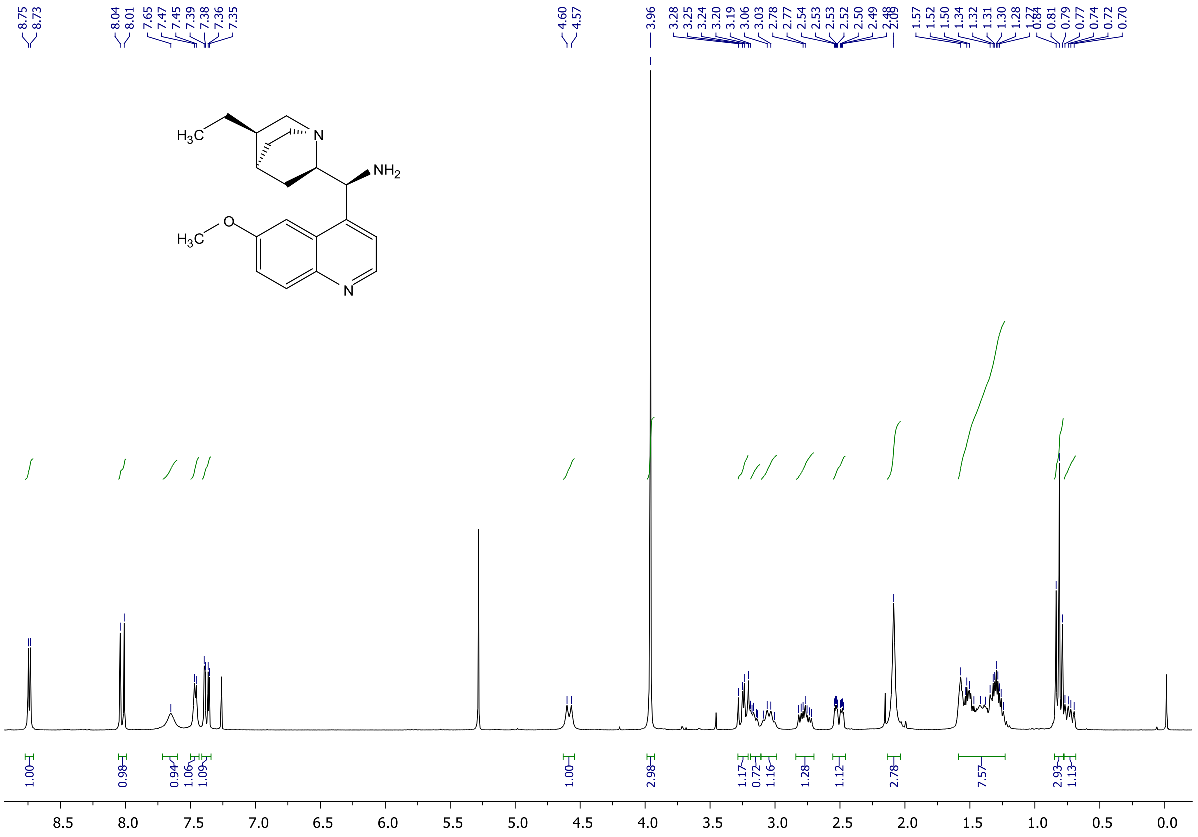

^{1}H NMR spectrum of **C1**

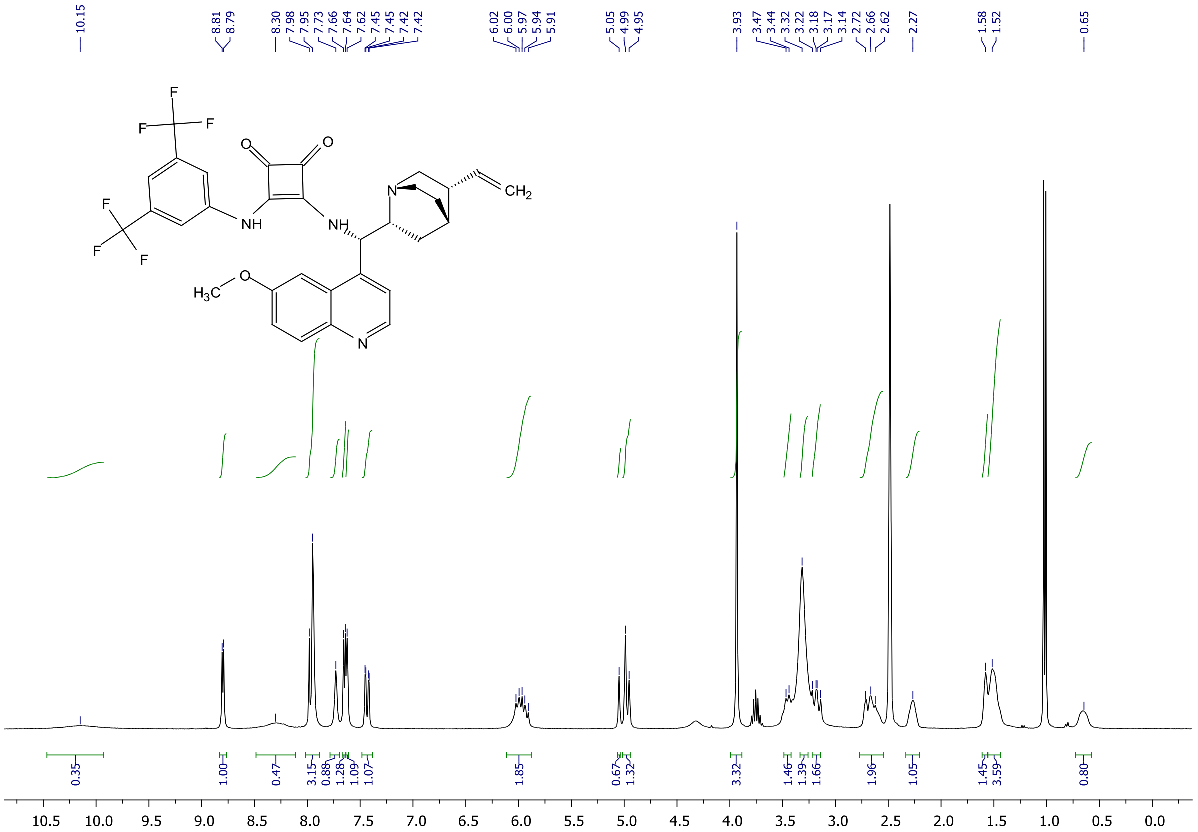

^{13}C NMR spectrum of **C1**

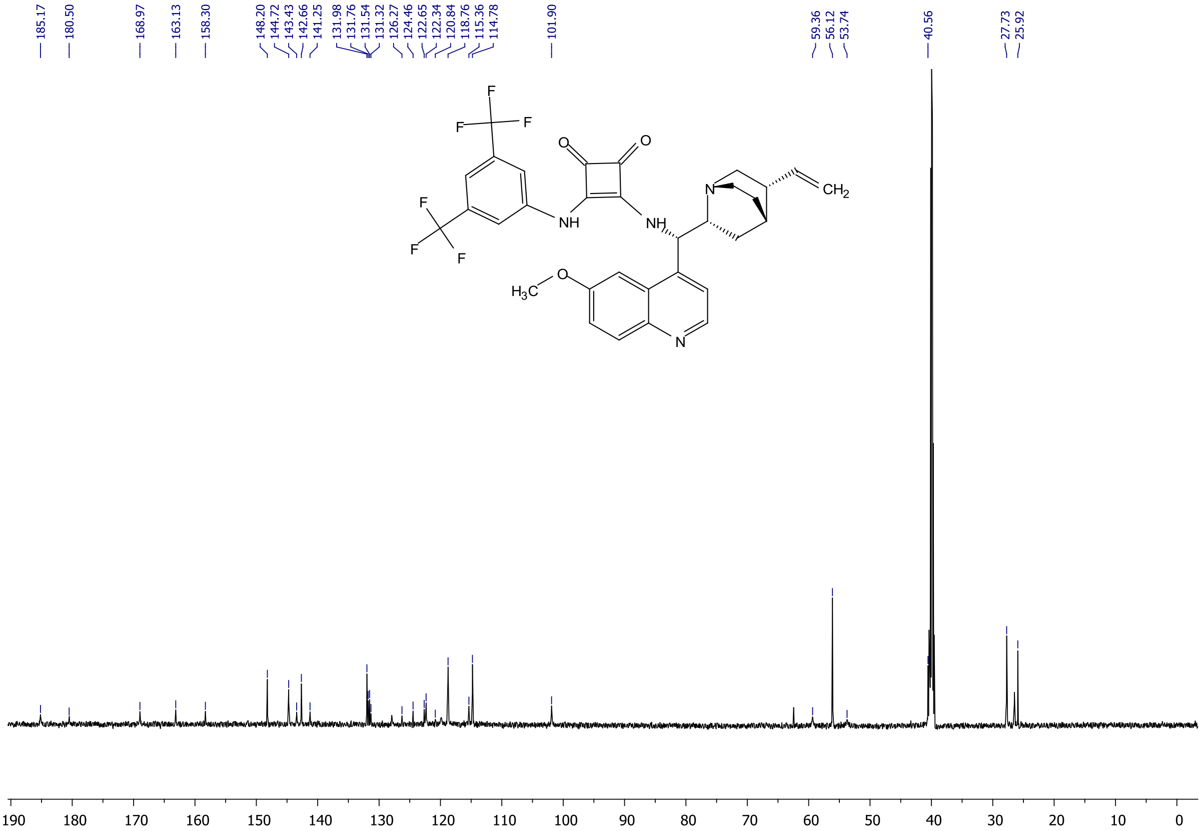

^{1}H NMR spectrum of **C2**

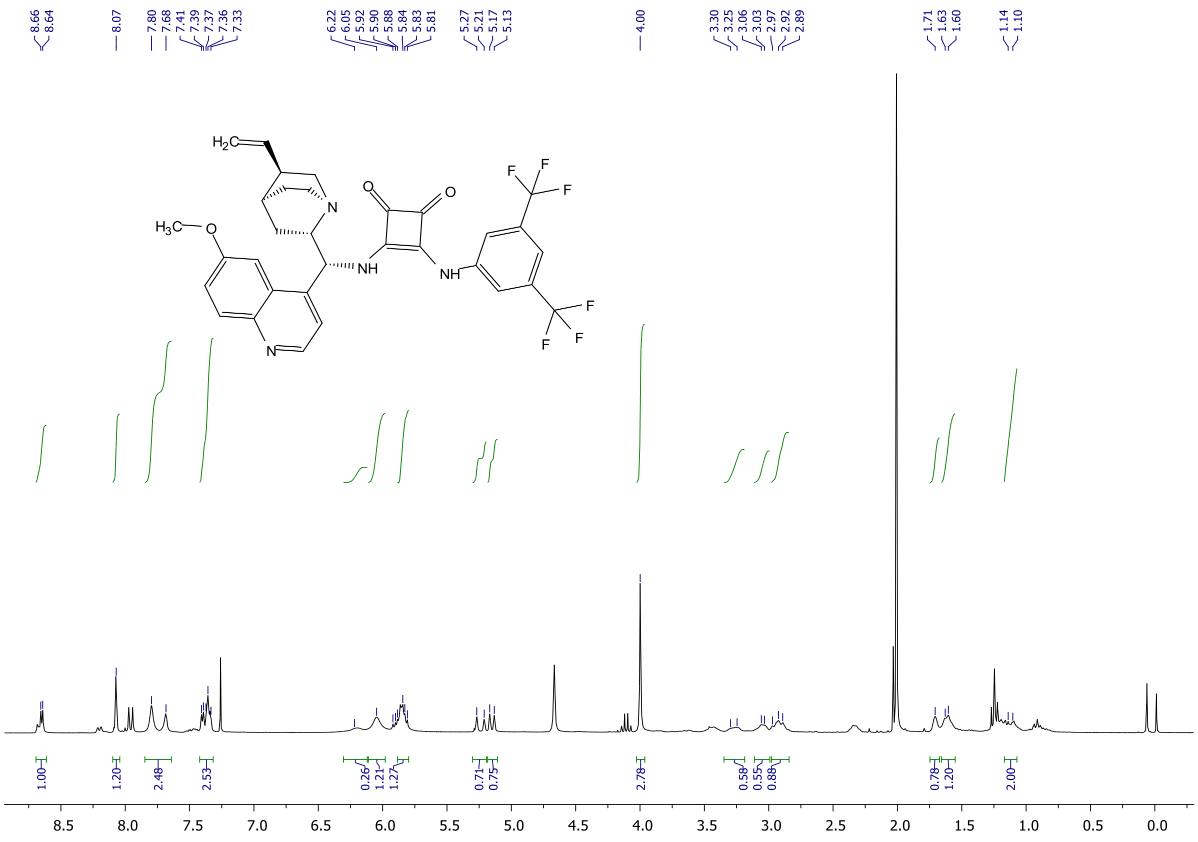

^{13}C NMR spectrum of **C2**

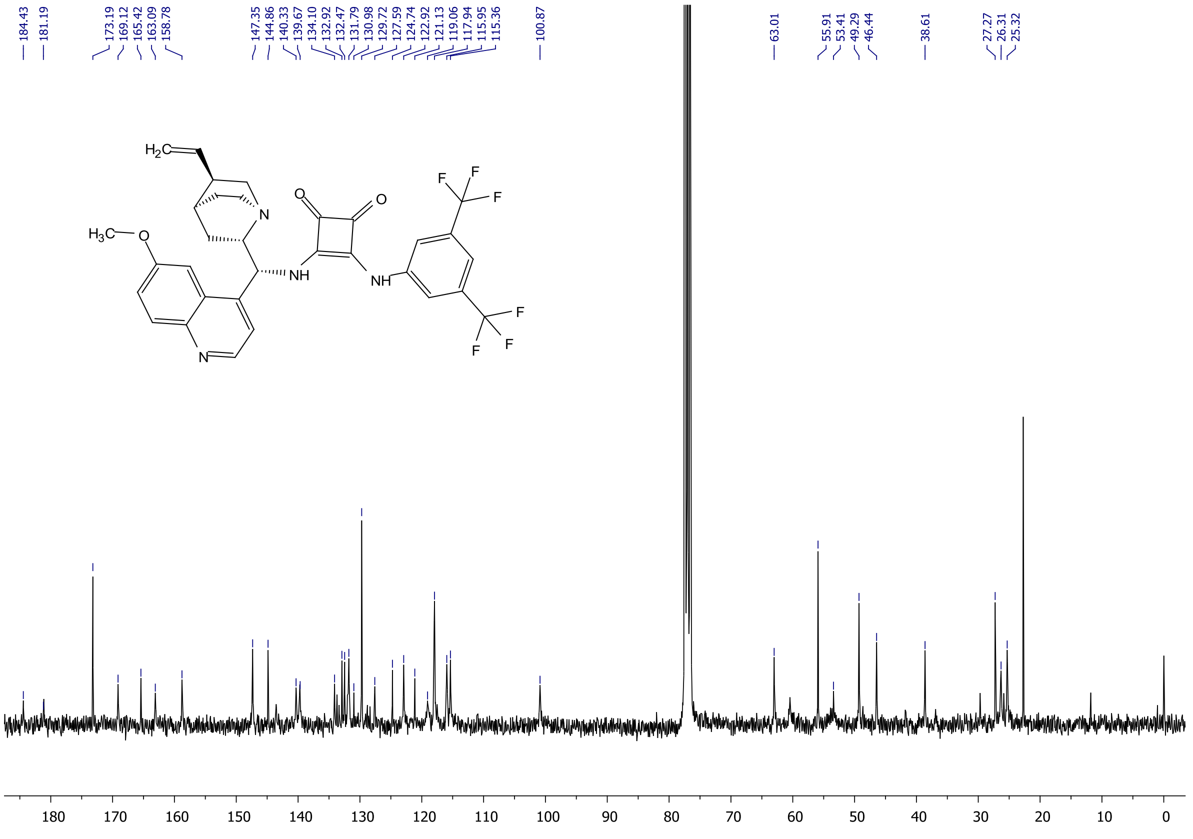

^{1}H NMR spectrum of
C3

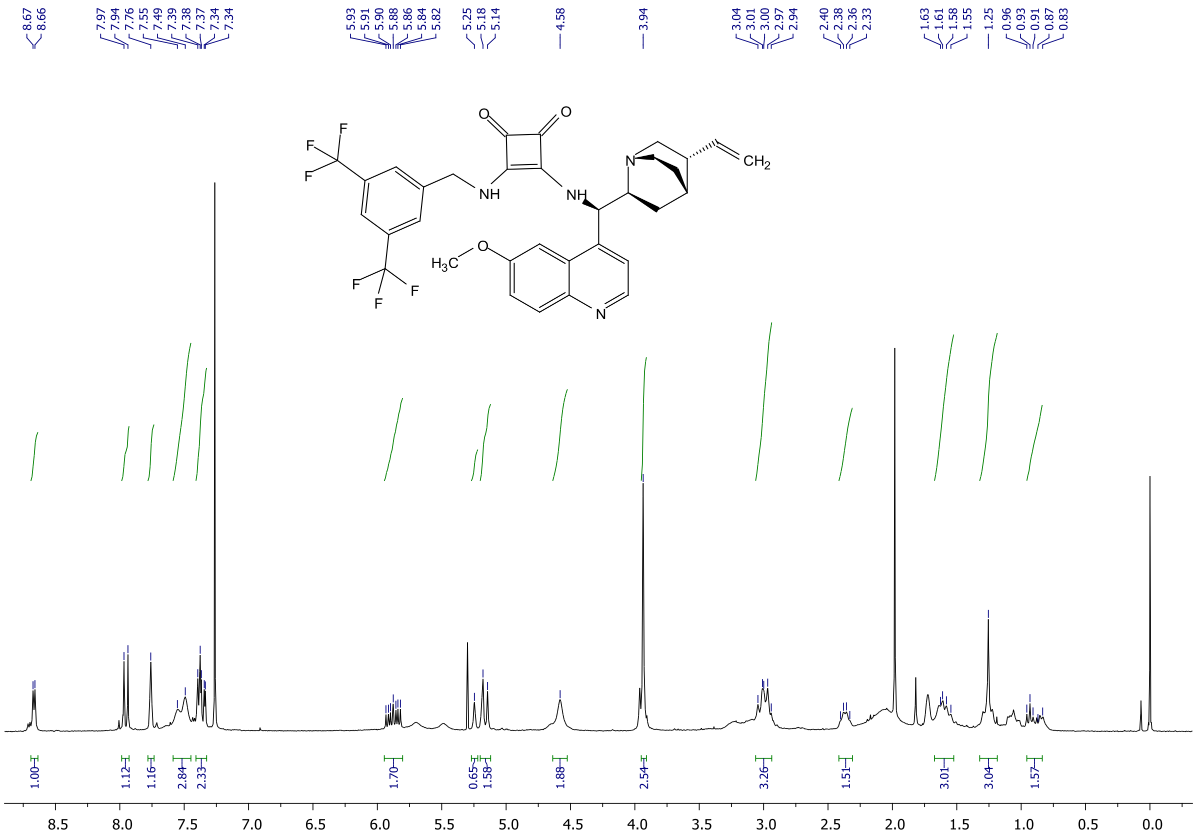

^{13}C NMR spectrum of **C3**

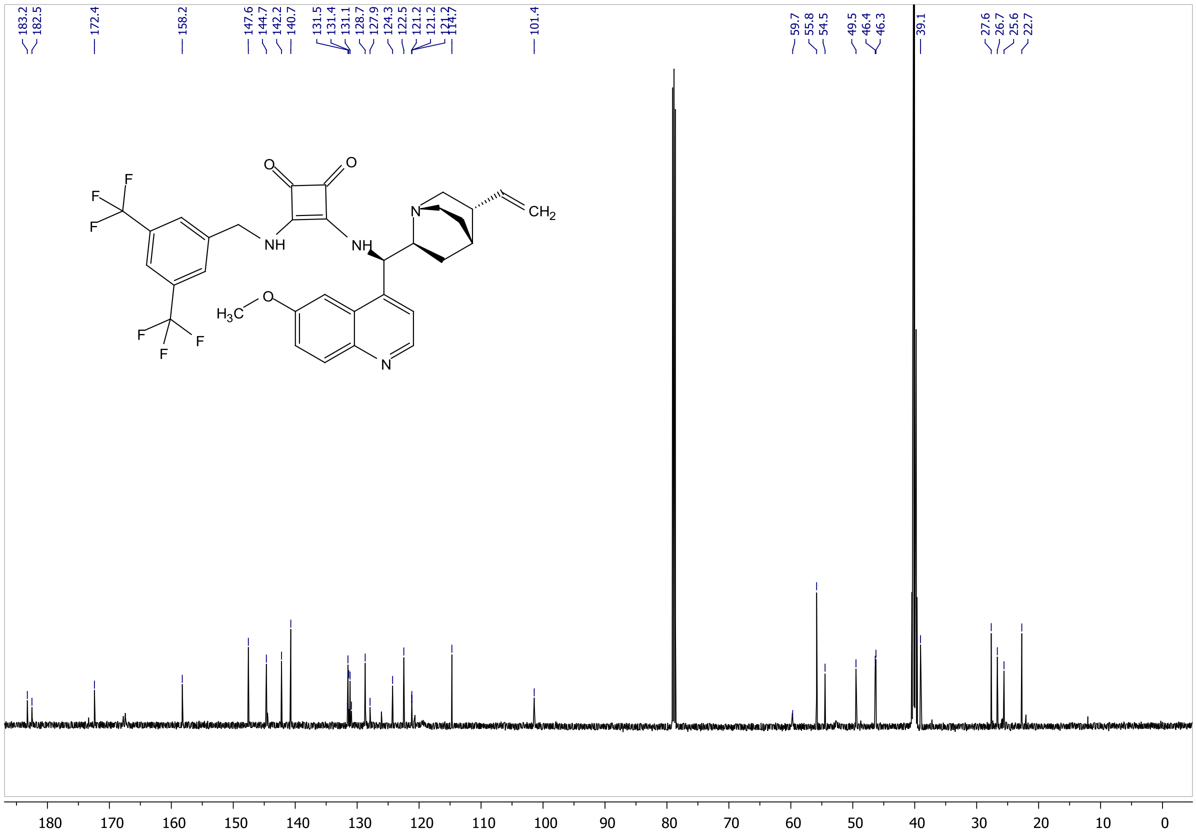

^{1}H NMR spectrum of **C4**

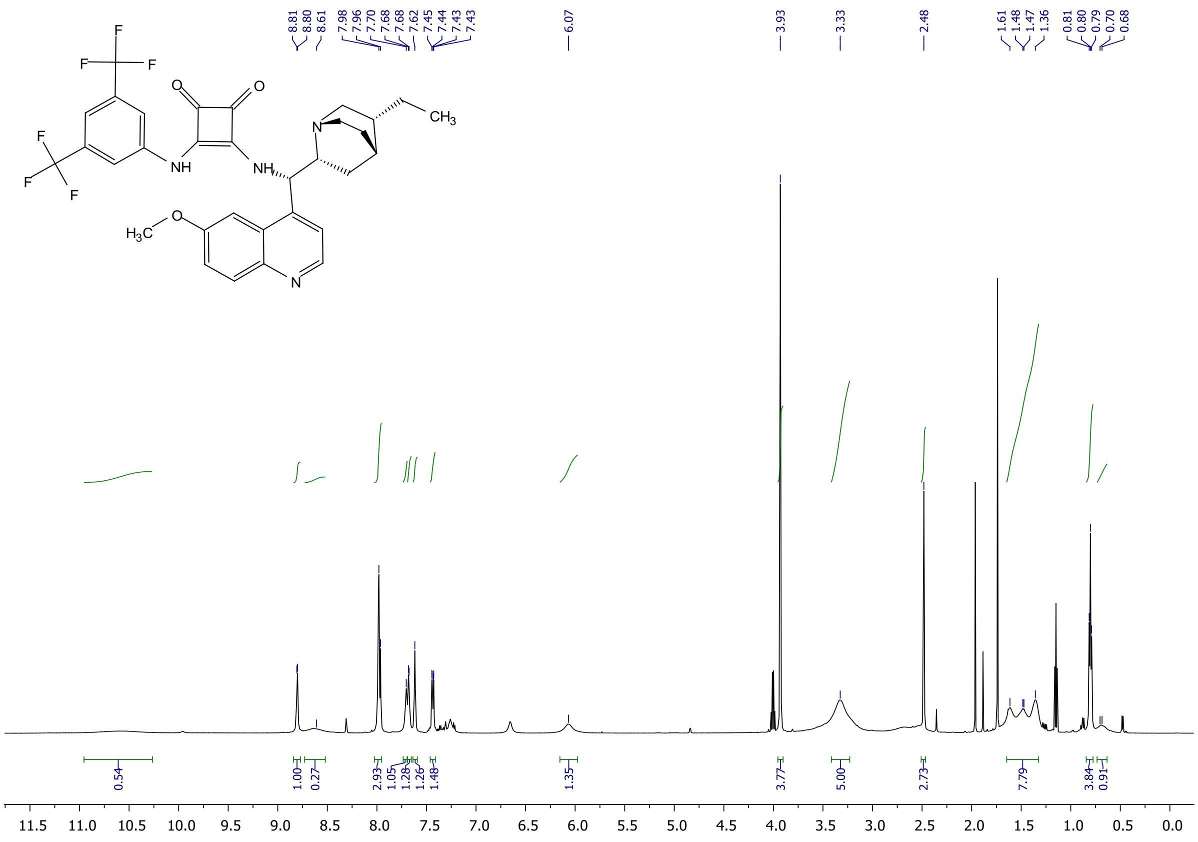

^{13}C NMR spectrum of **C4**

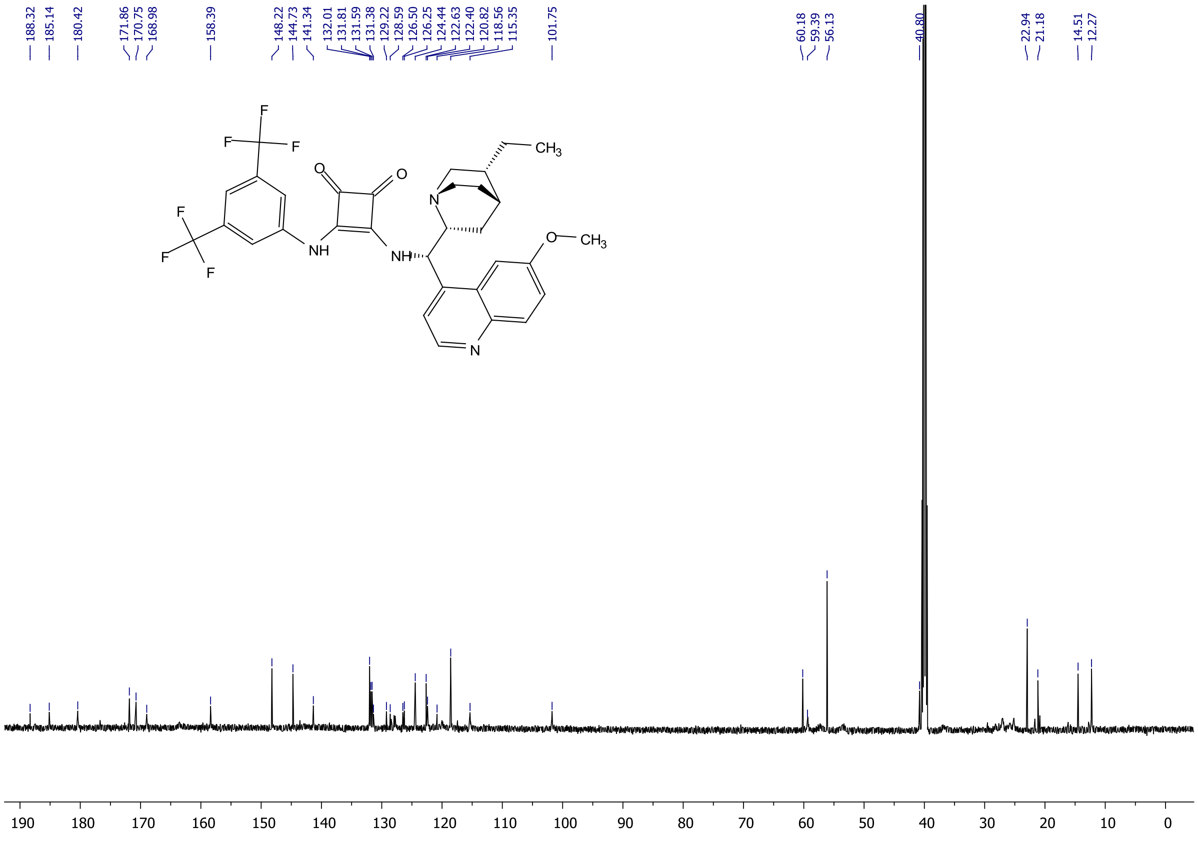

^{1}H NMR spectrum of (*S*,*S*)-**S7**

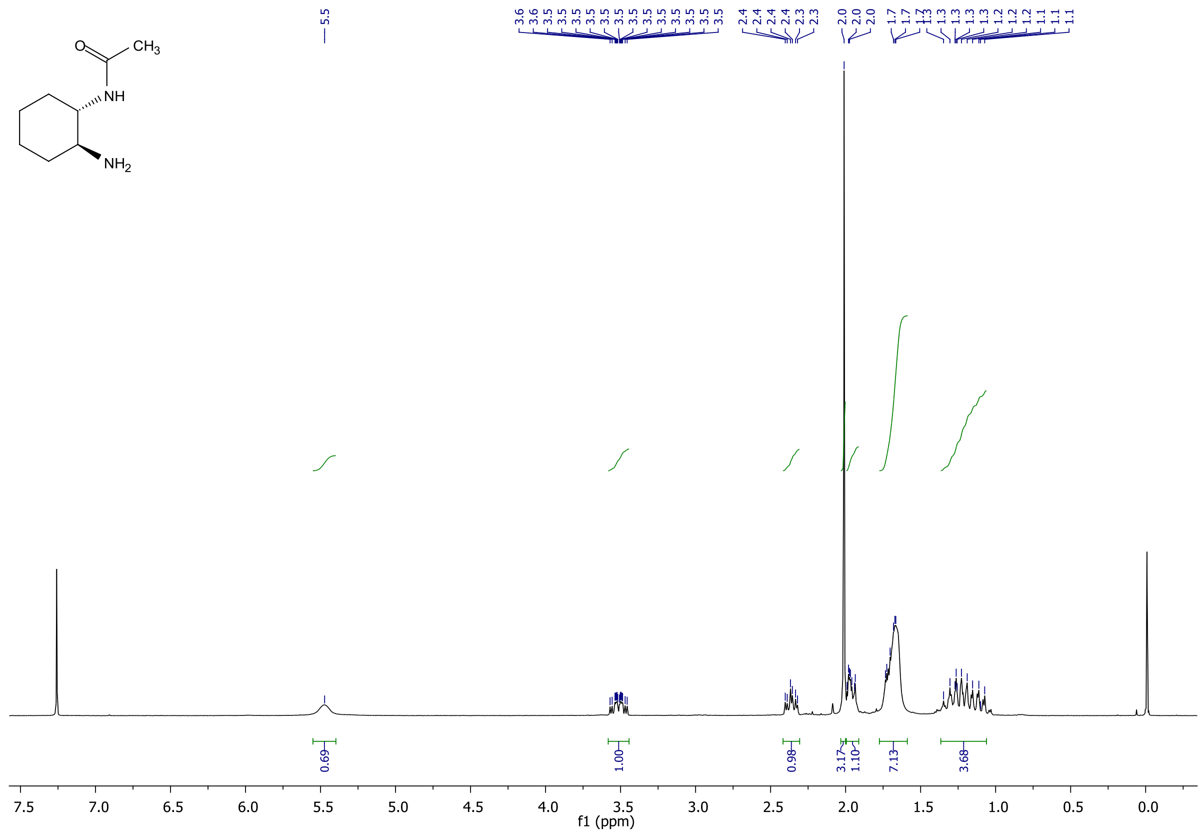

^{13}C NMR spectrum of (*S*,*S*)-**S7**

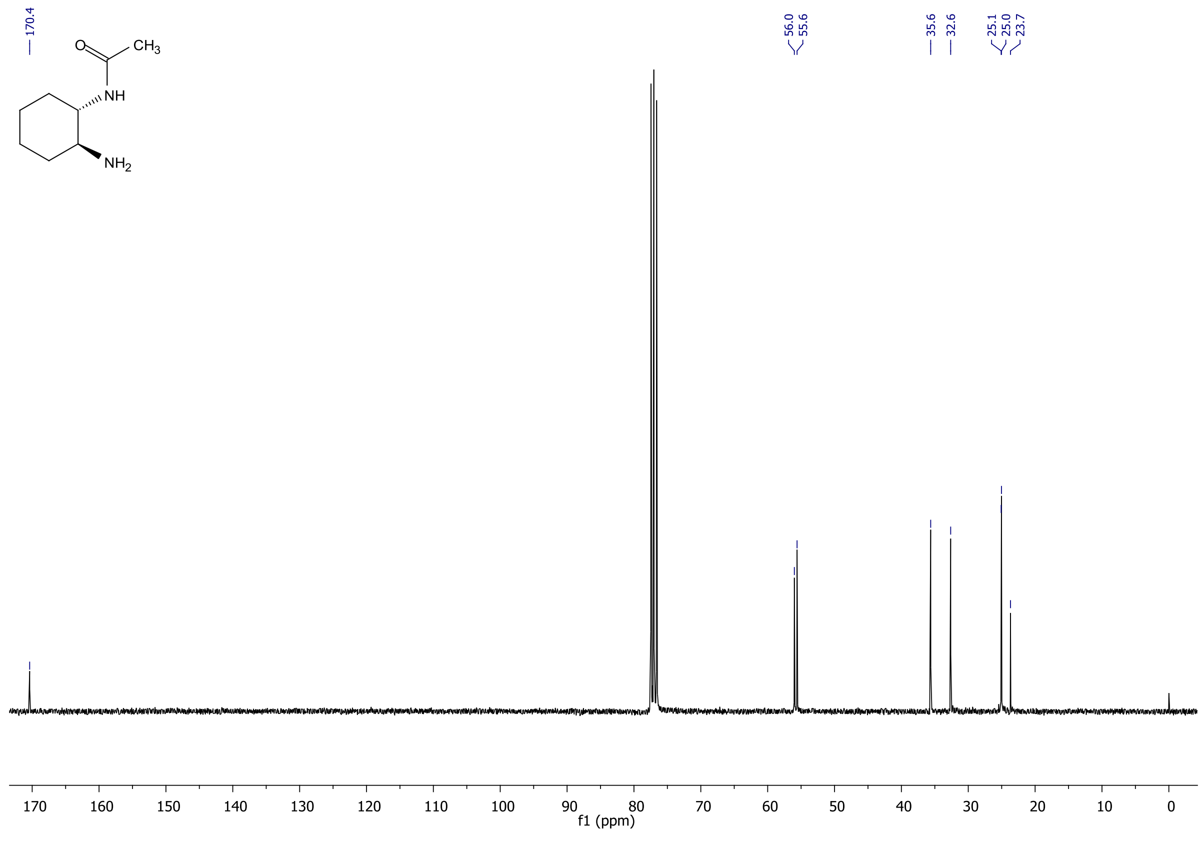

^{1}H NMR spectrum of (*S*,*S*)-**S8**

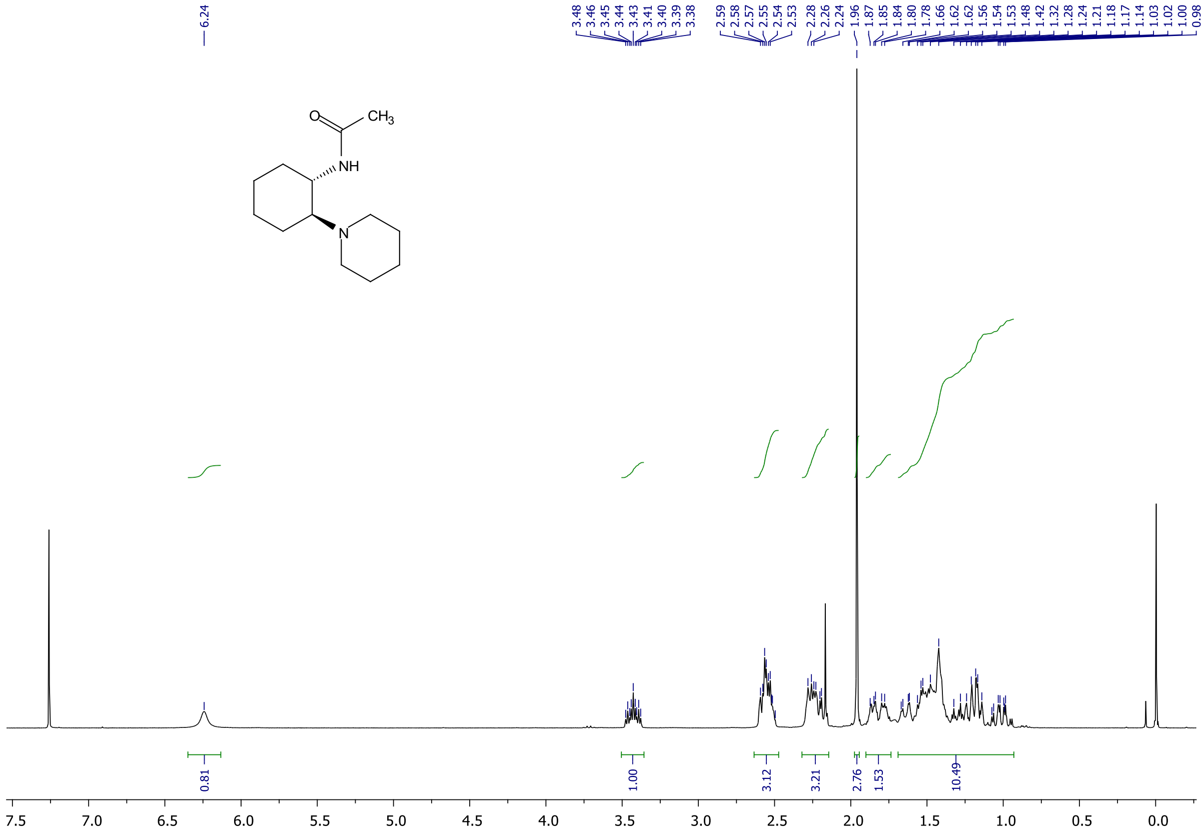

^{1}H NMR spectrum of (*S*,*S*)-**12**

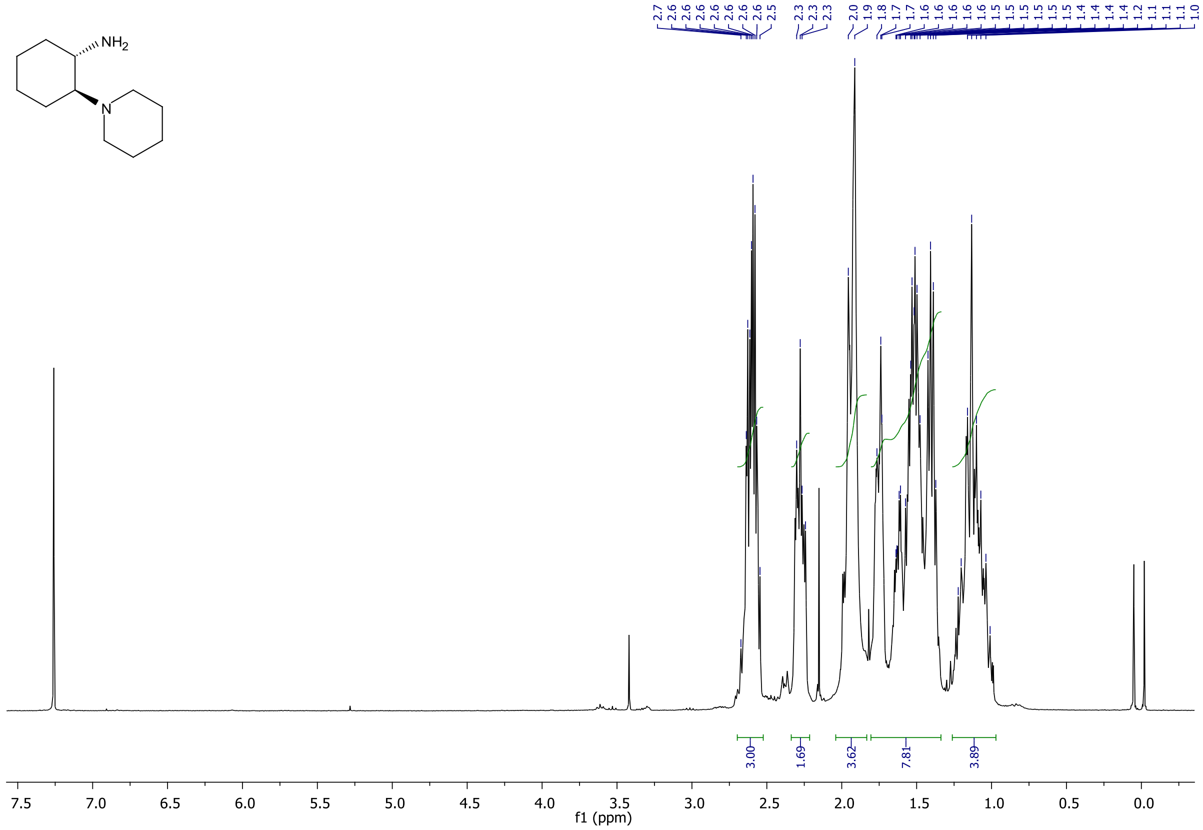

^{1}H NMR spectrum of (*R*,*R*)-**S7**

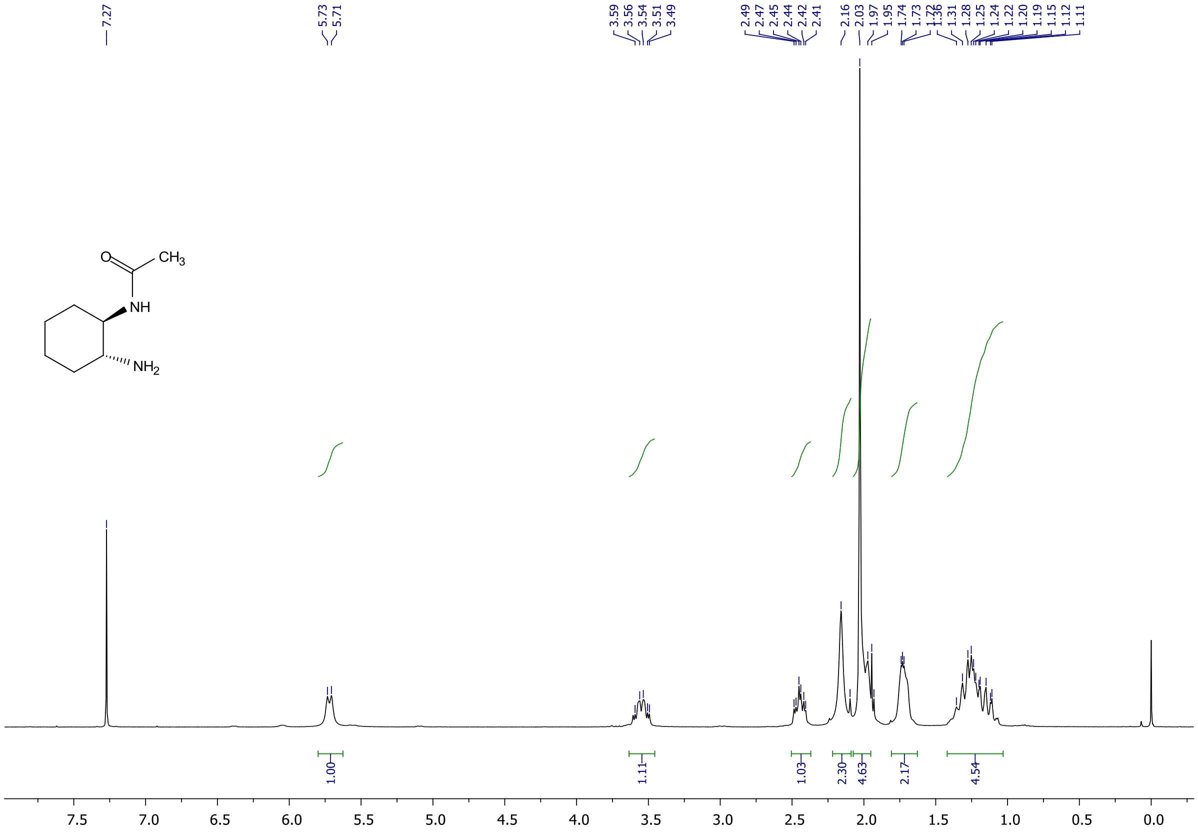

^{1}H NMR spectrum of (*R*,*R*)-**S8**

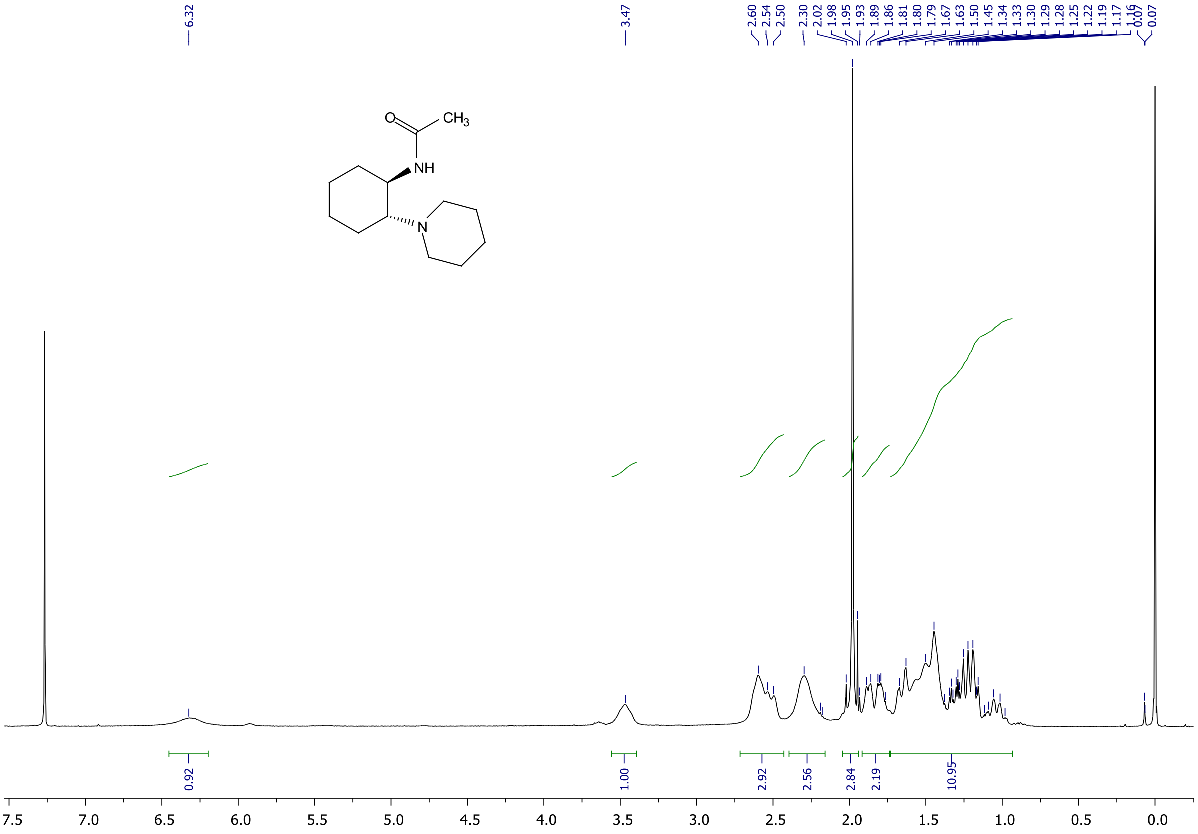

^{1}H NMR spectrum of (*R*,*R*)-**12**

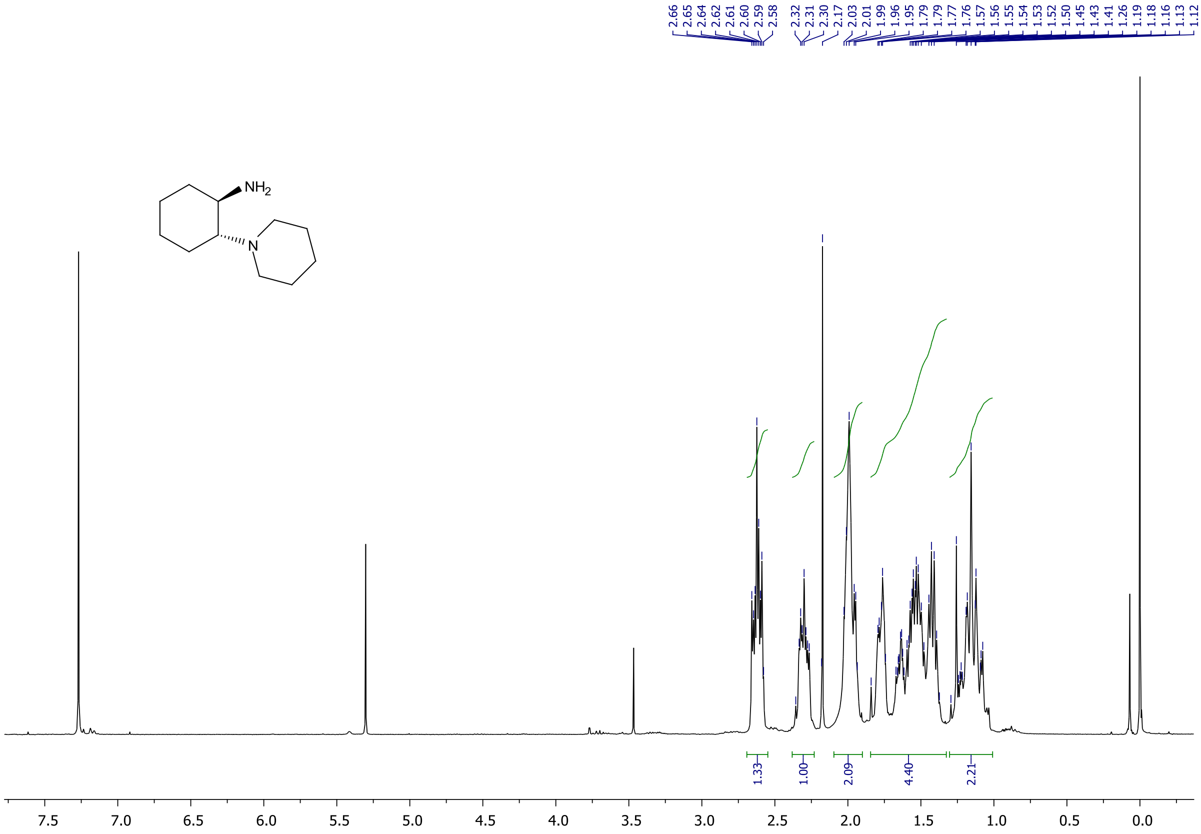

^{1}H NMR spectrum of (*S*,*S*)-**C5**

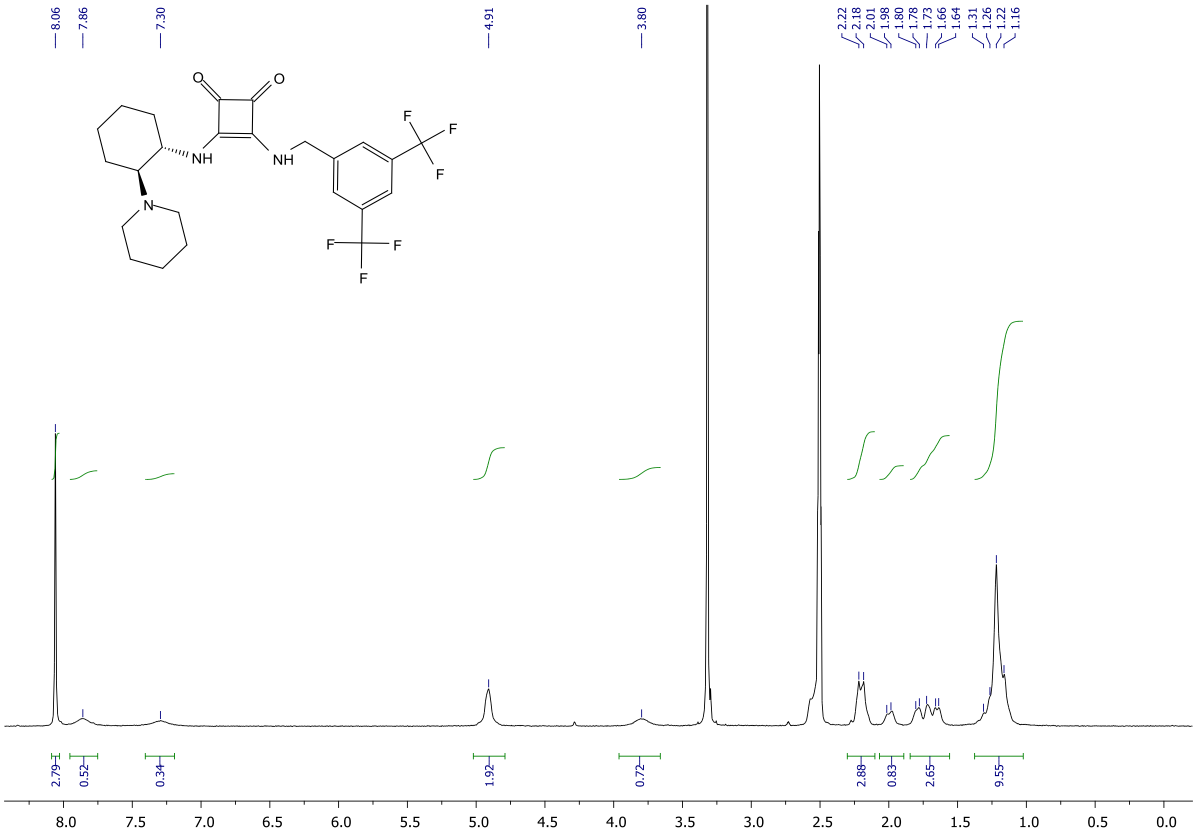

^{13}C NMR spectrum of (*S*,*S*)-**C5**

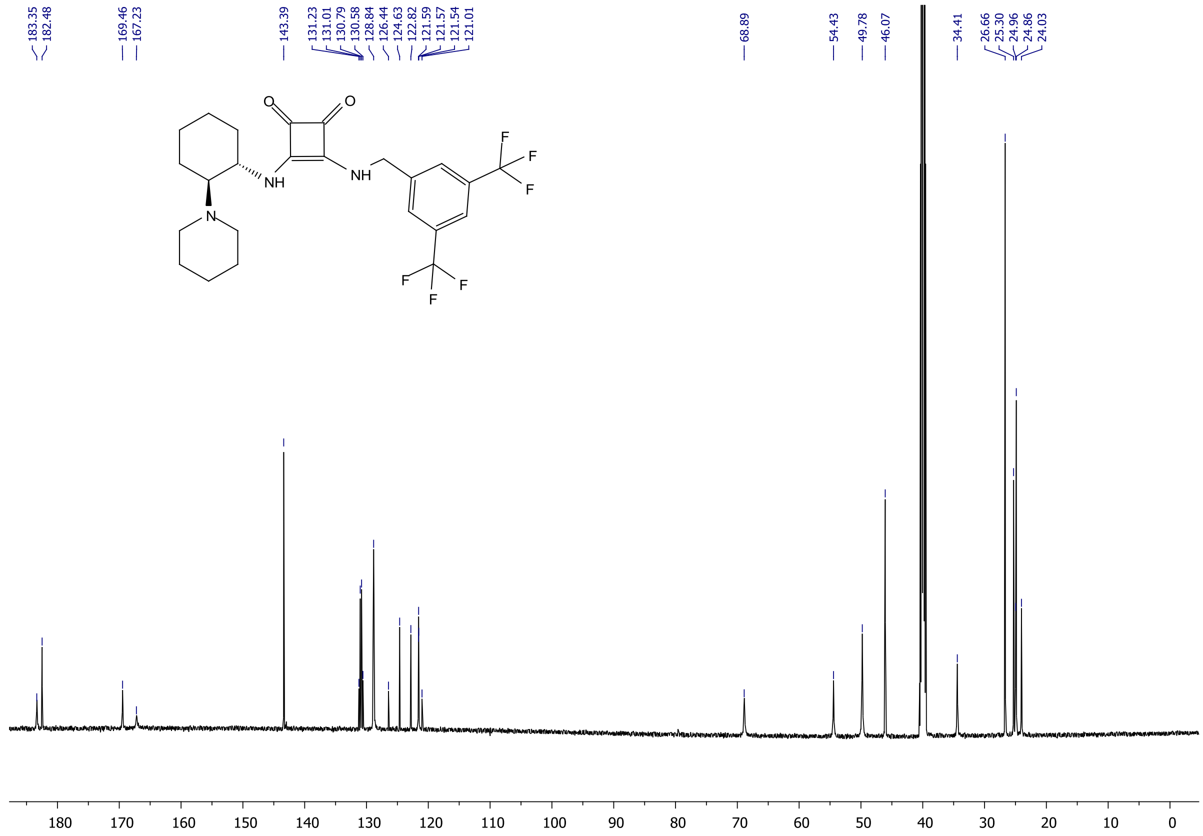

^{1}H NMR spectrum of (*R*,*R*)-**C5**

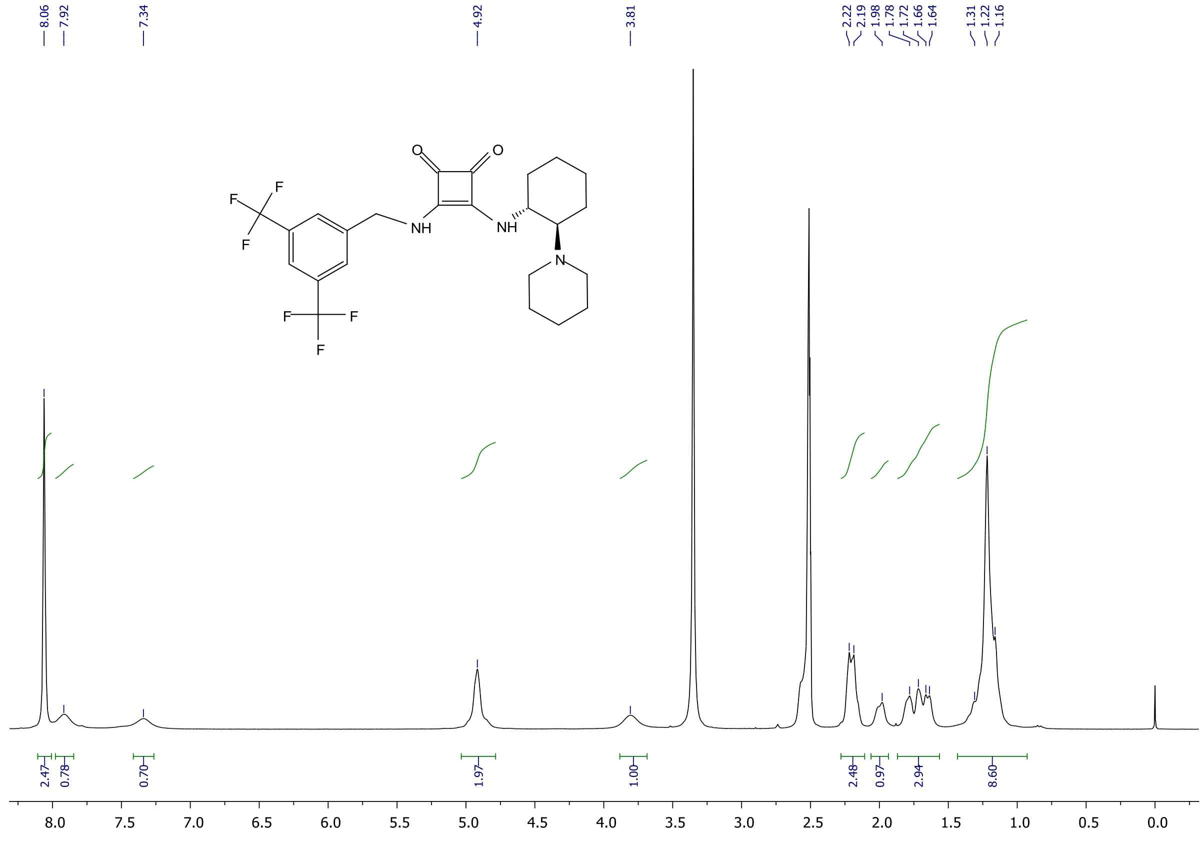

^{13}C NMR spectrum of (*R*,*R*)-**C5**

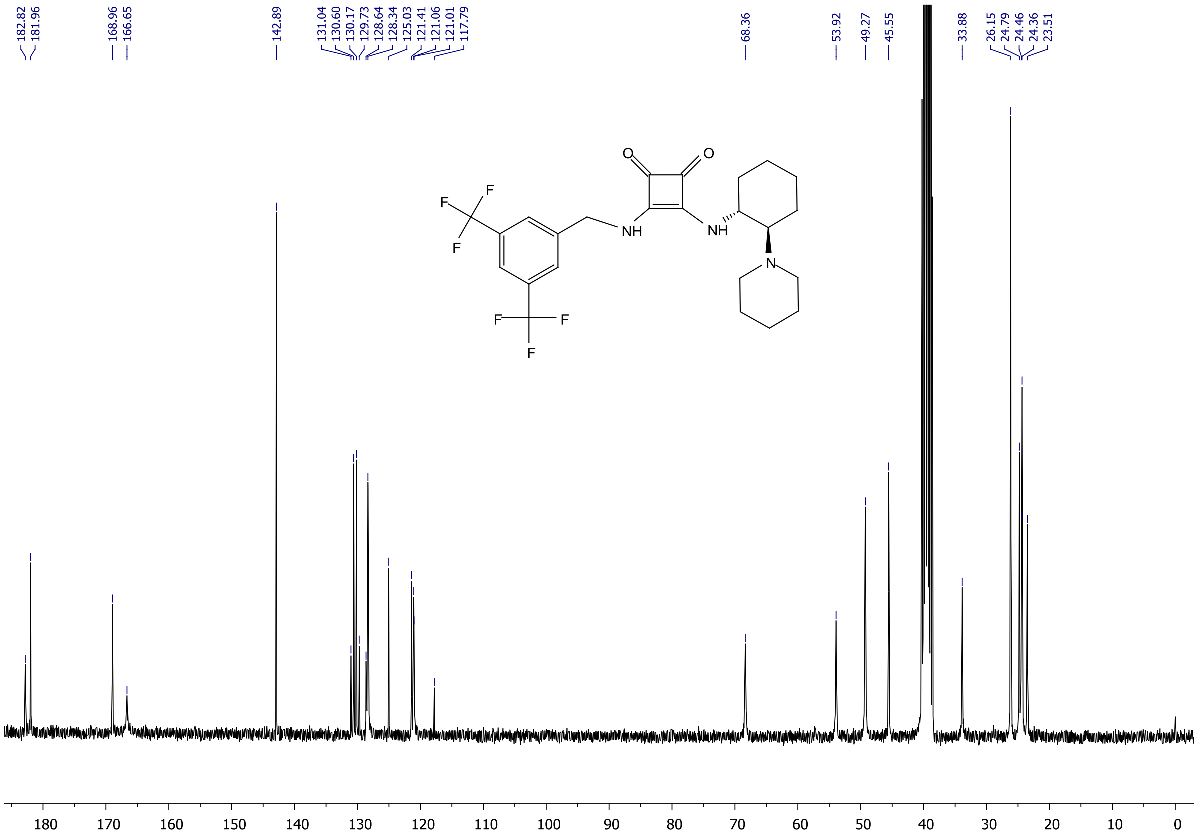

^{1}H NMR spectrum of **C6**

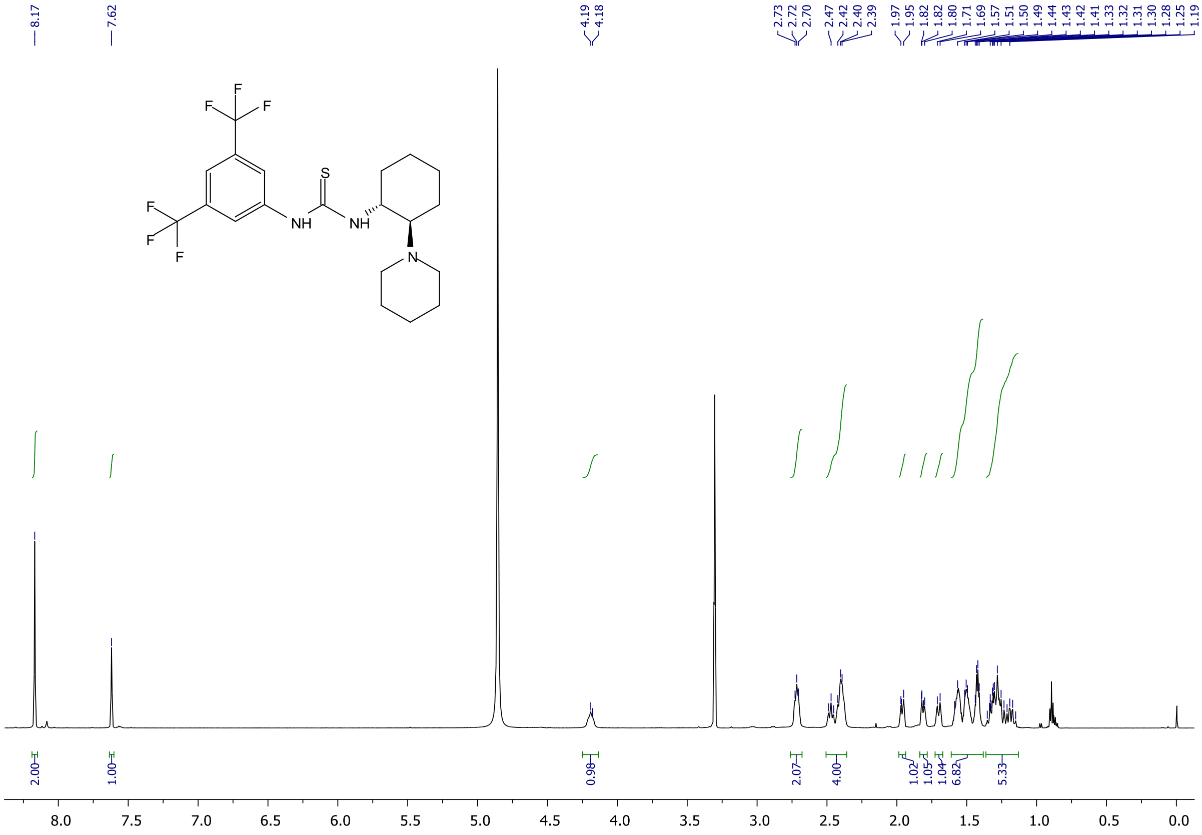

^{13}C NMR spectrum of **C6**

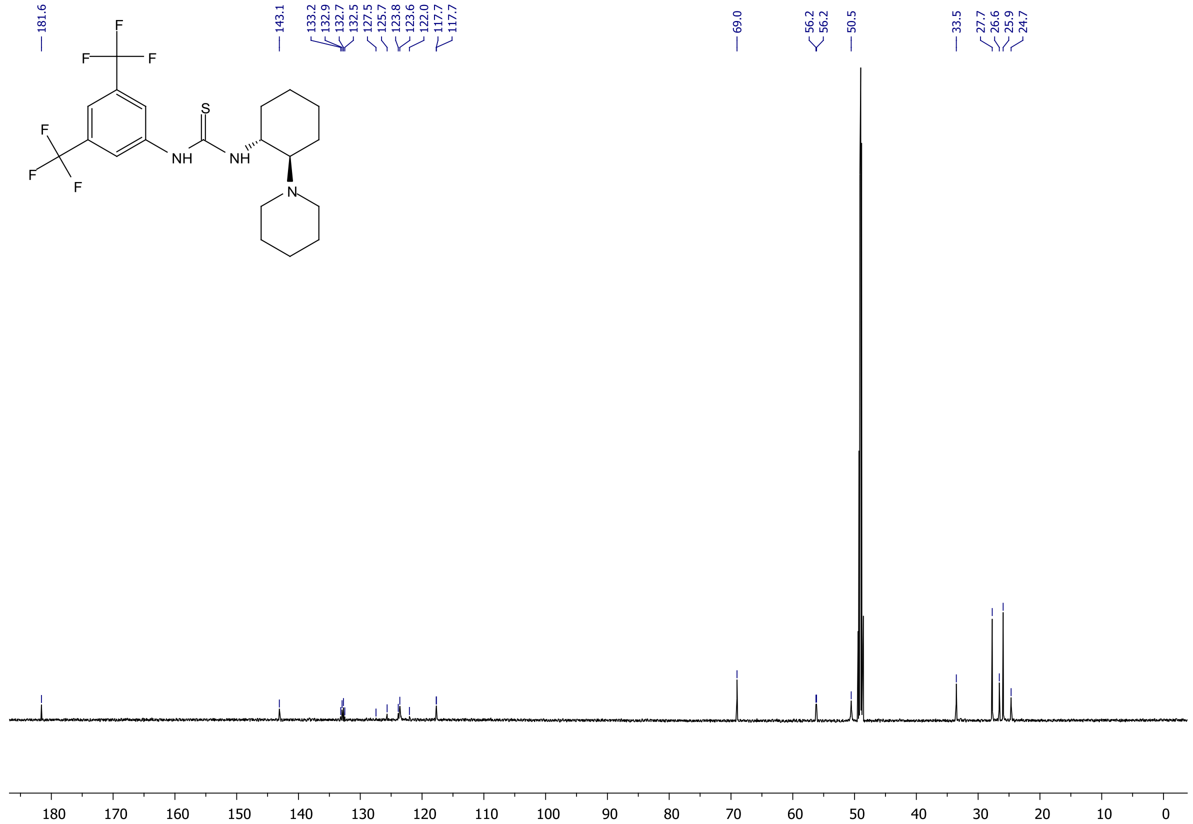

^{1}H NMR spectrum of (*R*,*R*)-**C7**

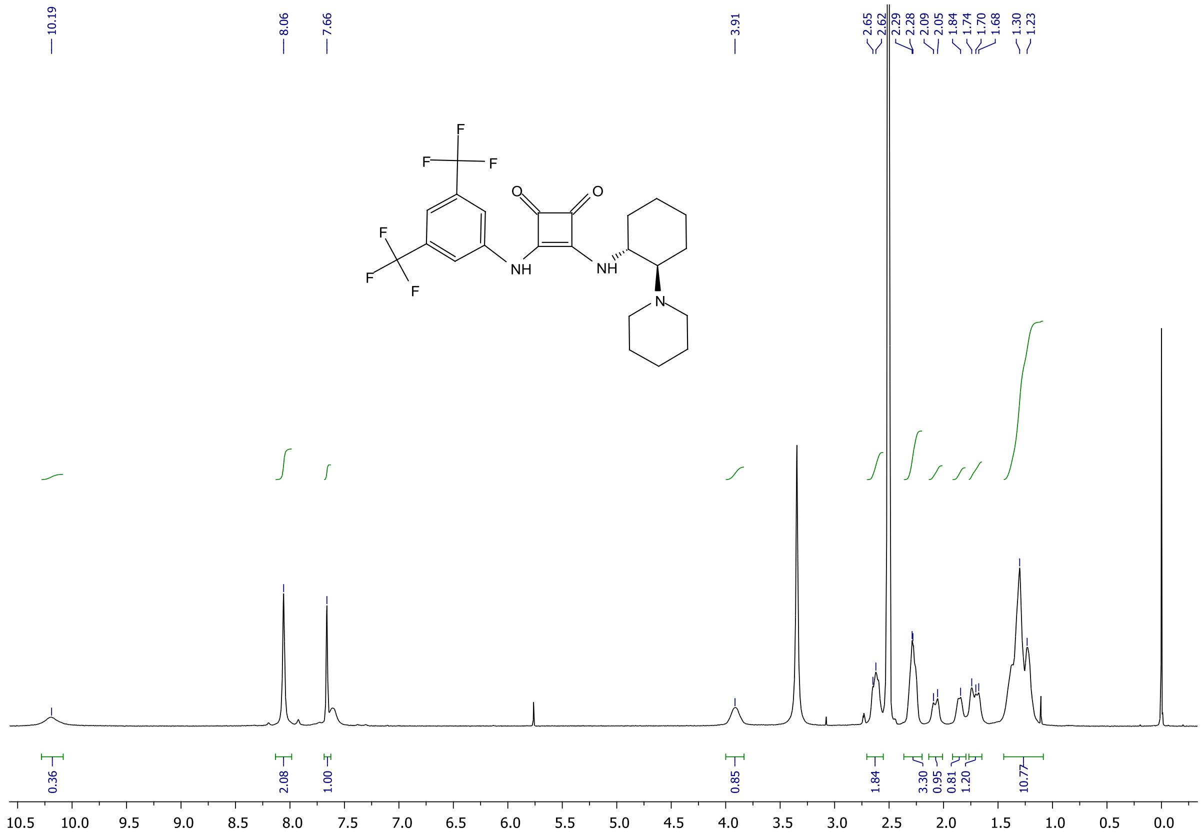

^{13}C NMR spectrum of (*R*,*R*)-**C7**

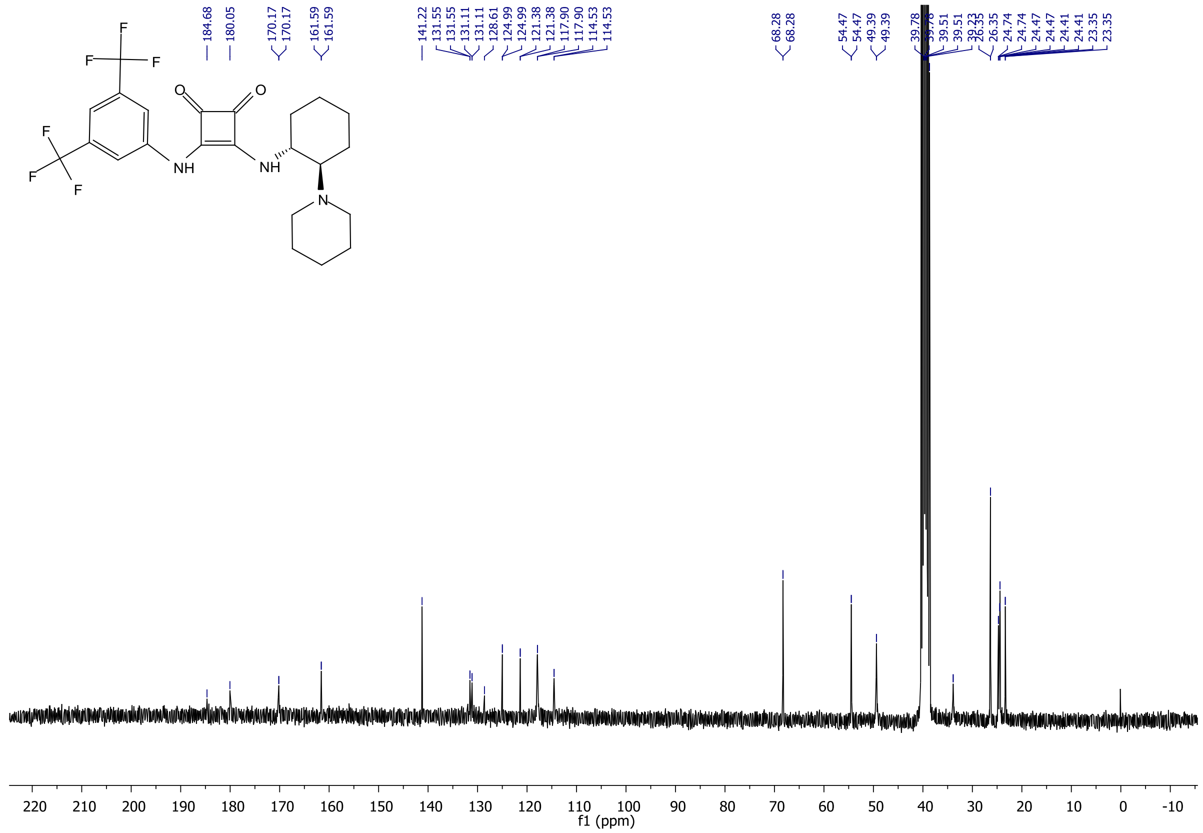

^{1}H NMR spectrum of **S9**

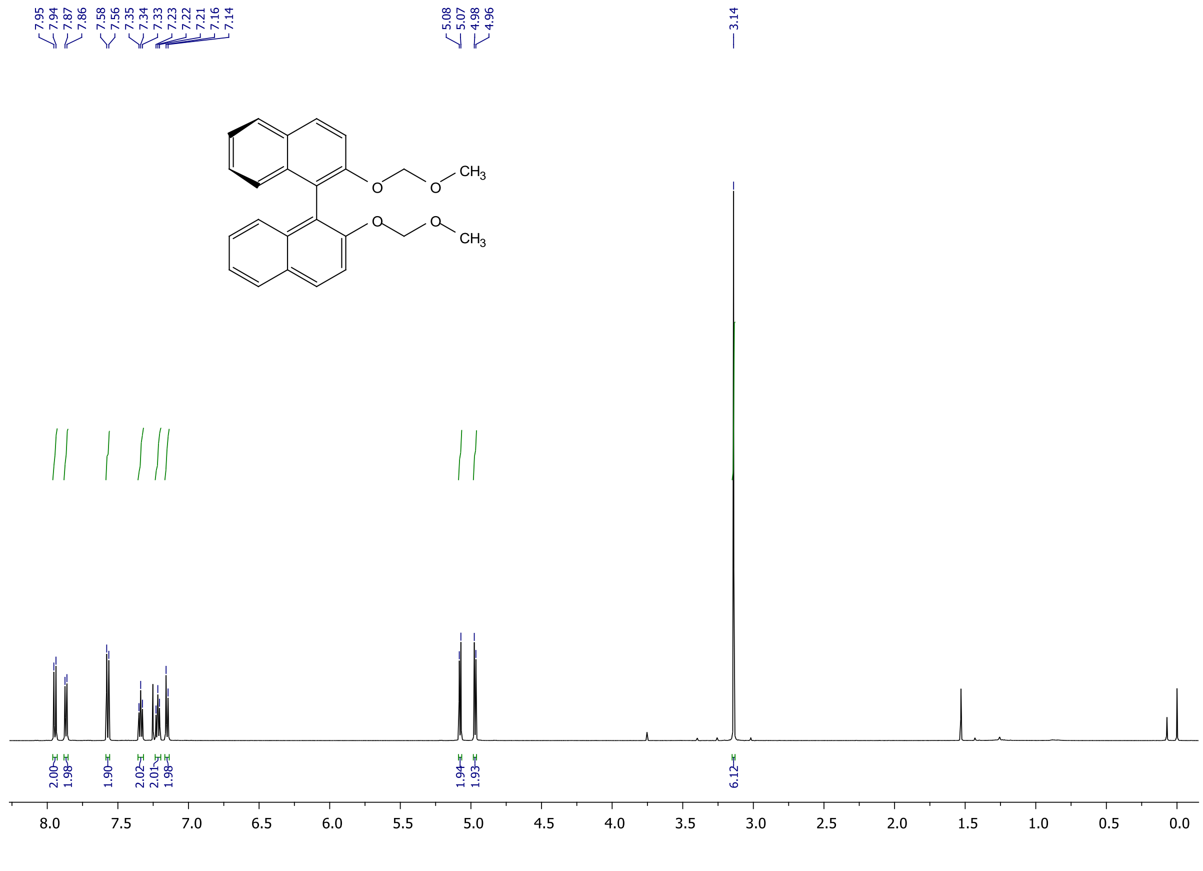

^{1}H NMR spectrum of
S10

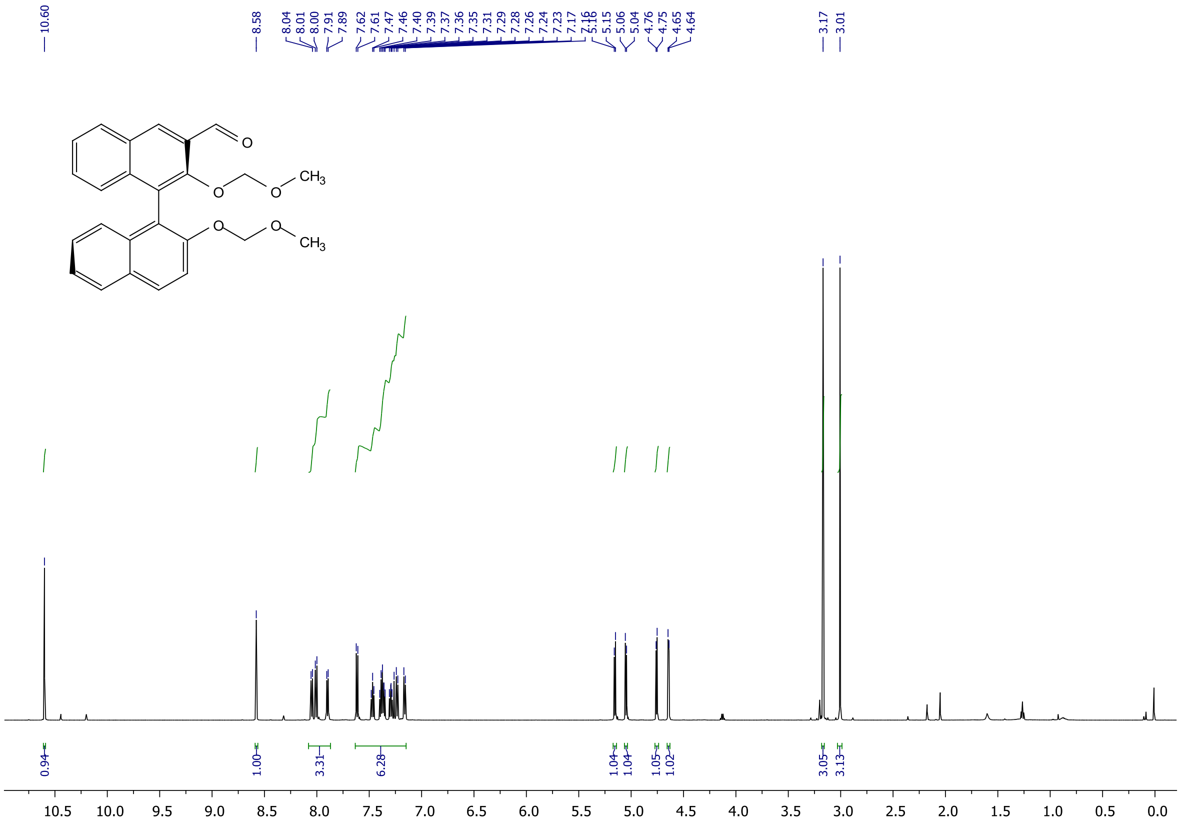

^{1}H NMR spectrum of **S11**

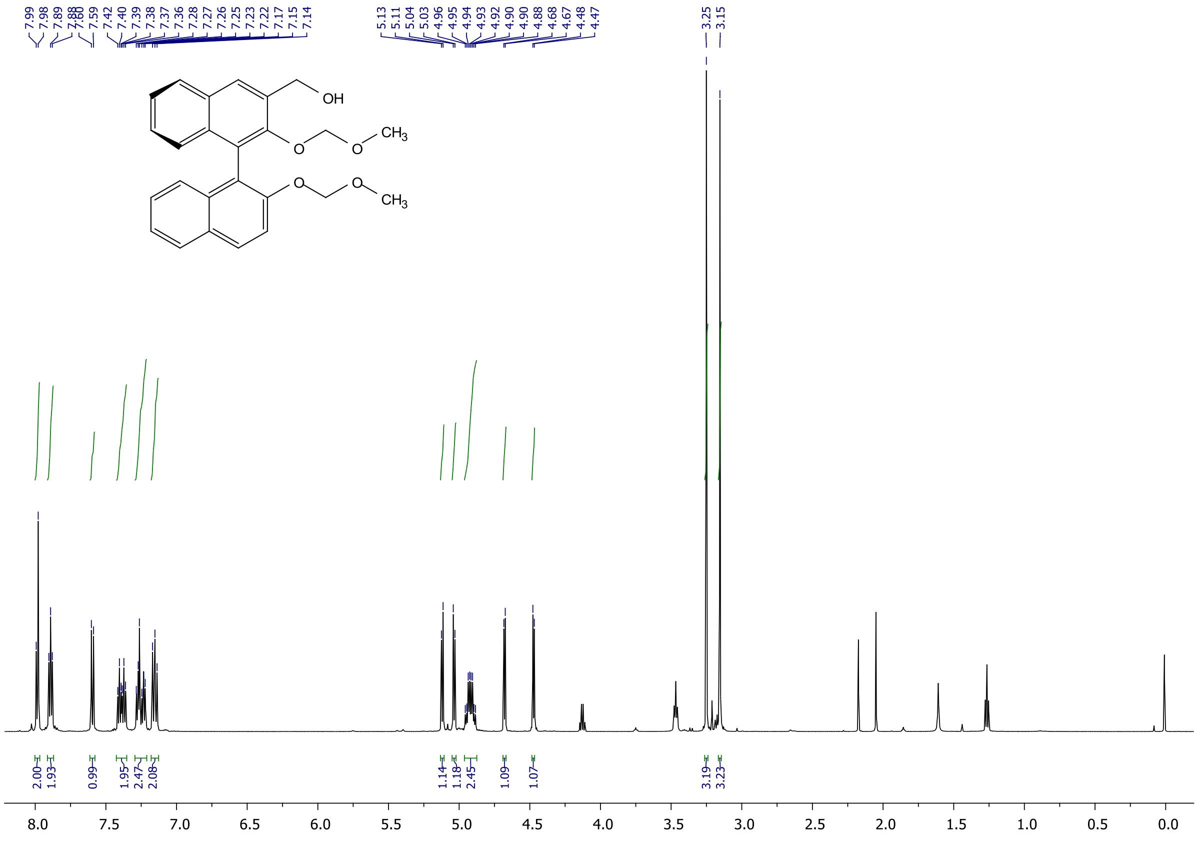

^{1}H NMR spectrum of **S12**

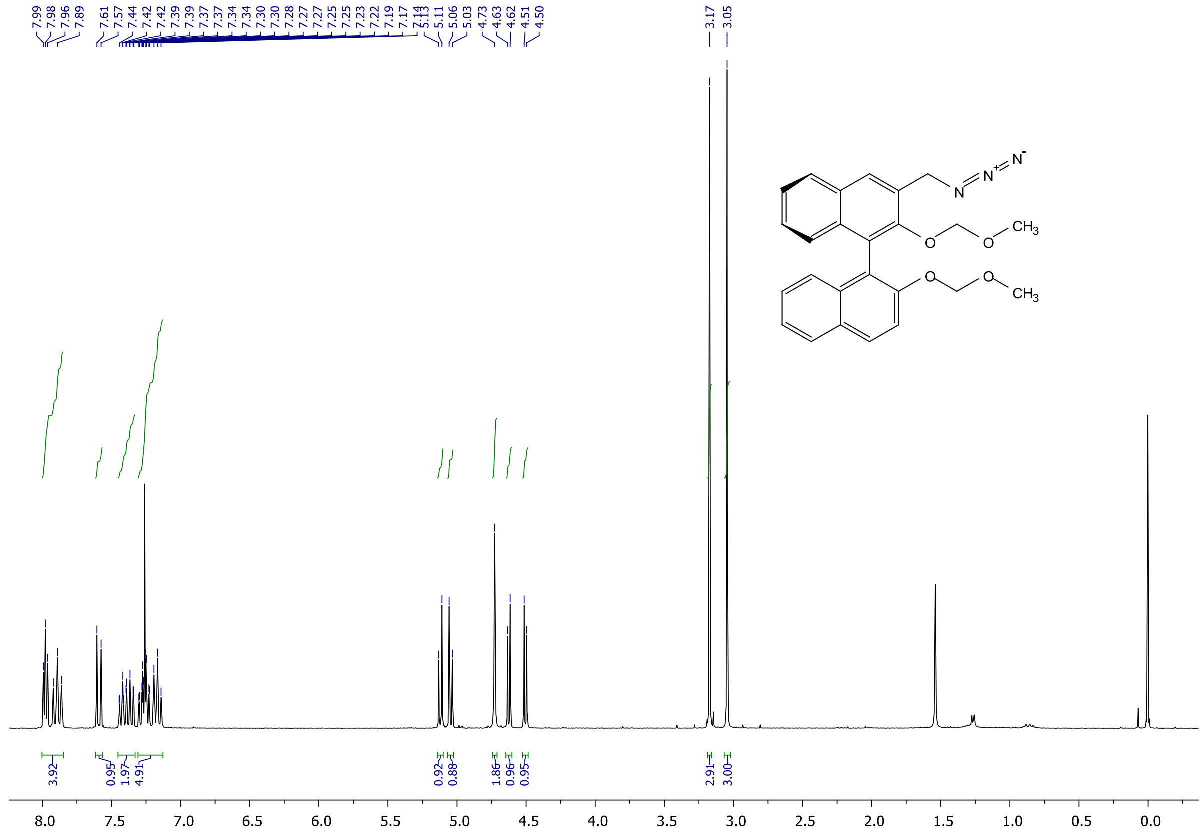

^{1}H NMR spectrum of **9**

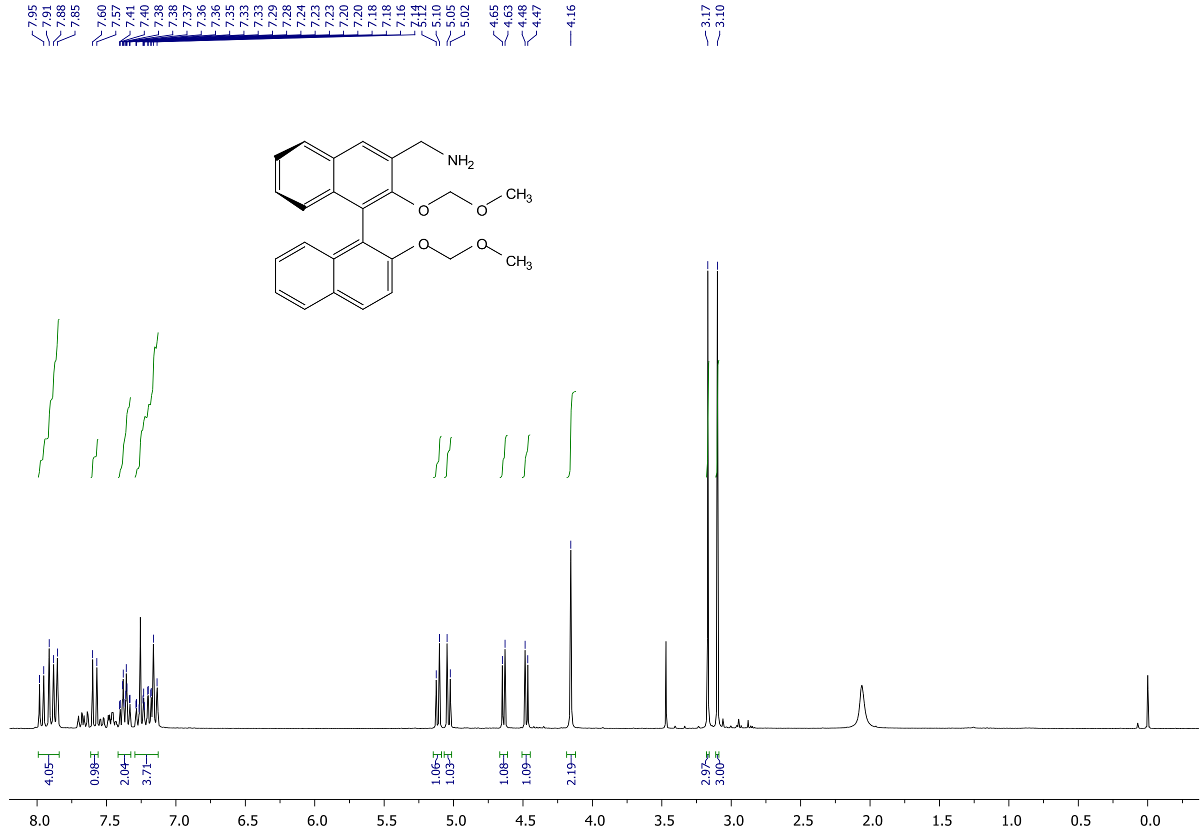

^{1}H NMR spectrum of **11**

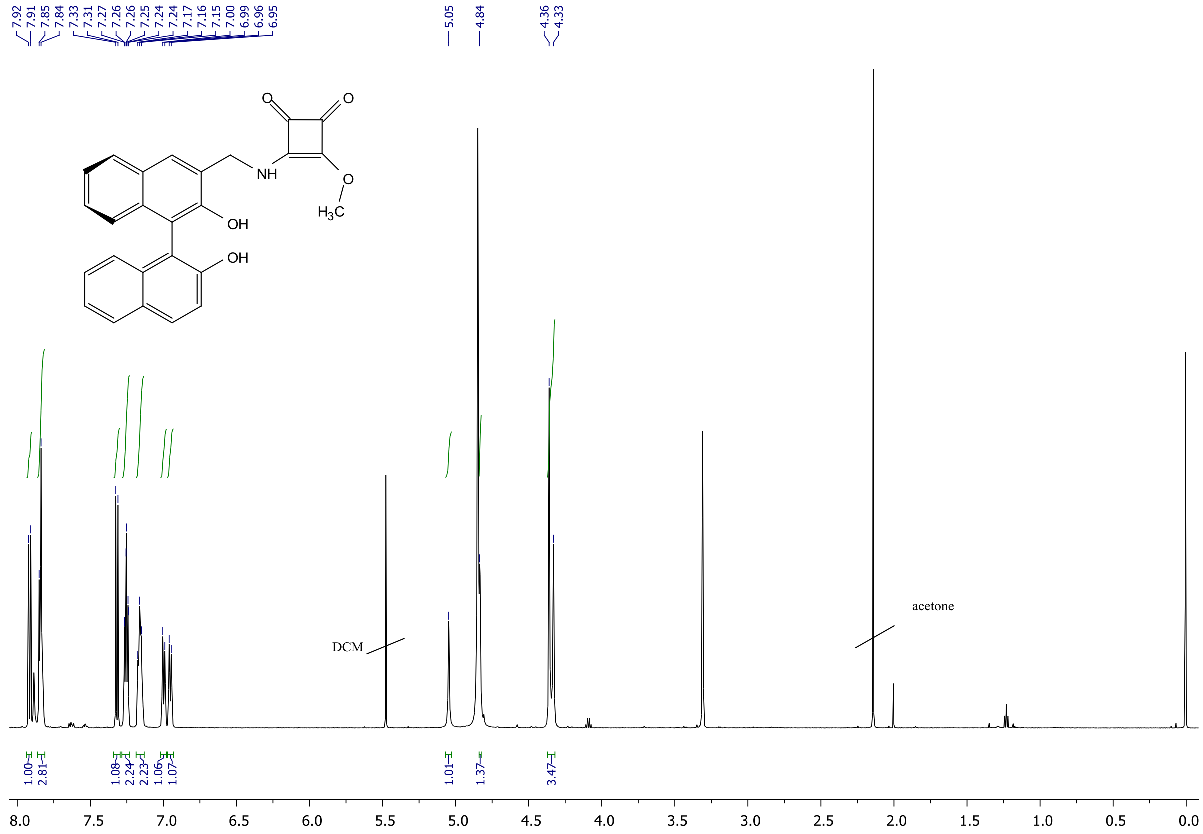

^{13}C NMR spectrum of **11**

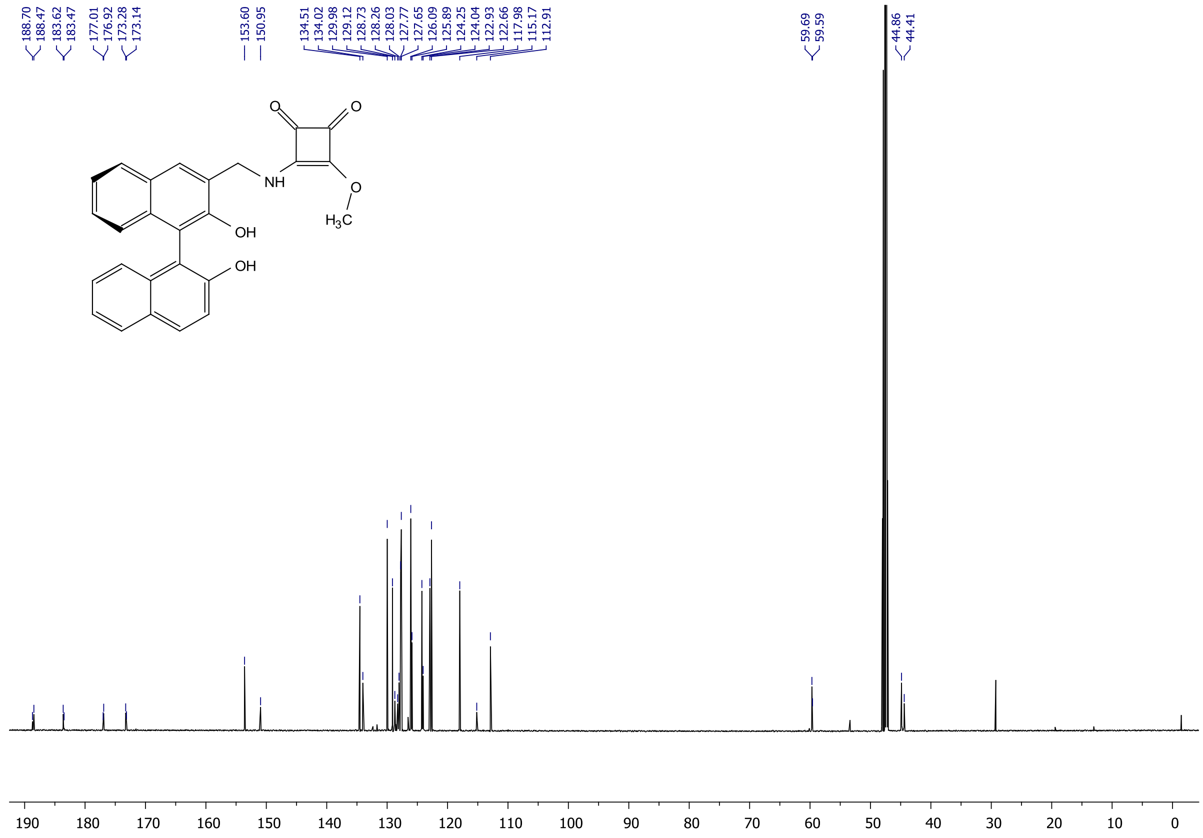

^{1}H NMR spectrum of (S_a,S,S)-**C8**

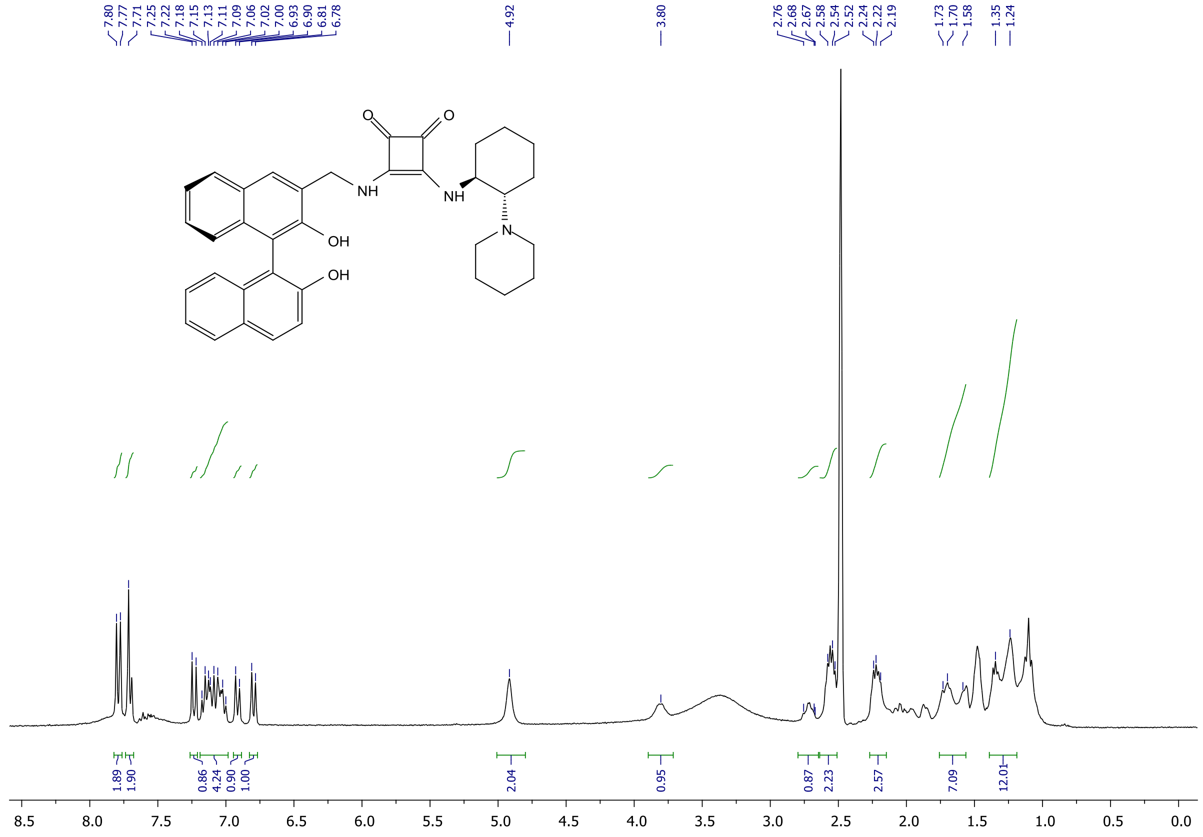

^{13}C NMR spectrum of (S_a,S,S)-**C8**

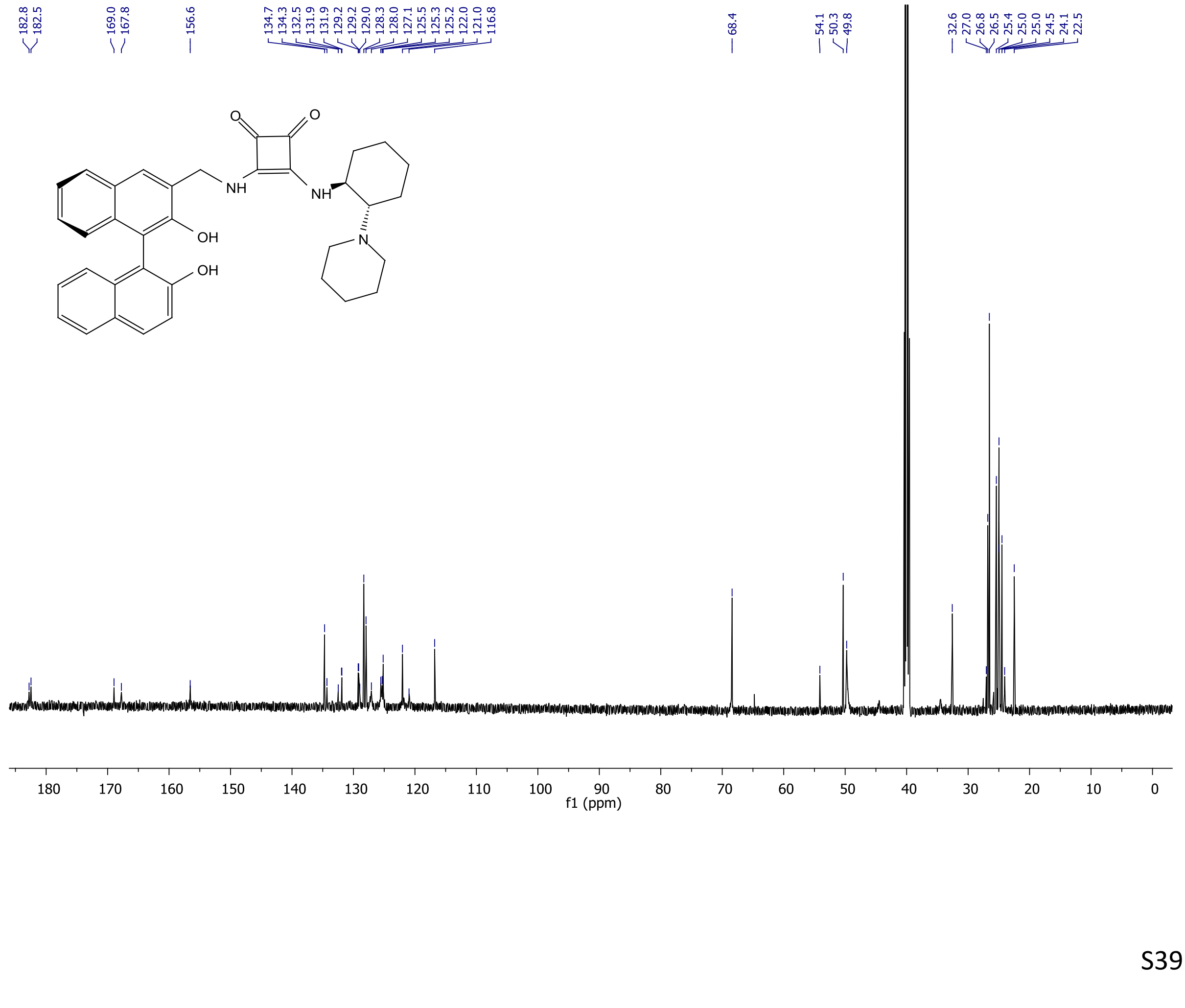

^{1}H NMR spectrum of (S_a,*R*,*R*)-**C8**

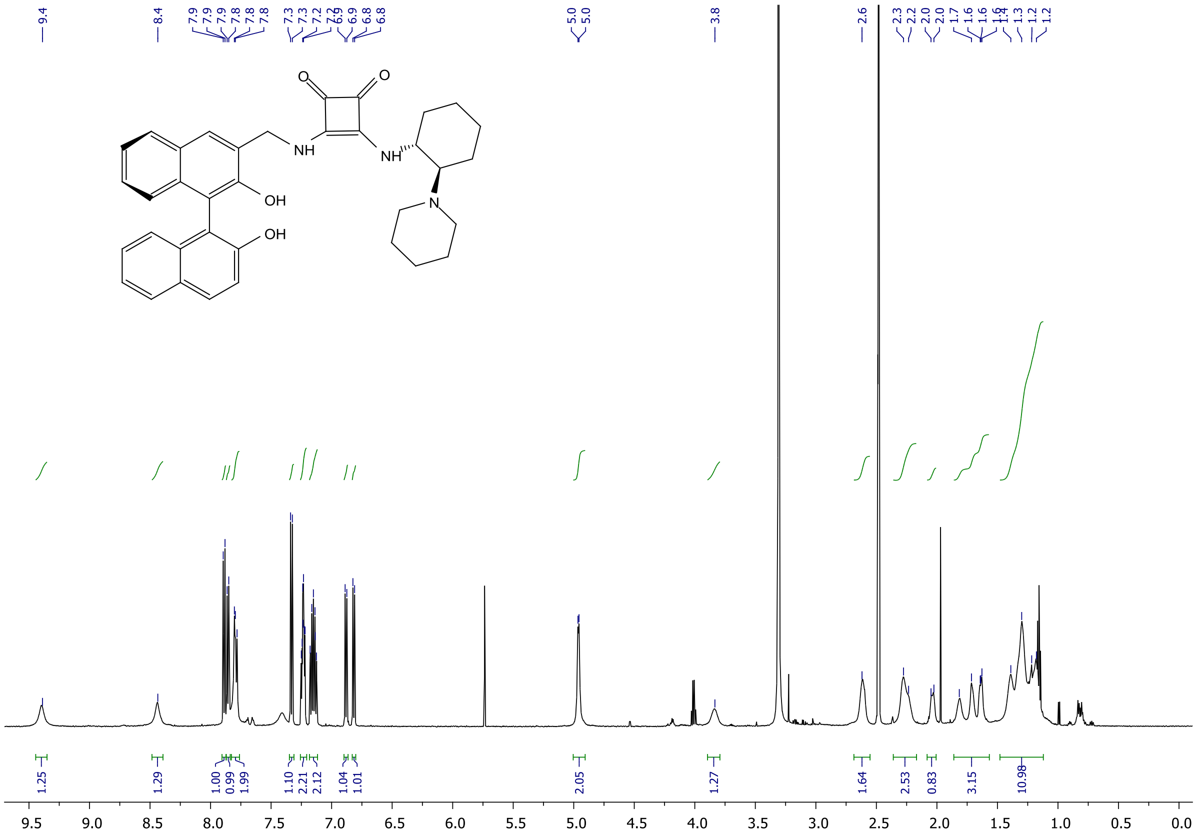

^{13}C NMR spectrum of (S_a,*R*,*R*)-**C8**

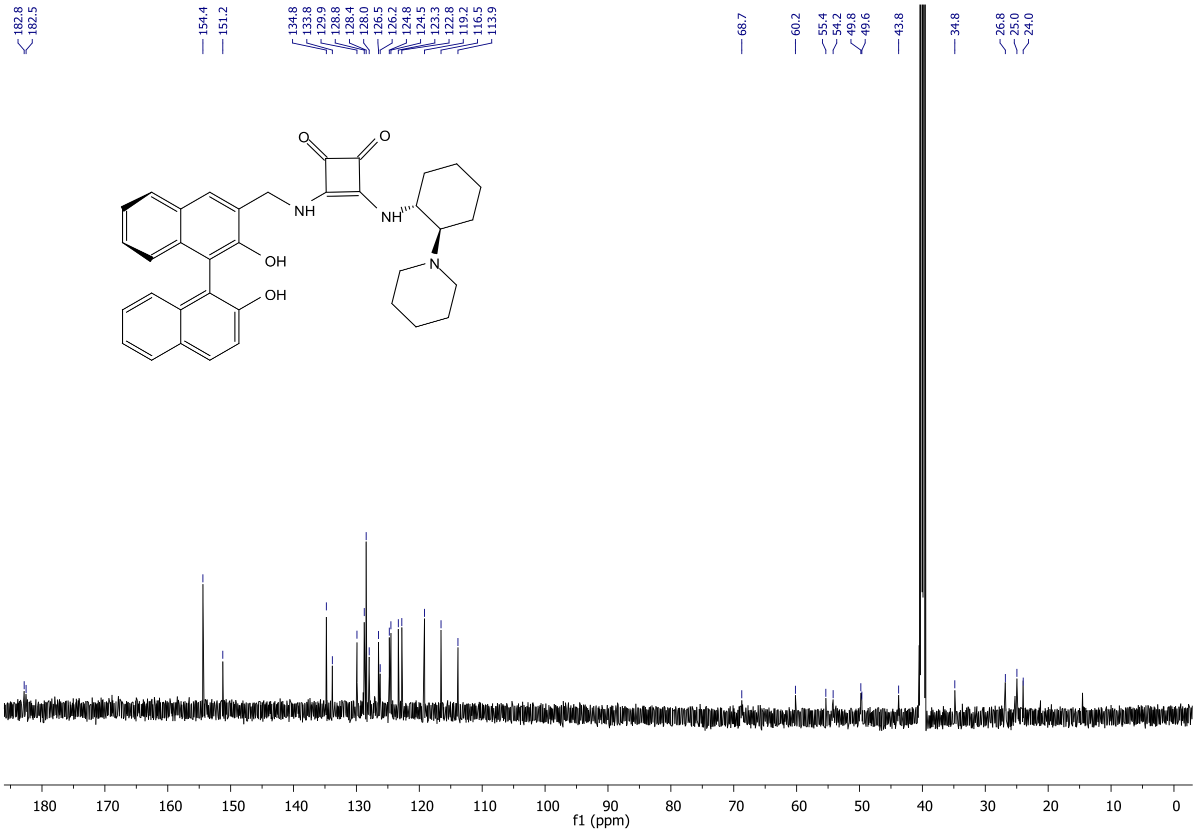

HPLC chromatograms

Racemic standard of the major diastereomer

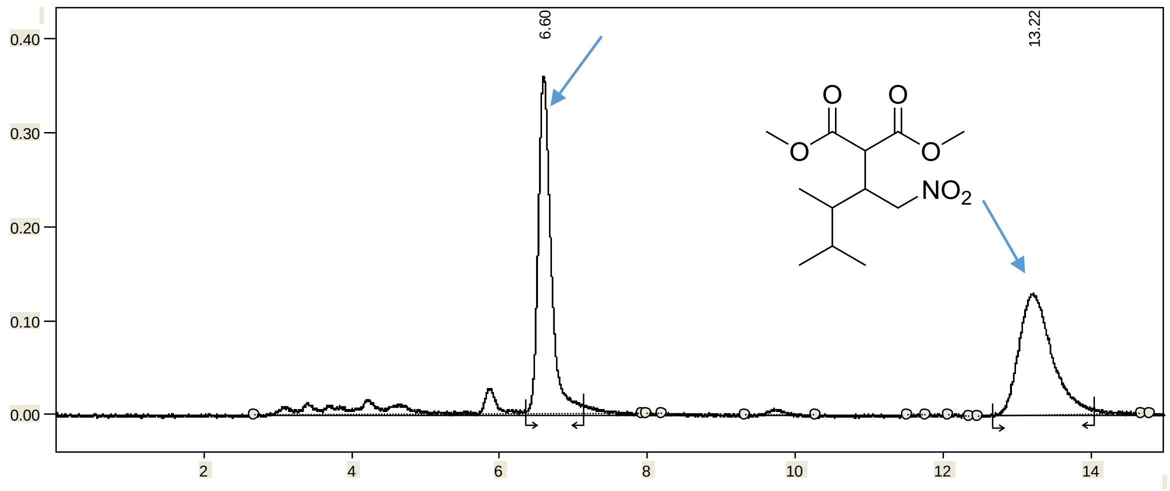

Time [min.] Area[%]
6.60 51.96
13.22 48.04

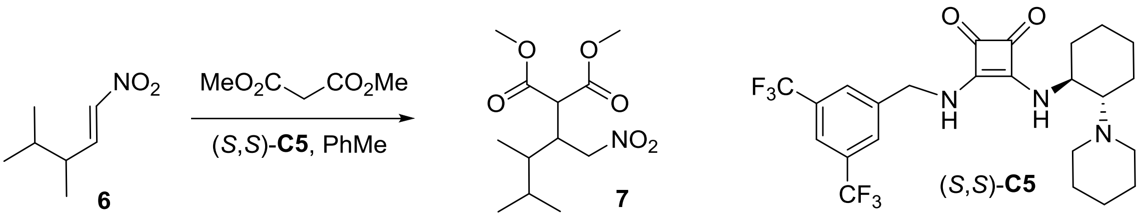

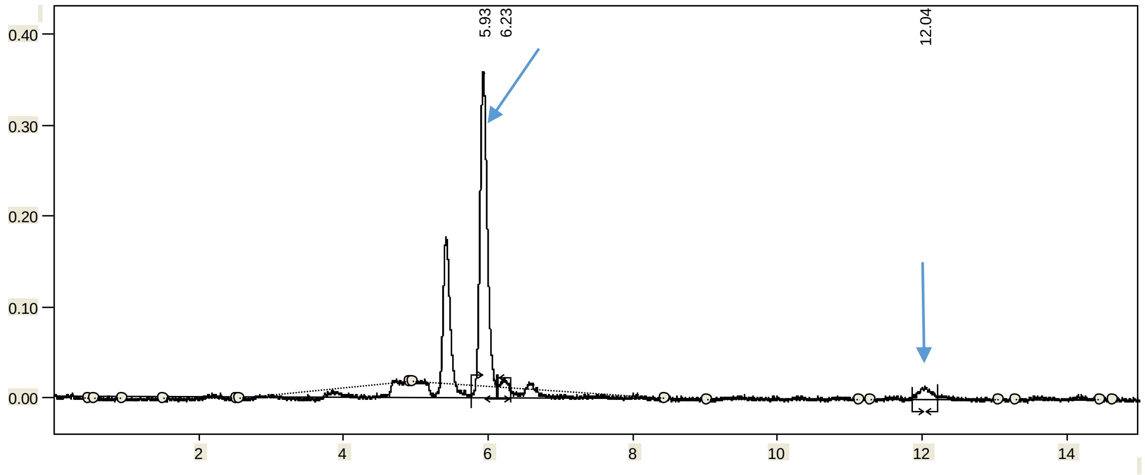

Time [min.] Area[%]
5.93 97.64
6.23 2.36

Racemic standard of the minor diastereomer

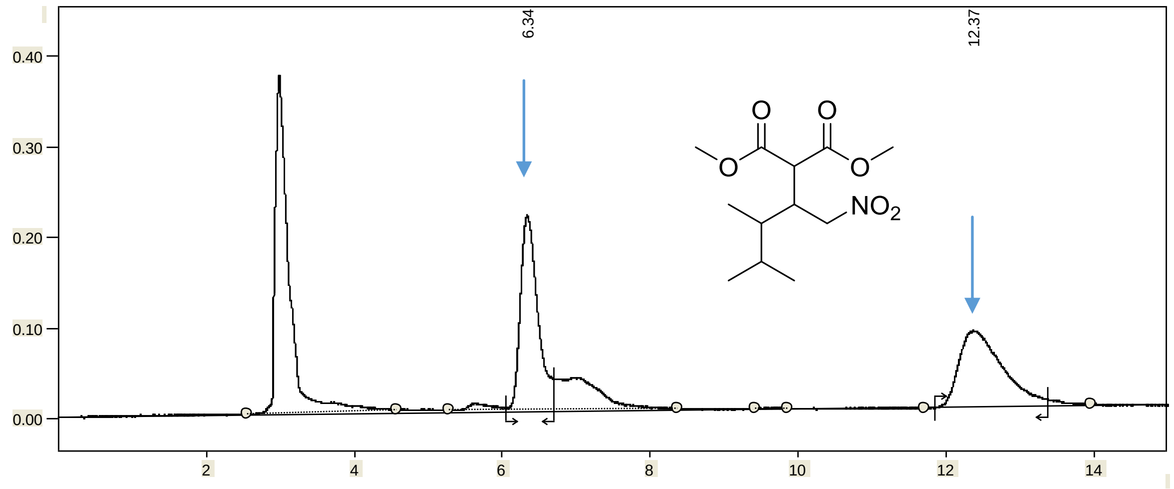

Time [min.] Area[%]
6.34 50.66
12.37 49.34

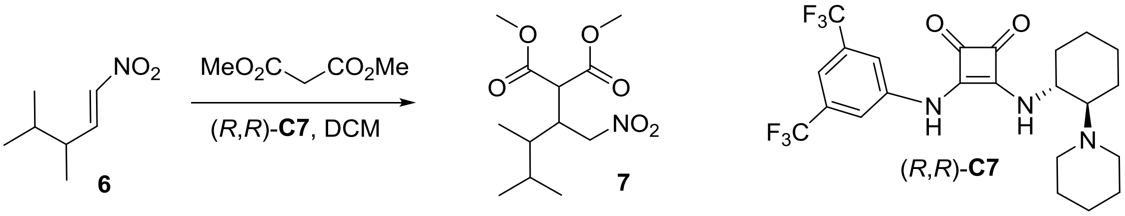

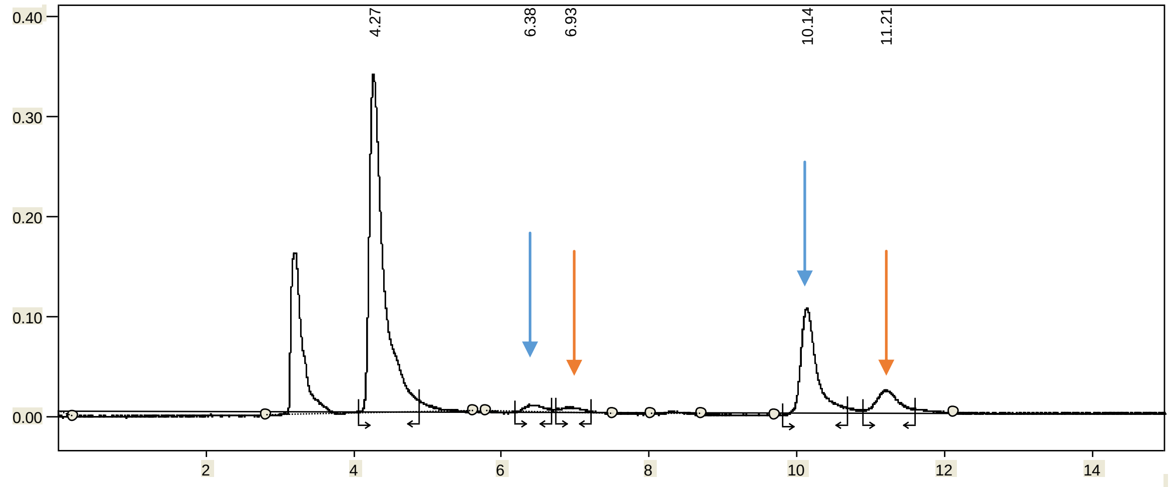

Time [min.] Area[%]
4.27 66.43 (nitroalkene)
6.38 2.00
6.93 1.63
10.14 22.83
11.21 7.11

Racemic nitroalkene and recovered material after Michael addition

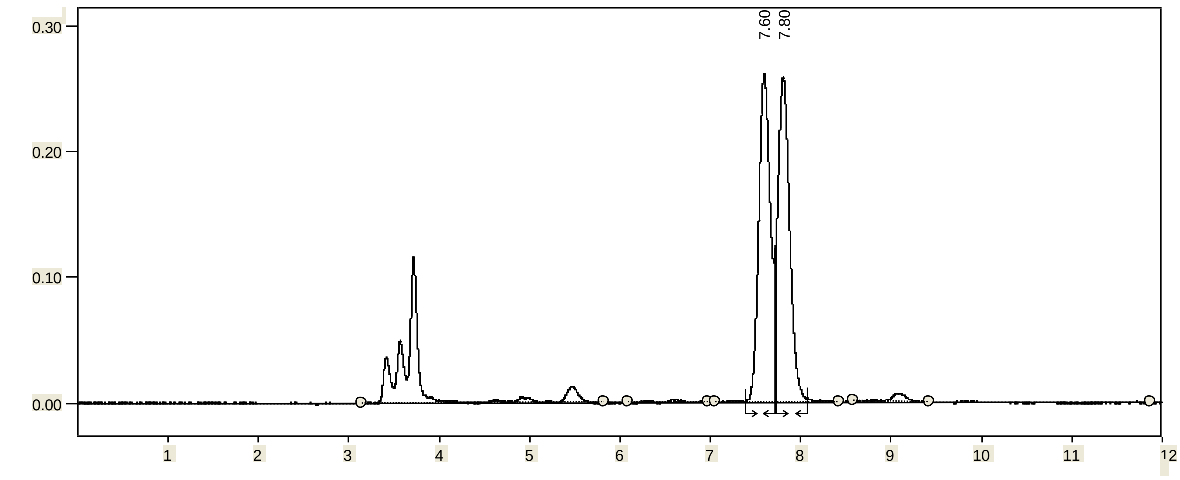

Time [min.] Area[%]
7.60 51.04
7.80 48.96

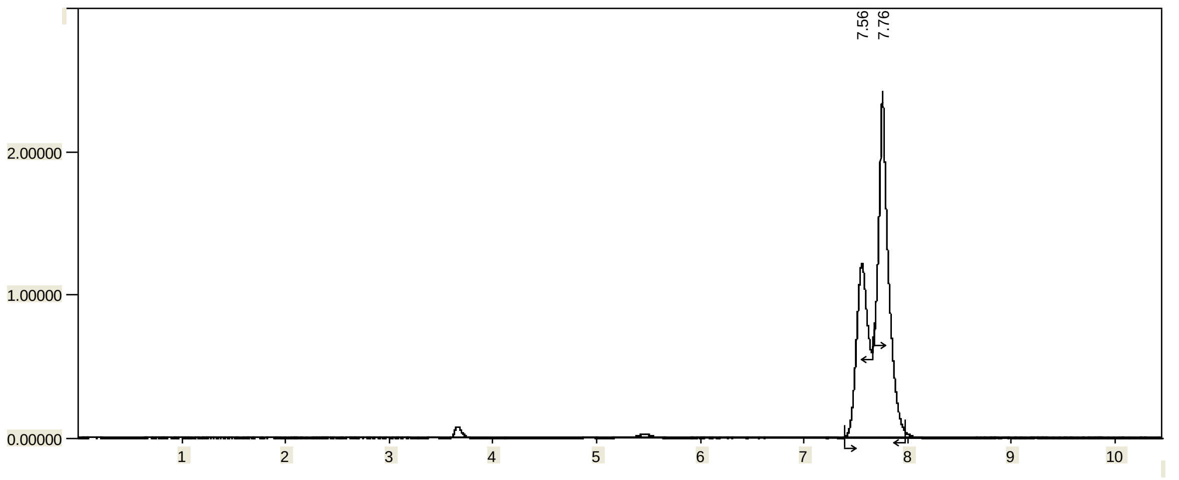

Time [min.] Area[%]
7.56 33.32
7.76 66.68

Conditions: Daicel IC, 245 nm, 1mL/min, hex/*i*PrOH 99:1

Computational details

Structures were first pre-optimized at the semiempirical AM1 method followed by geometrical optimization at the HF/6-31G* level using the Spartan 16 program package.[21] Energies of transition states were further refined by single point calculations at the M06-2X/6-311+G** level of theory as implemented in Spartan 16 package.[22] Solvation effects were evaluated by single point energy calculations using conductor-like C-PCM solvation model with toluene and THF as solvents. The transition states were characterized by frequency calculation at the HF/6-31G* level. All transition states have just one imaginary vibration corresponding to C–C bond formation (verified by visualization).

TS-RR

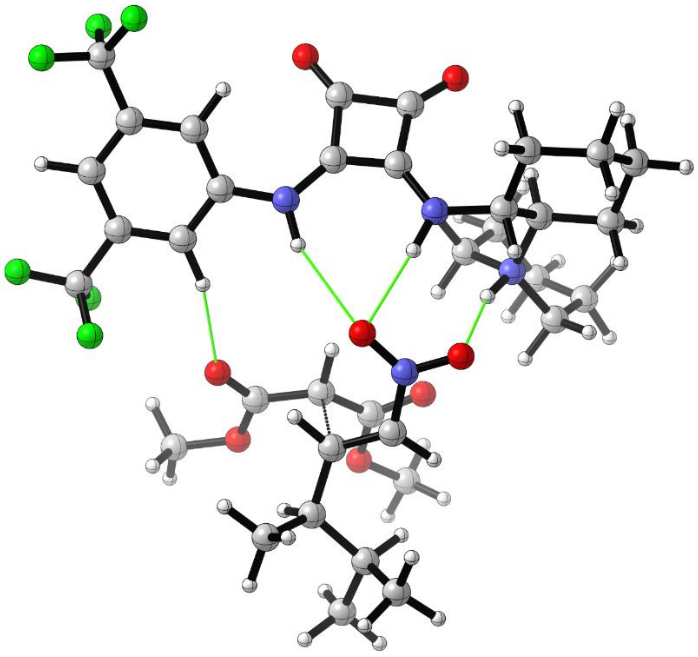

[21] Spartan 16, Wavefunction Inc.,Irvine CA

[22] Shao, Y.; Molnar, L. F.; Jung, Y.; Kussmann, J.; Ochsenfeld, C.; Brown, S. T.; Gilbert, A. T. B.; Slipchenko, L. V.; Levchenko, S. V.; ONeill, D. P.; Jr, R. A. D.; Lochan, R. C.; Wang, T.; Beran, G. J. O.; Besley, N. A.; Herbert, J. M.; Lin, C. Y.; Voorhis, T. V.; Chien, S. H.; Sodt, A.; Steele, R. P.; Rassolov, V. A.; Maslen, P. E.; Korambath, P. P.; Adamson, R. D.; Austin, B.; Baker, J.; Byrd, E. F. C.; Dachsel, H.; Doerksen, R. J.; Dreuw, A.; Dunietz, B. D.; Dutoi, A. D.; Furlani, T. R.; Gwaltney, S. R.; Heyden, A.; Hirata, S.; Hsu, C.-P.; Kedziora, G.; Khalliulin, R. Z.; Klunzinger, P.; Lee, A. M.; Lee, M. S.; Liang, W.; Lotan, I.; Nair, N.; Peters, B.; Proynov, E. I.; Pieniazek, P. A.; Rhee, Y. M.; Ritchie, J.; Rosta, E.; Sherrill, C. D.; Simmonett, A. C.; Subotnik, J. E.; Woodcock III, H. L.; Zhang, W.; Bell, A. T.; Chakraborty, A. K. *Phys. Chem. Chem. Phys*. **2006**, *8*, 3172 - 3191.

Cartesian coordinates

C	-0.239268	-3.046376	-0.433477
C	-1.426529	-2.387676	-0.202291
C	-0.580358	-4.130840	0.493677
C	-1.940111	-3.479295	0.658549
N	0.863598	-2.721224	-1.135213
N	-1.872468	-1.181535	-0.609077
O	-2.944916	-3.733325	1.232605
O	-0.001931	-5.060125	0.968297
H	0.850911	-1.852260	-1.628583
H	-1.190994	-0.563434	-0.998715
C	-3.146672	-0.623492	-0.378699
C	-5.639402	0.555511	-0.012141
C	-3.250954	0.752686	-0.244932
C	-4.289638	-1.412716	-0.337009
C	-5.520760	-0.819247	-0.137774
C	-4.499022	1.332210	-0.073691
C	2.041173	-3.546057	-1.335832
C	4.245064	-4.381553	-0.476161
C	2.980479	-5.736520	-2.192454
C	3.907964	-5.789598	-0.980476
C	1.711951	-4.948083	-1.866907
C	2.965594	-3.628865	-0.093629
H	2.599100	-3.023203	-2.101753
H	1.097934	-4.830631	-2.753569
H	2.717085	-6.740488	-2.508372
H	3.501087	-5.271500	-3.027398
H	4.831157	-6.302921	-1.229269
H	4.770318	-3.844298	-1.261036
H	4.916414	-4.457840	0.367612
H	-2.371418	1.371367	-0.254905
H	-6.601681	1.007593	0.119674
H	-4.223061	-2.474805	-0.446845
H	2.459610	-4.164896	0.695267
H	3.436640	-6.358066	-0.182569
C	-4.591546	2.828659	0.076878
C	-6.752574	-1.676606	-0.028592
F	-6.616240	-2.825109	-0.669805
F	-7.038421	-1.965053	1.230768
F	-7.815966	-1.067618	-0.532075
F	-5.844023	3.252418	-0.029627
F	-3.888442	3.458145	-0.846478
F	-4.149029	3.233956	1.254805
N	3.200619	-2.212251	0.436613
C	2.298510	-1.859500	1.596990
C	4.605270	-1.748094	0.741056
C	2.805075	-2.422021	2.916991
C	4.204018	-1.896522	3.228512
H	5.234074	-2.032282	-0.086588
H	4.530375	-0.670154	0.755845
H	4.573064	-2.322293	4.155385
H	4.163810	-0.819187	3.354427
H	2.277022	-0.780693	1.630768
H	1.317109	-2.227913	1.362936
H	2.099665	-2.121817	3.684215
H	2.800290	-3.508780	2.901092
H	2.921682	-1.598290	-0.319811
H	1.127511	-5.489749	-1.134055
C	5.146590	-2.244868	2.077834
H	5.317097	-3.315521	2.057929
H	6.116044	-1.778150	2.219731
C	1.698306	2.732334	-1.419367
C	2.546383	1.699750	-1.757969
N	2.063578	0.432797	-1.834683
O	2.867460	-0.497512	-1.938959
O	0.864058	0.199966	-1.790263
H	3.609878	1.744767	-1.769301
C	2.095630	4.196456	-1.452117
H	1.688689	4.637838	-0.553396
C	3.619185	4.480899	-1.437486
H	4.077727	3.745281	-0.782990
C	4.296604	4.382048	-2.811873
H	4.070288	3.454673	-3.325340
H	5.375007	4.443058	-2.700020
H	3.993244	5.197963	-3.460059
C	3.899078	5.853265	-0.813042
H	3.514618	5.904657	0.199858
H	3.444778	6.655648	-1.387455
H	4.967377	6.045718	-0.777034
H	0.647861	2.549491	-1.508635
C	0.082530	3.213673	0.992324
C	2.458875	2.278186	1.471347
C	1.180498	2.296014	0.834433
O	3.187216	1.299538	1.465871
O	-0.995892	3.017617	0.478180
O	2.876694	3.418905	2.019591
O	0.316527	4.334875	1.669965
H	0.842933	1.321118	0.546016
C	1.349101	4.843226	-2.636820

H	0.276961	4.763475	-2.493344	H	4.151278	2.689642	3.464358
H	1.596995	4.372649	-3.581717	C	-0.761885	5.239713	1.788804
H	1.592203	5.897044	-2.712255	H	-1.614120	4.766750	2.254181
C	4.131637	3.408735	2.656666	H	-1.059203	5.615776	0.819592
H	4.270418	4.404752	3.049986	H	-0.398433	6.048471	2.405239
H	4.923947	3.174134	1.958925				

Geometrical optimization: HF/6-31G*
Imaginary frequency: -410 cm^{-1}
Single point energy calculation: M06-2X/6-311+G**
SCF total energy: -2780.888551 hartrees
C-PCM (THF): -2780.928602 hartrees; C-PCM (toluene): -2780.914155 hartrees

TS-RS

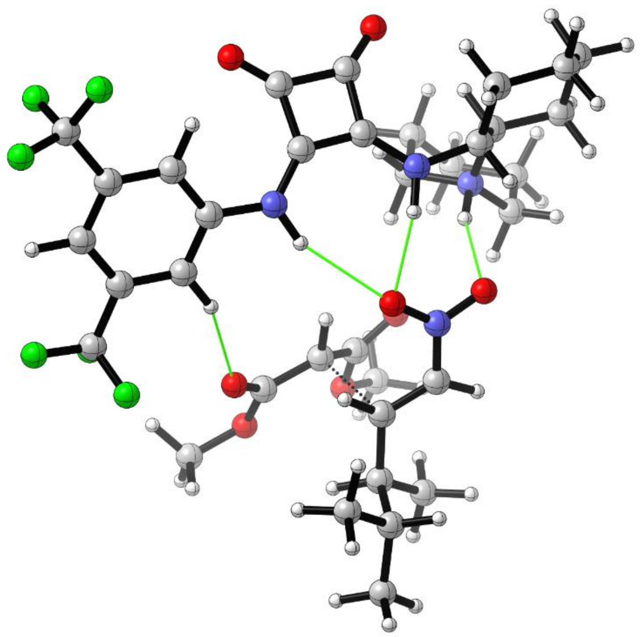

Cartesian coordinates

C	0.649683	-2.940782	-0.511305	C	-5.011029	-2.164081	-0.067892
C	-0.660521	-2.612380	-0.241419	C	-4.583041	0.172378	0.116440
C	0.606664	-4.119536	0.360847	C	2.965840	-2.815037	-1.453965
C	-0.867882	-3.834497	0.571218	C	5.326106	-3.109412	-0.657843
N	1.625220	-2.318404	-1.200525	C	4.405898	-4.647583	-2.440236
N	-1.405348	-1.541297	-0.585896	C	5.345482	-4.527099	-1.242075
O	-1.764526	-4.355023	1.144596	C	2.986618	-4.224727	-2.060132
O	1.407112	-4.897714	0.781559	C	3.905639	-2.731817	-0.223634
H	1.387532	-1.458580	-1.651788	H	3.362008	-2.129581	-2.191756
H	-0.912632	-0.753542	-0.953167	H	2.343541	-4.220453	-2.933895
C	-2.775686	-1.341515	-0.317182	H	4.398036	-5.667637	-2.809868
C	-5.479615	-0.876979	0.143288	H	4.772220	-4.022648	-3.252445
C	-3.230885	-0.049831	-0.103268	H	6.362390	-4.773811	-1.529697
C	-3.673875	-2.402675	-0.313435	H	5.681231	-2.413220	-1.412535

H	6.012227	-3.059671	0.176091
H	-2.545896	0.780056	-0.094708
H	-6.522339	-0.700370	0.314100
H	-3.336131	-3.402242	-0.488034
H	3.569352	-3.426080	0.531696
H	5.053758	-5.239656	-0.474495
C	-5.057344	1.583021	0.353512
C	-5.976297	-3.316242	-0.000152
F	-5.550199	-4.362041	-0.687781
F	-6.169566	-3.720060	1.244824
F	-7.164861	-2.984273	-0.482713
F	-6.381535	1.655505	0.375489
F	-4.638776	2.405735	-0.589684
F	-4.628220	2.057022	1.511094
N	3.777372	-1.337991	0.395012
C	2.858177	-1.312462	1.594254
C	5.016726	-0.529101	0.695242
C	3.551044	-1.784772	2.863958
C	4.763730	-0.911908	3.176602
H	5.665917	-0.589288	-0.162686
H	4.655478	0.486363	0.774739
H	5.269104	-1.266856	4.068331
H	4.435113	0.104896	3.367918
H	2.538492	-0.286361	1.695555
H	2.009595	-1.927685	1.358845
H	2.821328	-1.734082	3.664761
H	3.844360	-2.827956	2.778633
H	3.317901	-0.778454	-0.313153
H	2.573599	-4.933859	-1.353799
C	5.722338	-0.927605	1.987254
H	6.177607	-1.907650	1.899023
H	6.532471	-0.220928	2.136177
C	1.055465	3.158259	-1.317567
C	2.159008	2.407183	-1.655121
N	2.037986	1.059320	-1.773669
O	3.060932	0.381279	-1.885683
O	0.942517	0.514229	-1.760589
H	3.169130	2.746268	-1.651252
C	1.076064	4.670824	-1.335868
H	0.300146	5.005417	-0.658107
C	0.682233	5.164410	-2.762281
H	1.482201	4.875922	-3.441370
C	-0.622686	4.533067	-3.269160
H	-0.520809	3.471241	-3.462094
H	-0.926063	4.998459	-4.201057
H	-1.429034	4.664971	-2.553066
C	0.556844	6.693038	-2.798447
H	1.489571	7.192182	-2.566478
H	-0.194563	7.038399	-2.093083
H	0.253350	7.019152	-3.787915
H	0.096337	2.701403	-1.420587
C	-0.672106	3.185785	1.065999
C	1.813455	2.720931	1.664543
C	0.605706	2.540284	0.921428
O	2.744967	1.934610	1.610708
O	-1.652811	2.803563	0.464985
O	1.920177	3.833096	2.389729
O	-0.726106	4.273091	1.829951
H	0.505591	1.543325	0.542640
C	2.410734	5.261453	-0.866650
H	3.191761	5.130140	-1.610176
H	2.734184	4.801254	0.056747
H	2.312320	6.322501	-0.677673
C	3.104589	4.016146	3.126290
H	2.987420	4.957132	3.642822
H	3.968562	4.057384	2.476893
H	3.243215	3.218950	3.844248
C	-1.983114	4.906347	1.944005
H	-2.730627	4.220026	2.313371
H	-2.309224	5.294064	0.988516
H	-1.839808	5.717146	2.642729

Geometrical optimization: HF/6-31G*
Imaginary frequency: -382 cm^{-1}
Single point energy calculation: M06-2X/6-311+G**
SCF total energy: -2780.889209 hartrees
C-PCM (THF): -2780.929827 hartrees; C-PCM (toluene): -2780.915161 hartrees

TS-SR

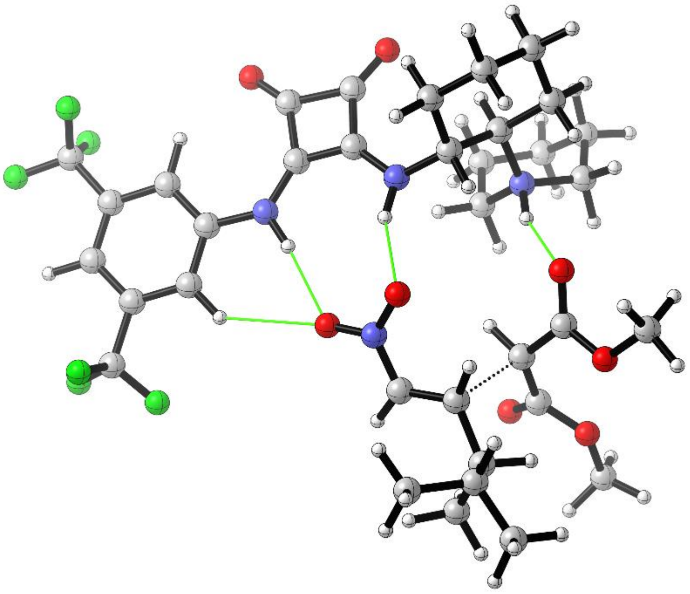

Cartesian coordinates

C	2.854021	2.554229	-0.564001	N	-2.660923	-0.377309	-0.327242
C	1.508289	2.642746	-0.219559	O	-4.108928	-3.371942	0.201521
N	0.656506	1.679769	-0.640558	O	-1.206660	-4.731076	-0.165223
O	-0.508044	1.692600	-0.252149	H	0.156571	-0.846341	-1.089205
O	1.042563	0.790531	-1.399094	H	-1.888760	0.255311	-0.439319
C	3.809182	3.728491	-0.426140	C	-3.896317	0.251402	-0.120158
C	4.046264	4.354140	-1.833856	C	-6.271872	1.670806	0.252815
H	1.087774	3.322832	0.484956	C	-3.880808	1.645469	-0.024458
H	3.069643	1.900045	-1.382139	C	-5.101650	-0.422418	-0.030321
H	4.765178	3.315625	-0.135549	C	-6.275604	0.297083	0.158784
H	4.323377	3.527424	-2.485887	C	-5.056695	2.337165	0.157423
C	2.821487	5.036202	-2.453992	C	1.161506	-2.610767	-1.474805
C	5.239055	5.317634	-1.814719	C	2.968941	-4.358731	-1.240766
H	6.118201	4.853175	-1.377956	C	1.773847	-4.125299	-3.440795
H	5.492489	5.621663	-2.825870	C	2.422395	-5.097007	-2.464300
H	5.020668	6.219116	-1.251529	C	0.668496	-3.355670	-2.728395
H	1.967366	4.372025	-2.511164	C	1.882469	-3.568824	-0.488275
H	3.053142	5.364895	-3.462766	H	1.891025	-1.872525	-1.785281
H	2.523473	5.913618	-1.889112	H	0.226601	-2.615181	-3.386503
C	-1.141296	-2.304638	-0.583029	H	1.362455	-4.656359	-4.293002
C	-2.346584	-1.686888	-0.318168	H	2.521985	-3.436672	-3.827937
C	-1.662923	-3.630467	-0.259552	H	3.238585	-5.635305	-2.935652
C	-3.017449	-2.985333	-0.056014	H	3.750377	-3.672654	-1.557962
N	0.082732	-1.826617	-0.887495	H	3.423943	-5.086913	-0.583224

H	-2.947038	2.170824	-0.087411
H	-7.183290	2.214902	0.399107
H	-5.141957	-1.491212	-0.093254
H	1.168730	-4.249499	-0.052799
H	1.694169	-5.840060	-2.148781
C	-5.046179	3.840929	0.210870
C	-7.562826	-0.473444	0.285795
F	-7.693710	-1.365178	-0.682418
F	-7.622293	-1.135891	1.429799
F	-8.621990	0.318270	0.238250
F	-5.905114	4.301347	1.107220
F	-5.390744	4.370452	-0.954094
F	-3.855373	4.322509	0.519192
N	2.516977	-2.774912	0.660141
C	1.565468	-2.285448	1.727192
C	3.672726	-3.428375	1.366765
C	1.060715	-3.416797	2.615836
C	2.215403	-4.167668	3.279489
H	4.416762	-3.693257	0.637782
H	4.099201	-2.649571	1.985098
H	1.842285	-5.027805	3.823791
H	2.701392	-3.519268	4.004567
H	2.130866	-1.570411	2.310434
H	0.766074	-1.761907	1.243062
H	0.416709	-2.971413	3.366692
H	0.441620	-4.101125	2.046448

H	2.938000	-1.952541	0.223828
H	-0.117063	-4.048311	-2.447531
C	3.236127	-4.606697	2.228579
H	2.817391	-5.394544	1.610258
H	4.121689	-5.018768	2.701371
C	4.137530	1.667742	1.997332
C	4.537380	0.197486	-0.066648
C	3.687999	0.983270	0.793560
O	4.203015	-0.877606	-0.545415
O	3.410409	1.901718	2.925157
O	5.697886	0.734284	-0.401620
O	5.416473	2.048023	2.004700
H	2.754456	0.488177	0.975275
C	3.385644	4.741160	0.644600
H	4.169593	5.472256	0.804116
H	3.197803	4.250871	1.591682
H	2.485887	5.278040	0.367413
C	6.542725	0.000254	-1.262529
H	7.426098	0.606322	-1.390864
H	6.066070	-0.170755	-2.217099
H	6.808382	-0.950884	-0.822667
C	5.874356	2.714211	3.162071
H	6.920343	2.918945	2.988822
H	5.757267	2.090949	4.037389
H	5.335803	3.638243	3.316109

Geometrical optimization: HF/6-31G*
Imaginary frequency: -493 cm^{-1}
Single point energy calculation: M06-2X/6-311+G**
SCF total energy: -2780.889817 hartrees
C-PCM (THF): -2780.932128 hartrees; C-PCM (toluene): -2780.917016 hartrees

TS-SS

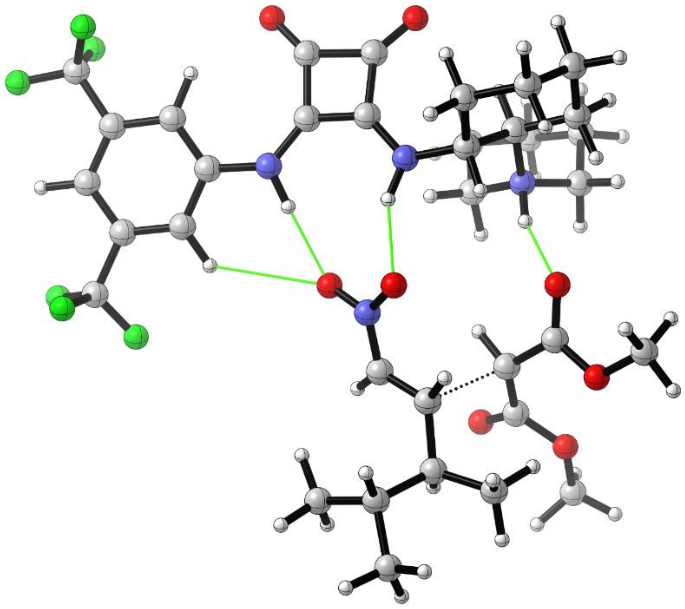

Cartesian coordinates

C	2.877123	2.548235	-0.700348	C	-6.197906	1.709860	0.242897
C	1.590126	2.454680	-0.166781	C	-3.806358	1.608987	-0.003789
N	0.793343	1.437100	-0.537533	C	-5.099505	-0.411863	-0.110631
O	-0.291418	1.281204	0.031688	C	-6.248955	0.341231	0.093423
O	1.133777	0.654119	-1.426838	C	-4.959223	2.335587	0.190508
C	3.683564	3.839972	-0.645627	C	1.156034	-2.742086	-1.433992
C	2.936710	4.965951	-1.448056	C	2.940060	-4.461435	-0.916973
H	1.235579	3.015053	0.667642	C	1.916611	-4.412246	-3.213580
H	3.056949	1.940189	-1.563498	C	2.481335	-5.298968	-2.112321
H	2.562797	4.522974	-2.370010	C	0.761006	-3.591371	-2.655477
C	1.746627	5.572885	-0.691466	C	1.810354	-3.602326	-0.319250
C	3.877512	6.116093	-1.843214	H	1.897144	-2.018383	-1.751410
H	4.659676	5.807320	-2.524051	H	0.372851	-2.908649	-3.403641
H	3.308549	6.896159	-2.338035	H	1.571934	-5.012479	-4.049382
H	4.346237	6.559411	-0.968286	H	2.695803	-3.755515	-3.595313
H	0.950486	4.865520	-0.518946	H	3.326745	-5.876643	-2.472543
H	1.327239	6.394724	-1.263019	H	3.752880	-3.809770	-1.227794
H	2.063173	5.970843	0.269343	H	3.332153	-5.134458	-0.166449
C	-1.190094	-2.397922	-0.667037	H	-2.852171	2.100574	-0.029613
C	-2.381198	-1.759238	-0.388741	H	-7.091767	2.279857	0.398870
C	-1.737453	-3.712933	-0.329371	H	-5.174725	-1.475521	-0.216488
C	-3.080844	-3.040015	-0.138874	H	1.066524	-4.239250	0.131828
N	0.028930	-1.936803	-0.992439	H	1.724877	-6.011075	-1.792181
N	-2.656794	-0.439231	-0.377976	C	-4.894958	3.834579	0.303927
O	-4.184023	-3.403638	0.105791	C	-7.565594	-0.383907	0.183094
O	-1.296470	-4.816379	-0.204597	F	-7.692321	-1.285440	-0.775945
H	0.130707	-0.956450	-1.179565	F	-7.689999	-1.024957	1.334157
H	-1.853725	0.162048	-0.420059	F	-8.594044	0.443160	0.085621
C	-3.869500	0.222120	-0.157636	F	-5.752349	4.291947	1.202714

F	-5.197048	4.421656	-0.845004
F	-3.692556	4.258529	0.651346
N	2.380008	-2.704762	0.787128
C	1.376376	-2.056503	1.712160
C	3.436614	-3.325251	1.659279
C	0.729078	-3.065912	2.653525
C	1.776839	-3.802533	3.489756
H	4.228057	-3.695106	1.033511
H	3.845954	-2.504795	2.233620
H	1.309149	-4.587230	4.073482
H	2.229855	-3.109676	4.194807
H	1.941534	-1.326295	2.276843
H	0.657846	-1.528445	1.118787
H	0.047456	-2.518504	3.295571
H	0.126182	-3.774865	2.096981
H	2.873591	-1.951277	0.304627
H	-0.045273	-4.261239	-2.379743
C	2.864850	-4.389246	2.588192
H	2.465492	-5.218838	2.012947
H	3.681405	-4.786803	3.182084
C	4.527572	1.891930	1.685824
C	4.686627	0.077139	-0.137093
C	3.955928	1.070328	0.622250
O	4.210976	-1.003445	-0.456183
O	3.852246	2.421220	2.526552
O	5.900705	0.399674	-0.542202
O	5.850733	2.047565	1.661404
H	3.020126	0.665138	0.948254
H	3.753540	4.164361	0.387588
C	5.093229	3.602204	-1.204234
H	5.573989	2.763334	-0.731655
H	5.723716	4.468051	-1.050192
H	5.052725	3.407680	-2.273590
C	6.615033	-0.540330	-1.315879
H	6.767656	-1.457010	-0.763429
H	7.565422	-0.077555	-1.531694
H	6.090924	-0.761261	-2.234729
C	6.411462	2.876483	2.656967
H	6.024800	3.883227	2.584365
H	7.476086	2.874114	2.476346
H	6.199616	2.490709	3.644046

Geometrical optimization: HF/6-31G*
Imaginary frequency: -469 cm^{-1}
Single point energy calculation: M06-2X/6-311+G**
SCF total energy: -2780.883700 hartrees
C-PCM (THF): -2780.925621 hartrees; C-PCM (toluene): -2780.910659 hartrees
